# Efficacy of mindfulness- and acceptance-based cognitive-behavioral therapies for bodily distress in adults: a meta-analysis

**DOI:** 10.3389/fpsyt.2023.1160908

**Published:** 2023-04-20

**Authors:** Frederic Maas genannt Bermpohl, Lea Hülsmann, Alexandra Martin

**Affiliations:** Department of Clinical Psychology and Psychotherapy, School of Human and Social Sciences, University of Wuppertal, Wuppertal, Germany

**Keywords:** persistent physical symptoms, somatoform disorders (MeSH), Somatic Symptom Disorder (SSD), treatment evaluation, efficacy

## Abstract

**Objective:**

Bodily distress, i.e., somatoform disorders and associated functional somatic syndromes, is highly prevalent, often persistent and highly disabling. It has been proposed that “third wave” therapies may be beneficial variants of cognitive behavioral treatments. However, evidence on their efficacy is scarce. This meta-analysis examines the efficacy of “third wave” psychotherapies (mindfulness-based cognitive therapy [MBCT], mindfulness-based stress reduction [MBSR], and acceptance and commitment therapy [ACT]) in adults with bodily distress.

**Method:**

We included randomized controlled trials (RCTs) treating adults with bodily distress using MBCT, MBSR, and ACT compared to inactive and non-specific control groups. A random effects model was used. The primary outcome was somatic symptom severity. Secondary outcomes were degrees of depression and of anxiety, health anxiety, perceived health status, mindfulness, psychological inflexibility, and pain acceptance.

**Results:**

Sixteen RCTs with 1,288 participants were included in the analysis (*k* = 4 MBCT, *k* = 7 MBSR, *k* = 5 ACT; *k* = 7 fibromyalgia, *k* = 5 irritable bowel syndrome, *k* = 1 chronic fatigue syndrome, *k* = 2 bodily distress, *k* = 1 medically unexplained symptoms). However, not all studies provided data for each of the relevant outcomes. The analyses revealed that “third wave“ therapies were more effective than control conditions in reducing somatic symptom severity (*k* = 15, *n* = 1,100, *g* = −0.51, 95%CI −0.69; −0.32). Heterogeneity was moderate (*I*^2^ = 52.8%, 95%CI 15.1 to 73.8). Effects for secondary outcomes were small to moderate with varying degrees of heterogeneity. We did not find differences between the different therapy approaches (mindfulness- vs. acceptance-based therapies); neither therapy dosis (i.e., total duration of therapy sessions) nor number of sessions were significant moderators of efficacy.

**Conclusions:**

The therapies addressing mindfulness and acceptance proved to be more effective than control conditions. Hence, they are promising treatment approaches for bodily distress. However, there is still need for research on which patient groups may benefit from these psychological approaches.

**Systematic review registration:**

https://osf.io/g7hby, identifier: 10.17605/OSF.IO/4RZGC.

## 1. Introduction

Incapacitating, persistent physical symptoms, i.e., functional and somatoform bodily complaints, are highly prevalent. Due to an increased use of healthcare services and frequent work absenteeism these complaints are often associated with increased costs for the health care system (1). Several psychological therapies are considered well-established in the treatment of persistent physical symptoms; with the largest body of evidence available for cognitive behavioral therapy [CBT, (2)]. However, symptoms are at best moderately improved by psychotherapy (1). Hence, there is need for more effective treatments (3). So-called “third wave“ therapies may represent promising new approaches in this context. They may hold the potential to improve management of bodily distress emphasizing mindfulness and acceptance as important change mechanisms (4). The most prominent “third wave“ therapies are mindfulness-based cognitive therapy (MBCT), mindfulness-based stress reduction (MBSR), and acceptance and commitment therapy (ACT).

MBCT combines CBT techniques with mindfulness practices. Therefore, it helps individuals to observe their experiences including thoughts, physical sensations and emotions in a non-judgmental way, leading to greater acceptance of inner experiences (5). In its mindfulness practices, it is based on MBSR. MBSR combines mindfulness practices with yoga and body awareness techniques to help individuals manage stress, pain, and illness. The goal of MBSR is to help individuals enhance their self-awareness (6). Mindfulness, as a supposed key mechanism of MBCT and MBSR, may help patients to interrupt rumination processes, and as a consequence interpret bodily symptoms less negatively by shifting their attentional focus away from the negative appraisal of their symptoms. Consequently, mindfulness could indirectly lead to a more accepting attitude toward bodily sensations. ACT focuses on accepting difficult thoughts and emotions while working toward one's values and goals. The goal in ACT is to help individuals develop psychological flexibility and become more effective in their actions and relationships. To that effect, ACT rather aims at reducing avoidance behaviors and uses a more direct approach on acceptance of inner experiences including somatic symptoms, emphasizing mindfulness and values clarification as key components for psychological flexibility and well-being (7). Hence, all of these three “third wave” therapies could have a positive impact on emotion regulation processes and interrupt patients' reinforcement processes related to their symptoms (8), thus also reducing comorbid depression and anxiety (4). Efficacy of these therapies has already been shown for some specific functional somatic syndromes or symptoms [e.g., (9, 10)]. However, no meta-analysis in the field provides sufficient evidence for “third wave“ therapies taking into account recent changes in classification. Due to these changes, the focus in defining bodily distress is no longer primarily on the strict distinction between medically unexplained symptoms and medically explained symptoms, but on considering excessive psychobehavioral characteristics in relation to the physical symptoms. Hence, several different diagnoses can now be subsumed under the concept of bodily distress, such as medically unexplained symptoms, somatoform disorders, Bodily Distress Disorder, Somatic Symptom Disorder (and its predecessors in previous editions of the DSM). Functional somatic syndromes, such as irritable bowel syndrome, chronic fatigue syndrome, and fibromyalgia are also included here (3).

The research base for “third wave“ therapies has improved in recent years (11). Two recent reviews, one systematic review on mindfulness-based therapies in medically unexplained symptoms (9) and one narrative review on “third wave“ therapies in bodily distress and health anxiety (4), yield promising results. However, effect sizes were not statistically aggregated.

The last meta-analysis focussing on the efficacy of mindfulness-based therapies, including MBCT, MBSR, and unspecified mindfulness training for bodily distress (12), incorporated 13 trials; one of them quasi-randomized. Effects were small to moderate on pain, symptom severity, quality of life, depression, and anxiety, favoring the mindfulness-based therapies. However, this meta-analytic aggregation needs to be updated due to the growing body of literature in this field in recent years. Whereas the research base for “third wave” therapies in fibromyalgia has been recently meta-analytically examined (10) based on 8 RCTs and one quasi-randomized controlled trial. Again, the effects were small to moderate, e.g., for pain, health-related quality of life, depression, and anxiety. However, the meta-analysis was limited to a single functional somatic syndrome.

As there is no current quantitative integration of findings for the efficacy of MBCT, MBSR, and ACT for the full scope of bodily distress, the aim of this meta-analysis is to examine the efficacy of these “third wave“ therapies in adults with bodily distress. Hence, we examine the immediate efficacy and maintenance after completion of these treatments in comparison to inactive and non-specific control groups. The primary outcome is somatic symptom severity. However, a comprehensive evaluation should not be limited to somatic symptoms alone (13). Therefore, we also examine the effects on perceived health status, comorbid psychopathologies as well as mechanisms that are considered central to the “third wave” therapies.

## 2. Methods

### 2.1. Protocol and openness

The project is reported according to the PRISMA 2020 statement (14) and was registered on OSF (Registration doi: 10.17605/OSF.IO/G7HBY). All analyses were computed in R version 4.2.1 (15). A complete list of packages used can be found in the supplement. The data (within an R environment) and the full R code are available on OSF (https://osf.io/g7hby).

The investigators of the primary trials report positive ethics votes for these studies, and there are no ethical requirements that this meta-analysis would violate.

### 2.2. Eligibility criteria

Study participants needed to be adults (≥18 years old) and fulfill the criteria for bodily distress (a complete list of included diagnoses can be found in the Supplementary material). Although there is a considerable overlap between hypochondriasis/illness anxiety and bodily distress, studies solely focussing on these diagnoses will be excluded as well as studies on body dysmorphic disorder. The assessment of bodily distress should either be based on a diagnostic interview, or scores above a certain threshold on a validated symptom scale. Eligible therapies were MBCT, MBSR, and ACT, while studies examining conventional CBT were not included. Any of these “third wave“ therapies had to be compared to an inactive control arm or unspecific treatment within the study, i.e., no treatment, wait-list (WL), treatment-as-usual (TAU), enhanced care (EC, e.g., TAU with additional psychoeducation or counseling) or attention/psychological/pill placebo. Furthermore, only RCTs and (results of the first part of) cross-over trials were selected if they entailed *n* ≥ 10 participants per arm (16). Additionally, reports had to be published in English or German.

### 2.3. Search strategies and study selection

PubMed, PsycInfo, Psyndex, Cochrane Central Register of Controlled Trials (CENTRAL), and Cochrane Database for Systematic Reviews (CDSR) were last searched on 01st of October 2021. The complete search algorithms can be found in the Supplementary Material.

For study selection, abstracts were scanned for eligibility after the removal of duplicates. Subsets of the reports were each selected by two independent researchers and disagreements were resolved by discussion. Afterwards, the full articles of the reports that had not been excluded in the screening process were assessed for eligibility. During an additional backwards search, the references of meta-analyses and systematic reviews found during the first phase were searched for further eligible reports. None of the independent researchers (FMgB and LH) were blinded to any aspects of the studies at any time during the process.

### 2.4. Data extraction

For each report, two researchers (FMgB and LH; FMgB and a research assistant for the studies identified in the final search update) extracted data independently using a previously prepared Microsoft Excel-sheet (17) (see Supplementary material for a complete list of extracted variables). If reported, results of intent-to-treat (ITT)-analyses were extracted. Otherwise, per-protocol (PP) data were assessed. If multiple measures were used for the assessment of similar outcomes, validated measures were preferred. If multiple or none of the measures were validated, FMgB, LH, and a research assistant decided which measure was more suitable. AM supervised these decisions while being blinded to the results. The decision-making process was documented (see Supplementary material). If multiple publications were based on one dataset, the publications were considered as a single study. If data could not be extracted from the published paper (or Supplementary material), e.g., if it was not possible to calculate effect sizes, the original authors were contacted and asked to provide the relevant data.

### 2.5. Risk of bias assessment

The risk of bias (RoB) was assessed with the revised Cochrane risk of bias tool for randomized trials (RoB 2.0) (18) by two independent researchers (in the same constellations mentioned above).

### 2.6. Outcome measures

Outcomes were examined for post-treatment assessment as well as for short-term (up to 3 months) and long-term follow-up assessments (>3 months) (19). If multiple follow-up assessments were provided all of which fell into either the short-term or the long-term category, the longest of these follow-up periods was included.

The primary outcome was somatic symptom severity. Secondary outcomes were perceived health status (PHS), depressive symptoms, anxiety symptoms, and health anxiety; PHS included quality of life, and health-related quality of life measures as well as impairment due to illness (20). Additionally, some exploratory analyses were performed for relevant constructs in the included treatments: mindfulness, psychological inflexibility, and pain acceptance. On the one hand, mindfulness is considered a core mechanism of MBCT as well as MBSR and is also used in ACT. On the other hand, tackling psychological inflexibility is the main focus in ACT (7). To achieve an increased psychological flexibility, ACT aims at pain acceptance, i.e., acceptance of symptoms, in patients with bodily distress to reduce avoidance behaviors (4).

### 2.7. Data analyses

Since different scales were used to assess the outcomes of interest throughout the included studies, standardized mean difference (SMD) was chosen as summary statistic for all outcomes. More precisely, Hedges' *g* was calculated. Especially, as the number of participants was expected to be small in some groups, Hedges' *g* holds the benefit that it is adjusted for small sample bias (21). If no total score but scores from several subscales were available for an outcome, we combined the subscales into one total score using a method for integrating dependent effect sizes (22), if applicable (see Supplementary material for formulas). The values extracted for PHS scales on which higher scores indicate higher impairment were multiplied by (−1) to ensure all scales pointed in the same direction (23). Consequently, for PHS and mindfulness positive effects indicate an improvement favoring the treatment condition. For all other outcomes negative effects indicate a reduction of symptoms favoring the treatment condition.

A random effects model was used in this analysis as the aim of this analysis is to draw statistical inferences on the underlying universe based on the study results (24). The recommendation to use the restricted maximum likelihood (REML) estimator for τ^2^ for continuous data was followed (25). The *I*^2^-statistic was calculated to quantify heterogeneity (23).

### 2.8. Moderator analyses

Several subgroup analyses were conducted using a mixed-effects model, presuming each subgroup containing an individual true overall effect and random effects within subgroups (26). In order to compute the analyses with dichotomized categories, we dummy coded the groups. We only calculated subgroup analyses for subgroups with *k* ≥ 3. For the meta-regressions we used a mixed-effects model as well.

First, despite their parallels, the different therapies differ in their foci which could influence outcome. Thus, we examined whether the mindfulness-based (MBCT/MBSR) and the acceptance-based (ACT) approaches differ in their efficacy. Additionally, we planned to compare therapies that were delivered in a face-to-face setting to internet-based interventions. Since the choice of control group typically influences the effect size (27), another subgroup analysis was computed to investigate the impact of control group conditions. We dichotomized the control groups into non-specific and inactive control groups. While we considered WL inactive comparisons, we classified psychological or attention placebos, TAU, and enhanced care (e.g., TAU combined with regular counseling or psychoeducation) as non-specific control groups. Besides, we conducted subgroup analyses for the different diagnoses (i.e., disorders) included as recommended by Lakhan and Shofield (12). Additionally, we examined the influence of treatment intensity as low-intensity treatments usually result in smaller effects [e.g., (2)]. Hence, a subgroup analysis was performed comparing low- and high-intensity psychotherapy (28), i.e., manuals with less than eight sessions vs. manuals with eight or more sessions. In line with this, we performed an explorative meta-regression on number of sessions. Another meta-regression was conducted on therapy dosis, i.e., total time of therapy sessions in minutes. We deviated from the registered moderator analyses: We dichotomized some moderators to be able to form larger subgroups. Further, no analyses on type of assessment were conducted. Lastly, we were not able to conduct useful analyses on baseline scores, neither on somatic symptom severity nor on comorbidities (see Supplementary material for details on these deviations from registered protocol).

### 2.9. Sensitivity analyses

A sensitivity analysis was performed to evaluate the use of the random effects model, comparing results in the meta-analyses using fixed effect and random effects models. In addition, based on outlier analyses, we computed influence analyses (leave one out-method) as well as Graphic Displays of Heterogeneity (GOSH plots) to display study heterogeneity graphically (29). Lastly, another sensitivity analysis was conducted to examine whether the selection of scales in PHS due to our prioritization influenced results.

### 2.10. Publication bias

To examine possible publication bias, contour-enhanced funnel plots were created and examined for asymmetry for every outcome at post-treatment. Moreover, we computed Egger's regression test to statistically assess asymmetry (30). Furthermore, we used the *p*-curve method, focussing on the distribution of statistically significant *p*-values (31).

## 3. Results

### 3.1. Study selection

**Figure 1:**
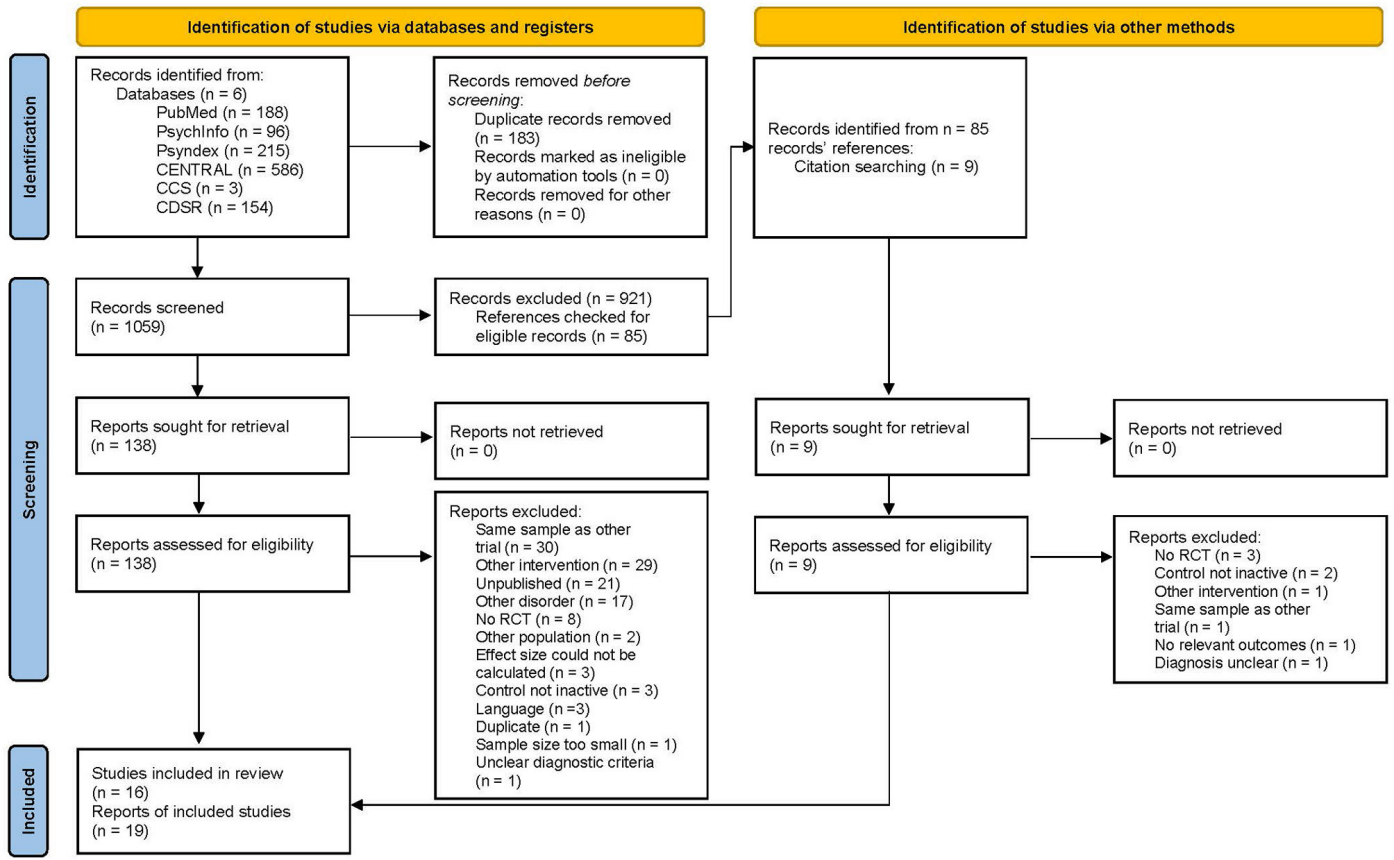
PRISMA 2020 flow diagram [adapted from Page et al. (14)].

For study selection 1,242 reports were retrieved from the databases. After deduplication 1,059 reports were screened. During screening, 921 reports were excluded. Therefore, 138 full-texts were retrieved and assessed for eligibility; 119 of which were excluded after full-text assessment, e.g., 30 reports were excluded as they were based on the same sample as another and did not provide additional relevant data for this project. Finally, we included *k* = 16 studies (with information gathered from 19 reports) including *n* = 1,288 participants randomized (see Figure 1). We gathered additional data from Weissbecker et al. (32), and Cash et al. (33) for the study by Sephton et al. (34), and from Wicksell et al. (35) for Jensen et al. (36). Interrater agreement for study selection was κ = 0.85, i.e., almost perfect (37).

### 3.2. Study characteristics

All included studies focused on adults, one of which explicitly focused on a student population (38). While five studies solely examined women, participants were predominantly female in all other studies. Of the included studies, seven focused on fibromyalgia (34, 36, 39–43), five on irritable bowel syndrome (38, 44–47), one on chronic fatigue syndrome (48), two on bodily distress syndrome (49, 50), and one on medically unexplained symptoms (51). Four of the included studies implemented MBCT, seven studies MBSR, and five studies ACT. Only two of the included studies combined either MBCT (51) or MBSR (50) with methods from conventional CBT. Most studies implemented the therapies in a group setting; only one study (43) was internet-based and therefore patients participated in an individual setting; another study (38) consisted of a self-help approach following a group workshop. One study (49) consisted of more than two potentially relevant arms: extended ACT, brief ACT, and EC. We decided to include the extended ACT arm as well as the EC condition and excluded the brief ACT approach from the analyses. Control groups involved WL (*k* = 7), TAU (*k* = 5), EC (*k* = 3), and psychological placebo (a social support group, *k* = 1). TAU conditions were defined as continuation of current treatment (40, 43, 47), continued medication (41, 45). All studies but one (45) that was set in Iran took place in OECD countries. Further study characteristics are provided in the supplement. For data extraction all disagreements in the independently extracted data were resolved through discussion in a consensus meeting.

### 3.3. Risk of bias

Overall RoB was high across all studies; details on the ratings of individual studies are provided in the supplement. Three domains were predominantly responsible for the high ratings: In the domain “RoB in measurement of the outcome”, we rated the risk of bias as high for all studies except for one. As blinding is rather difficult in psychotherapy trials, and almost impossible in the included designs (e.g., WL vs. psychotherapy), we assumed that the assessors for self-report measures (i.e., the participants themselves) were probably aware of the therapy they received. Only in Gaylord et al. (44) we rated RoB as low as participants perceived the support group as credible as the intervention group. The evaluation of “RoB in selection of reported results” required the availability of a study protocol. Although nine studies provided a protocol, these did not entail information on the planned analyses. In accordance with the RoB tool 2.0, for the domain “RoB due to missing outcome data”, we rated all intention-to-treat methods as adequate that allowed inclusion of all participants for whom data were available.

Due to the initially low interrater reliability (κ = 0.59 for the five domains; κ = 1 for overall RoB judgement) between FMgB and LH, an additional research assistant independently rated all studies with disagreement between the raters and studies identified in the final search update. This improved interrater reliability for these studies (κ = 0.76; κ = 1 for overall RoB judgement).

### 3.4. Treatment effects—post-treatment

**Figure 2:**
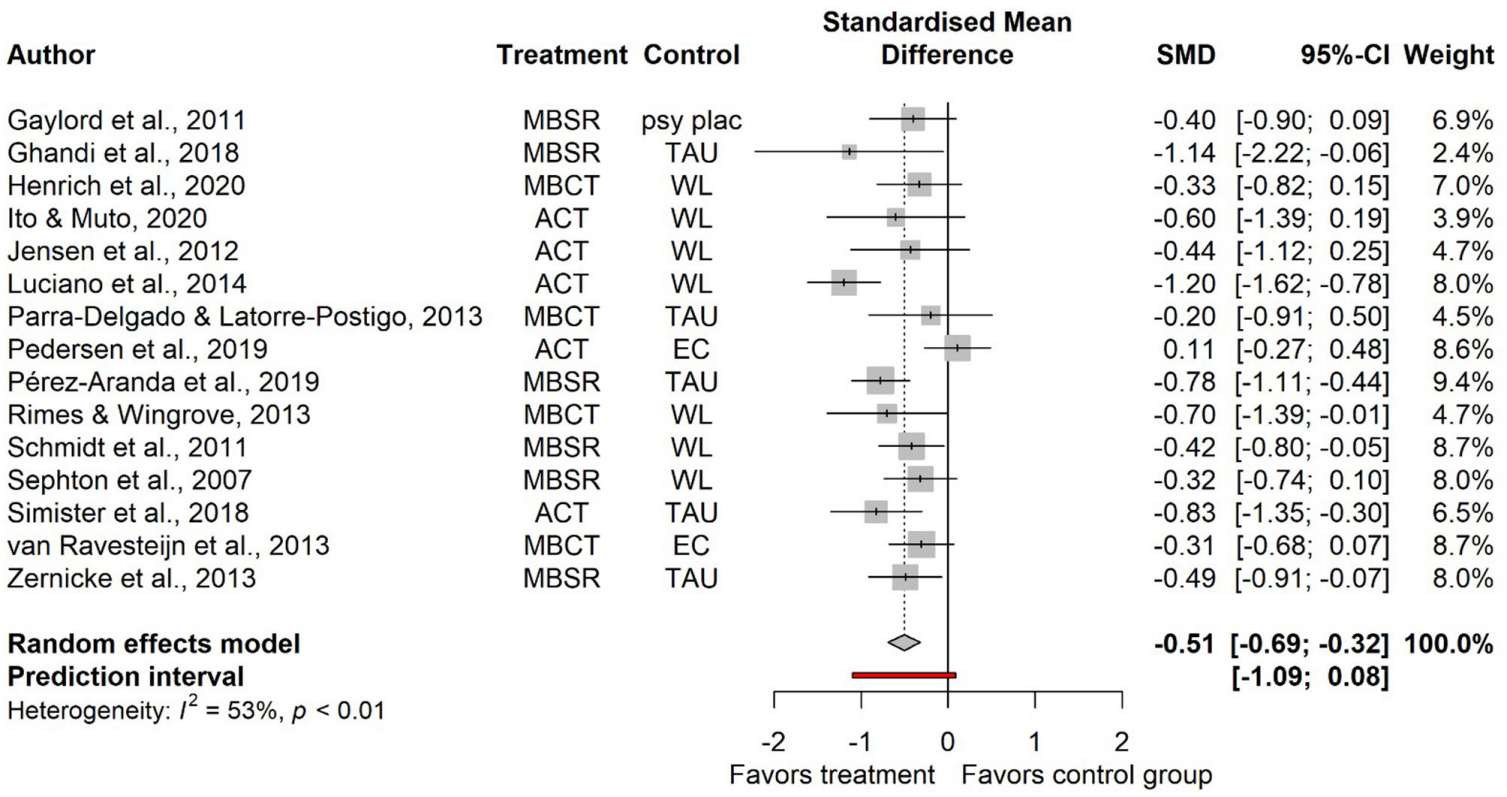
Forest plot for somatic symptom severity (post-treatment).

For the primary outcome, somatic symptom severity, 15 studies provided data at post-treatment (*n* = 1,100). Results showed a significant effect in favor of the “third wave“ therapies compared to inactive and non-specific control conditions (*g* = −0.51, 95%CI −0.69 to −0.32; see Figure 2 for a forest plot and Table 1 for more detailed results on all outcomes). Heterogeneity was 52.8% (95%CI 15.1 to 73.8). All studies reported a measure of PHS (*n* = 1,221). The aggregated effect size was *g* = 0.41 (95%CI 0.11 to 0.72; forest plots for all secondary outcomes are provided in the supplement). Heterogeneity was considerable (83.7%, 95%CI 74.8 to 89.4). For the comorbid psychopathologies the symptoms scores were also reduced in the treatment groups compared to the control groups at post-treatment. Results on depression of 13 studies, based on *n* = 980 participants, were aggregated to an effect of *g* = −0.52 (95%CI −0.82 to −0.23). Anxiety scores were provided in nine studies (*n* = 687) at post–treatment, leading to an effect of *g* = −0.38 (95%CI −0.65 to −0.11). For both comorbid psychopathologies, heterogeneity was substantial (depression: 79.8%, anxiety: 67.4%). The outcomes measures focussing on mechanisms in the “third wave” therapies, i.e., mindfulness (*k* = 8, *n* = 603, *g* = 0.49, 95%CI 0.33 to 0.65), and psychological inflexibility (*k* = 3, *n* = 216, *g* = −0.47, 95%CI, −0.93 to −0.02) improved significantly. While the mean heterogeneity indicated that it might not be of importance or only moderate, the respective 95%CIs were very wide (see Table 1). For health anxiety and pain acceptance, the effects were not aggregated as only two studies reported measures of each construct.

**Table 1:** Treatment effects.

**Outcome**	***k***	***n***	***g***	**95%CI**	***p***	***I*** **^2^**	**95%CI**	**95%PI**
**Post-treatment**								
Somatic Symptom Severity	15	1,100	−0.51	−0.69; −0.32	< 0.001	52.8%	15.1; 73.8	−1.09; 0.08
PHS	16	1,221	0.41	0.11; 0.72	0.008	83.7%	74.8; 89.4	−0.85; 1.67
Depression	13	980	−0.52	−0.82; −0.23	< 0.001	79.8%	66.2; 87.9	−1.61; 0.57
Anxiety	9	687	−0.38	−0.65; −0.11	0.006	67.4%	34.2; 83.8	−1.20; 0.44
Mindfulness	8	603	0.49	0.33; 0.65	< 0.001	0.0%	0.0; 67.6	0.29; 0.69
Psychological inflexibility	3	216	−0.47	−0.93; −0.02	0.043	49.7%	0.0; 85.4	−5.16; 4.21
**Short-term follow-up**								
Somatic Symptom Severity	9	500	−0.45	−0.72; −0.19	< 0.001	53.7%	1.8; 78.2	−1.16; 0.26
PHS	10	554	0.40	0.09; 0.7	0.012	65.1%	31.6; 82.2	−0.60; 1.40
Depression	9	508	−0.40	−0.59; −0.21	< 0.001	26.7%	0.0; 65.8	−0.72; −0.09
Anxiety	6	326	−0.27	−0.49; −0.05	0.017	0.0%	0.0; 74.6	−0.58; 0.04
Mindfulness	6	333	0.32	0.10; 0.54	0.004	0.0%	0.0; 74.6	0.01; 0.63
**Long-term follow-up**								
Somatic Symptom Severity	6	591	−0.40	−0.79; −0.002	0.049	82.3%	62.4; 91.6	−1.74; 0.95
PHS	6	635	0.44	−0.19; 1.08	0.171	91.9%	85.2; 95.6	−1.87; 2.75
Depression	4	453	−0.47	−1.08; 0.14	0.133	88.6%	73.4; 95.1	−3.35; 2.4
Anxiety	3	346	−0.62	−1.12; −0.11	0.016	79.2%	33.7; 93.5	−6.65; 5.42
Mindfulness	3	309	0.37	−0.06; 0.81	0.095	66.4%	0.0; 90.3	−4.62; 5.37

95%CI = 95% confidence interval; 95%PI = 95% prediction interval; *g* = Hedges' *g*; *k* = number of studies; *n* = number of participants; PHS = perceived health status.

### 3.5. Treatment effects—short-term follow-up

For the short-term follow-up period (up to 3 months post treatment), somatic symptom severity was significantly reduced compared to the control conditions (*k* = 9, *n* = 500). The “third wave“ therapies reduced somatic symptom severity in participants (*g* = −0.45, 95%CI −0.72 to −0.19). PHS was again reported in all studies that entailed a short–term follow–up (*k* = 10, *n* = 554). PHS improved significantly in the therapy arms compared to the control conditions (*g* = 0.40, 95%CI 0.09 to 0.71). The improvements in depression (*k* = 9, *n* = 508, *g* = −0.40, 95%CI −0.59 to −0.21), and anxiety (*k* = 6, *n* = 326, *g* = −0.27, 95%CI −0.49 to −0.05) were maintained at short–term follow–up. The additional outcome mindfulness was reported in *k* = 6 studies (*n* = 333); the aggregated effect was *g* = 0.32 (95%CI 0.10 to 0.54) at short-term follow-up. The estimates on quantified heterogeneity had rather wide 95%CIs for all outcomes (see Table 1). Health anxiety was not reported in any study at short-term follow-up; psychological inflexibility (*k* = 2) and pain acceptance (*k* = 1) were too rarely reported at short-term follow-up to be aggregated.

### 3.6. Treatment effects—long-term follow-up

Only six (*n* = 591) studies reported the primary outcome, somatic symptom severity, at follow-up periods longer than 3 months (mean follow–up period was ~9.57 months, *SD* = 3.55). Nonetheless, for these studies the aggregated effect was significant (*g* = −0.40, 95%CI −0.79 to −0.002). However, we could not find a significant effect at long–term follow–up for PHS (*k* = 6, *n* = 635; *g* = 0.44, 95%CI −0.19 to 1.08). While we found a significant effect for anxiety (*k* = 3, *n* = 346, *g* = −0.62, 95%CI −1.12 to −0.11), the effect on depression (*k* = 4, *n* = 453, *g* = −0.47, 95%CI −1.08 to 0.14) was not significant at long–term follow–up anymore. For the exploratory outcome mindfulness, only three studies (*n* = 309) reported results at follow–ups longer than 3 months after end of treatment. These findings did not result in a significant aggregated effect (*g* = 0.37, 95%CI −0.06 to 0.81). For all outcomes, heterogeneity was substantial to considerable. Only two studies reported health anxiety and only one study each reported psychological inflexibility and pain acceptance at follow-up. Hence, effects were not aggregated for these outcomes.

### 3.7. Moderator analyses

When comparing acceptance-based therapies (ACT) with the mindfulness-based therapies (MBCT and MBSR) we did not find a significant group difference for somatic symptom severity (*p*_*subgroup*_ = 0.658). For the acceptance–based therapies (*k* = 5) the effect was *g* = −0.59 (95%CI −1.07 to −0.10) and for mindfulness–based therapies (*k* = 10) it was *g* = −0.47 (95%CI −0.62 to −0.33). However, heterogeneity was larger in acceptance–based therapies (*I*^2^ = 81.8%) than in mindfulness–based therapies (*I*^2^ = 0.00%). We did not find a significant effect of *type of treatment* in any other outcome, either (more detailed results on all moderator analyses can be found in the Supplementary material). As we only identified one study that implemented an internet-based intervention, we did not perform a subgroup analysis for *modus of treatment* (face-to-face vs. internet-based). When dichotomising control groups into *inactive and non-specific control conditions*, we did not find significant subgroup differences for any outcome, either. Regarding subgroup analyses for the different *diagnoses* (i.e., disorders) included, we could only examine differences between the subgroups of irritable bowel syndrome and fibromyalgia as all other subgroups did not hold enough studies (*k* < 3). Here, we did not find significant subgroup differences for any outcome. *Therapy dosis* (i.e., total duration of therapy sessions) was not a significant moderator for any outcome, neither was the *number of sessions*. We did not find significant subgroup differences between *low- and high-intensity treatments* for any outcome. We did not examine moderators for follow-up periods as we only found little studies for these intervals.

### 3.8. Sensitivity analyses

The sensitivity analysis comparing use of the random effects model to the *fixed effect model* did not reveal major differences. As expected, the 95%CIs were larger in the meta-analyses based on the random effects model. The sensitivity analyses concerning the *choice of PHS scales* did not result in meaningful differences. In the *outlier analyses*, we identified two outliers for somatic symptom severity (39, 49), as well as for PHS (39, 51), and one outlier for depression (39) at post-treatment. After excluding the outliers, the effect sizes decreased for all three outcomes. That is, the lower end of the 95%CIs of all outliers was above the upper end of the 95%CI for the respective pooled effect. Furthermore, the mean *I*^2^ values decreased when the outliers were excluded from the analyses. However, the 95%CIs for the heterogeneity in the analyses with outliers and without outliers still overlapped. We did not identify any outliers for anxiety or mindfulness at post-treatment. Details on the sensitivity analyses can be found in the Supplementary material.

### 3.9. Publication Bias

**Figure 3:**
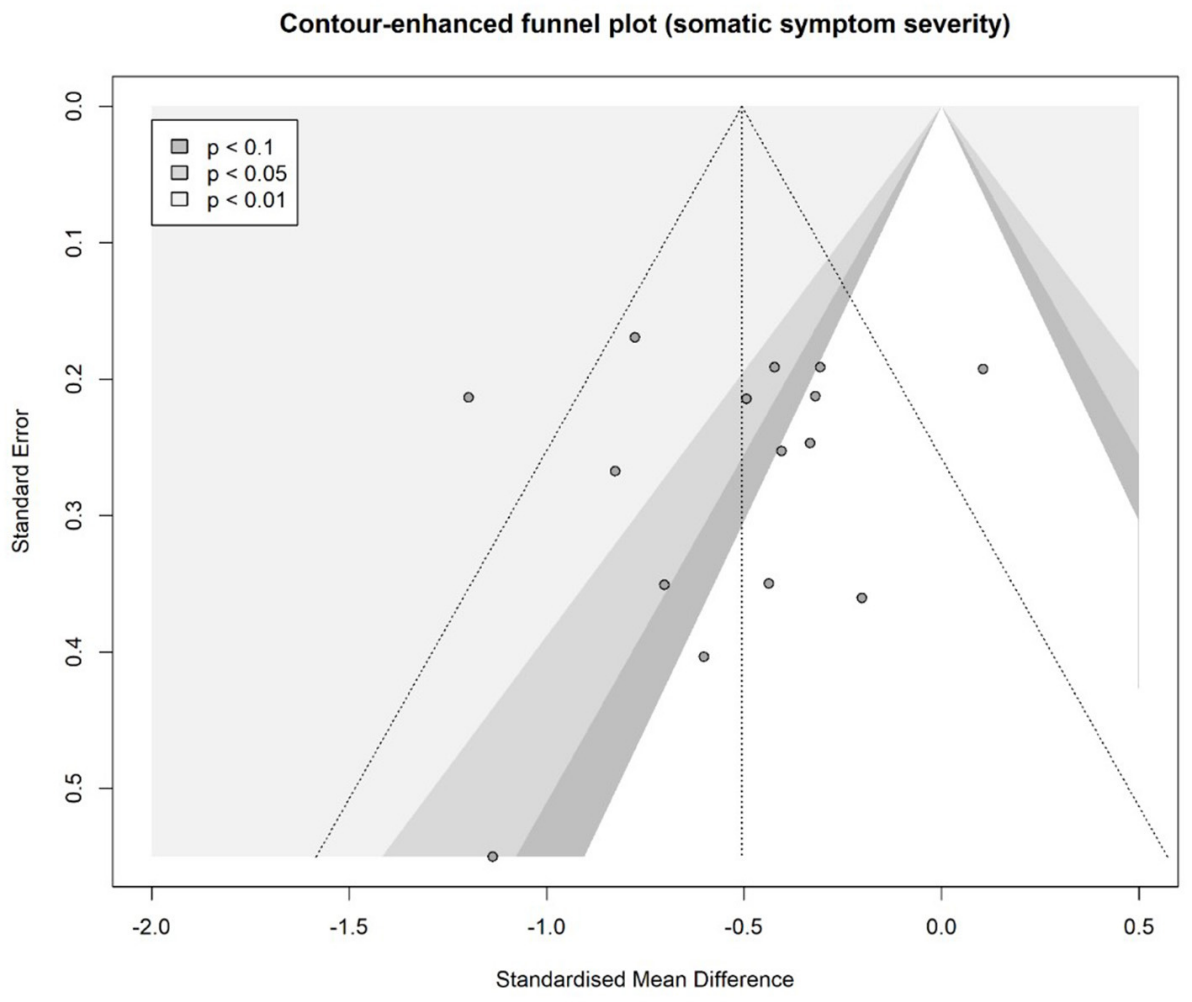
Contour-enhanced funnel plot for somatic symptom severity (post-treatment).

Eggers' test did not indicate the presence of funnel plot asymmetry for any outcome at post-treatment (see Figure 3 for the contour-enhanced funnel plot for somatic symptom severity at post-treatment; details and funnel plots for all other outcomes are provided in the supplement). However, for anxiety as well as for mindfulness, power might not have been adequate to detect a potential bias. Furthermore, for health anxiety, psychological inflexibility, and pain acceptance we did not analyse publication bias due to the small number of studies. In the *p*-curve analyses, we found that evidential value was present for all outcomes at post-treatment as far as it was possible to determine (*p*-curves are depicted in the supplement). However, it was not possible to examine anxiety or psychological inflexibility due to sample size restrictions.

## 4. Discussion

For adults with bodily distress, the effects of the included “third wave” therapies (ACT, MBCT, and MBSR) at post-treatment indicate a reduction in somatic symptom severity, and of comorbid symptoms of depression and anxiety. Further, results suggest an improvement related to PHS, reflected by an increased (health-related) quality of life and a reduction of impairments due to illness. The effects at short-term follow-up (up to 3 months) suggest maintenance of the effects in somatic symptom severity. The effects on PHS, depression, and anxiety were maintained as well. However, the effects at long-term follow-up on somatic symptom severity and anxiety were small; the effects on PHS and depression were insignificant.

At post-treatment and short-term follow-up, we found small to medium effect sizes. On the long run, effects on somatic symptom severity and anxiety were small. That is, the mindfulness- and acceptance based therapies are more effective than inactive and non-specific control conditions. However, there was a significant amount of heterogeneity. While one might deem the broad definition in PHS accountable for the amount of the substantial heterogeneity in the effects on this measure, we want to point out that we also found a large amount of heterogeneity in depression. The latter was straightforwardly operationalised in the included studies by using established self-report depression scales for assessment.

All in all, the effects are in line with previous research. In comparison to former meta-analyses in the field, the data base for this meta-analysis was considerably improved. A certain overlap (*k* = 6) with Lakhan and Schofield's (12) meta-analysis was given. That is, half of their included trials were incorporated in our analyses, too. Six of the nine total studies included in the latest meta-analysis on “third wave” therapies in fibromyalgia (10) were also identified in the present meta-analysis. However, these former projects included not only a quasi-randomized controlled trial (52), but also trials that did not solely rely on established “third wave” therapy manuals. Findings here included short-term maintenance of treatment effects and, in some outcomes, over the longer follow-up. In a previous work (12) the reported outcomes were descriptively smaller. Still, the 95%CIs overlapped. The overlap of confidence intervals does not necessarily imply that the results are comparable, however, it can be an indicator that they are. In Haugmark et al. (10), the effect on health-related quality of life was larger than our effect on PHS which might be attributed to differences in operationalisation decisions. However, the 95%CIs overlapped here as well. Lakhan and Schofield (12) did not aggregate follow-up effects meta-analytically.

Although the effects are only small to moderate, ACT, MBCT, and MBSR may be clinically useful, as the main goal in psychotherapy approaches for bodily distress is not a full remission, but to enable patients to adaptively cope with their symptoms (53). While the “third wave“ therapies and conventional CBT were not directly compared, the effect sizes we found are comparable to those of conventional CBT (1). This means that mindfulness- and acceptance-based therapies could possibly pose an effective treatment alternative for patients suffering from bodily distress.

While some patients might benefit more from these “third wave” therapies than from CBT, specific predictors still have to be identified. Hence, we included measures of potential change mechanisms as exploratory outcomes. Mindfulness, central to MBCT and MBSR, improved significantly at post-treatment, and this effect was maintained at short-term follow-up. However, at long-term follow-up the effect was no longer significant. Psychological inflexibility was lower at post-treatment in the groups that received “third wave” therapies, indicating that ACT in fact tackles psychological inflexibility. Overall, the number of follow-up studies on mechanism variables is very small, and no data could be integrated for psychological inflexibility or pain acceptance due to lack of studies. It also remains unclear whether these effects are associated with the changes in clinical outcomes, as it was not possible to examine this relationship in this meta-analysis.

The moderator analyses did not identify relevant sources of heterogeneity. That is, e.g., the effect on somatic symptom severity seems to be independent of the specific type of treatment and treatment intensity or dosis. However, subgroup and moderator analyses usually lack power (54). Consequently, non-significant results do not necessarily indicate that there is no effect. Generally, for the outcomes that were seldomly reported, the 95%CIs were larger due to limited accuracy. As the number of studies decreased for the follow-up periods, the confidence intervals grew larger for the other outcomes, too. Same goes for the accuracy of the heterogeneity measure. Therefore, we advise researchers to comply to the core domains of bodily distress recommended by the EURONET-SOMA group (13), as conclusions could not be drawn for all of them.

Furthermore, almost all studies evaluated treatments in a group setting, including the studies on ACT—which is not a group program *per se*. Thus, the efficacy of ACT, MBCT, and MBSR as individual therapy cannot be assessed (we also did not find evidence that the study implementing ACT in an individual (internet-based) setting (43) had a substantial impact on the results). Individual therapy may have the advantage of being better tailored to the individual problem and treatment goal (4). However, treatments with group format could have the advantage of being more cost-efficient. Nonetheless, except for the social support group condition in one trial (44), all control groups in the included studies were individual-focused rather than group therapy-focused. Therefore, we cannot determine whether the effects of the included “third wave” therapies may have been influenced by the group component. It is possible, for example, that certain desirable effects result from the social support in groups alone (55).

A factor limiting generalization of results to the wide scope of bodily distress is that most included studies were on functional somatic syndromes and only three on other conditions within the category of somatoform disorders. It is noteworthy that so far there is no study that investigated the efficacy of “third wave” therapies in Somatic Symptom Disorder or bodily distress disorder, i.e., recently introduced classification approaches predominantly focussing on the excessive psychobehavioral components of bodily distress. Mindfulness-based or acceptance and commitment therapy might be especially useful for these disorders as they could help in reducing excessive psychobehavioral symptoms such as excessive symptom related preoccupation and worries as well as experiential avoidance.

The results need to be interpreted in the light of potential biases. RoB ratings were rather high for the included studies. Some domains lead to high RoB ratings when studies were designed according to conventions in psychotherapy trials in bodily distress. For example, all outcome scores relied on self-reports increasing RoB scores in the domain “RoB in measurement of the outcome”. That is, we considered the patients as outcome assessors which were probably not blinded due to a lack of convincing (placebo-)control groups (56). In the “RoB in the selection of reported results” most studies were rated high for the majority of outcomes. However, selective reporting of analyses could not be examined in most registrations (e.g., on clinicaltrials.gov) as the templates lack options to provide information on analysis plans.

Our analysis had several strengths. We complied with current guidelines on conducting, analyzing, and reporting meta-analytic results. Based on a preregistered rational, we examined efficacy on various manifestations of bodily distress. Furthermore, we integrated follow-up effects of the included “third wave” therapies, for which aggregated data for the entire field of bodily distress was previously lacking [e.g., (12)].

However, there were some conceptual limitations to this study. First and foremost, the definition for “third wave” therapies that was used in this study solely entailed MBCT, MBSR, and ACT. Subsequently, this project allows statements regarding the efficacy of these three therapies, which have now been the subject of a number of studies in bodily distress. However, an assessment regarding the efficacy of other “third wave” approaches (like meta-cognitive therapy) is currently not possible, yet. Apart from that, only inactive and non-specific control groups were taken into account which could result in an overestimation of the effect sizes (27). No conclusions can be drawn regarding the efficacy of “third wave” therapies in comparison to other active treatments, especially evidence-based treatments.

Lastly, we did not include gray literature, i.e., unpublished reports. Nonetheless, there was no indication of publication bias.

### 4.1. Prospects

Future updates of this meta-analysis would be a useful tool to include new findings in the field of psychotherapy in adults with bodily distress; ideally incorporating all core outcome domains in bodily distress as well as more long-term follow-ups. Moreover, it might be useful to collect individual patient data, i.e., primary data from RCTs, and combine them into one dataset to enable researchers to identify mediators and moderators of effects in “third wave” therapies. Nonetheless, larger trials are needed, preferably RCTs that help to identify mechanisms in symptom reduction. Future RCTs should also implement control groups that control for unspecific effects in the intervention examined, e.g., peer-support as a group component in group therapy. Considering the distribution of diagnoses within our sample, more studies on Somatic Symptom Disorder and bodily distress disorder would be especially beneficial to the field.

### 4.2. Conclusions and implications

The results of this meta-analysis suggest that ACT, MBCT, and MBSR can be beneficial treatment approaches for bodily distress in adults, showing improvements in somatic symptom severity, comorbid psychopathology, and perceived health status. This meta-analysis provides a good summary of the current state of research of the included “third wave” therapies' efficacy in bodily distress using an enlarged data base due to the focus on bodily distress and not solely focussing on a single syndrome or symptom. All in all, these results could form the basis for these “third wave” therapies to be included in treatment guidelines, while acknowledging that more research is needed to reliably draw conclusions on the efficacy in all relevant domains of bodily distress—especially in the long run.

## Data availability statement

The original contributions presented in the study are included in the article/Supplementary material, further inquiries can be directed to the corresponding author.

## Author contributions

FMgB and AM developed the research question and conceptual background for this work and prepared the final manuscript for publication. FMgB and LH developed the outline, formulated the search terms, and independently performed the literature search, data extraction, and risk of bias assessment (with the help of a research assistant). FMgB performed data analyses and provided feedback on the manuscript. AM regularly provided feedback on the progress and results. All authors contributed to the article and approved the submitted version.
