# Supplementary material for: Efficacy of mindfulness- and acceptance-based cognitive-behavioral therapies for bodily distress in adults: a meta-analysis
Source: Front Psychiatry. 2023 Apr 20;14:1160908. doi: 10.3389/fpsyt.2023.1160908 (PMC10157071; doi:10.3389/fpsyt.2023.1160908)
Supplement: Supplementary file 1 [file Presentation_1.pdf]

Supplementary Material

Efficacy of mindfulness- and acceptance-based cognitive-behavioral therapies for bodily distress in adults: a meta-analysis

Frederic Maas genannt Bermpohl*, Lea Hülsmann, Alexandra Martin

*** Correspondence:** Frederic Maas genannt Bermpohl: fmaas@uni-wuppertal.de

1 Eligible syndromes and diagnoses

- ICD-10 (1):
  - Somatization disorder (F45.0)
  - Undifferentiated somatoform disorder (F45.1)
  - Dissociative (conversion) disorders (F44.)
  - Persistent somatoform pain disorder (F45.4)
  - Somatoform autonomic dysfunction (F45.3)
  - Other somatoform disorders (F45.8)
  - Somatoform disorder unspecified (F45.9)
  - Neurasthenia (in other neurotic disorders category; F.48.0)
- ICD-11 (2): Bodily Distress Disorder (6C40)
- DSM-IV (3):
  - Somatization Disorder (300.81) (4)
  - Undifferentiated somatoform disorder (300.81)
  - Conversion disorder (300.11)
  - Pain disorder (307.80)
  - Somatoform disorder not otherwise specified (300.81)
- DSM-5 (5): Somatic Symptom Disorder (300.82) (6)
- Bodily Distress Syndrome (7)
- Multisomatoform Disorder (8)
- Abridged Somatization (SSI-4/6; somatic symptom index) (9)
- Physical Symptom Disorder (10)
- Polysymptomatic Distress Disorder (4)
- Complex Somatic Symptom Disorder (11)
- Medically unexplained symptoms (MUS, 12)
- Functional Somatic Disorders (13)
- multi-symptom Functional Somatic Syndromes
  - IBS
    - Rome-I to -IV (14)
    - German consensus guidelines (15),
    - ICD-10 (1): K85.
  - CFS and myalgic enzephalomyelitis (ME) will be combined under the term CFS

    - Holmes Criteria (16)
    - Oxford criteria (17)
    - Fukuda definition/Centers for Disease Control and Prevention (CDC) definition (18)
    - Canadian consensus criteria (19)
    - international consensus criteria (20)
    - NICE guidelines (21)
    - ICD-10 (1): Neurasthenia (F48.0), unspecified Chronic fatigue (R53.82), ME/postviral fatigue syndrome (G.93.3)
    - Systemic Exertion Intolerance Disease (22, 23)
- FM/Fibromyalgia syndrome (FMS)
    - American College of Rheumatology (ACR) 1990 Criteria (24), ACR 2010 preliminary diagnostic criteria (25), 2016 Revisions to the 2010/2011 fibromyalgia diagnostic criteria (26)
    - ICD-10 (1): M79.7
    - Chronic Widespread Pain: Defining feature of FM, although not pathognomonic (27)

2 Prioritization of exclusion criteria

Reasons for exclusion were prioritized as follows: focus is not on bodily distress; treatment is not CBT or a third wave treatment; focus is not on human adults (e.g., children, adolescents, animals); the study is not an RCT; neither control group is inactive, CBT or a third wave treatment; the report was not published in English or German.

2.1 Excluded studies

There are several studies we excluded but that might appear to meet our inclusion criteria:

- Amutio et al. (28): no clear definition as for how the diagnosis of FM was assessed
- Andrés-Rodríguez et al. : same sample as Pérez-Aranda et al. (29, subset)
- Givehki et al. (30): they assessed relevant outcomes but did not report them; authors did not answer inquiries
- Mirsharifa et al. (31): no clear definition as for how the diagnosis of IBS was assessed; authors did not answer inquiries
- Mohamadi et al. (32): reporting of relevant outcomes was considered faulty; authors did not answer inquiries
- Zomorrodi et al. (33): only PHS was assessed of the relevant outcomes; its reporting is considered faulty; authors did not answer inquiries
- Ljótsson et al. (34, 35): exposure therapies with additional mindfulness elements
- Kleinstäuber et al. (36) : the intervention mainly contained conventional CBT with additional 3rd Wave methods

3 Search strategies

3.1 Search strategy PubMed

((“Somatoform disorders” [MeSH Terms] OR somatization disorder [MeSH Terms] OR somatization disorder [MeSH Terms] OR Neurasthenia [MeSH Terms] OR “conversion disorder” [MeSH Terms] OR psychophysiological disorder [MeSH Terms] OR “Psychosomatic medicine” [MeSH Terms] OR Fibromyalgia [MeSH Terms] OR “Fatigue Syndrome, Chronic” [MeSH Terms] OR “Irritable Bowel Syndrome” [MeSH Terms] OR “Colonic diseases, Functional” [MeSH Terms] OR briquet syndrome [MeSH Terms] OR

“somatoform disorder*” [All Fields] OR somatoform [All Fields] OR somatiz* [All Fields] OR somatis* [All Fields] OR “somatic symptom disorder*” [All Fields] OR (somatic NEAR symptom*) OR (pain NEXT disorder) OR “psychophysiological disorder*” [All Fields] OR “psychophysiologic disorder*” [All Fields] OR psychosomat* [All Fields] OR “persistent physical symptom*” [All Fields] OR “persistent somatic symptom*” [All Fields] OR “bodily distress” [All Fields] OR "body distress" [All Fields] OR “Physical Symptom Disorder” [All Fields] OR “pain disorder*” [All Fields] OR multisomat* [All Fields] OR "multi?somat*" [All Fields] OR polysymptom* [All Fields] OR "poly?symptom*" [All Fields] OR (multiple AND (“physical symptom*” OR “symptom diagnos*”)) OR

„frequent attend*” [Title/Abstract] OR „high utilis*” [Title/Abstract] OR „high utiliz*” [Title/Abstract]

OR “medically unexplain*” [All Fields] OR “organically unexplain*” [All Fields] OR MUS [All Fields] OR MUPS [All Fields] OR “unexplained medical*” [All Fields] OR "unexplained symptom*" [All Fields] OR "unexplained syndrom*" [All Fields] OR "unexplained illness*" [All Fields] OR nonorganic [All Fields] OR “non?organic” [All Fields] OR “non?specific complain*” [All Fields] OR “non?specific symptom*” [All Fields] OR “psychosomatic syndrom*” [All Fields] OR

FSS [All Fields] OR "functional somatic syndrome*" [All Fields] OR "functional somatic disorder*" [All Fields] OR "functional symptom" [All Fields] OR "functional disorder*" [All Fields] OR "functional syndrome*" [All Fields] OR "functional somatic" [All Fields] OR "functional illness*" [All Fields] OR ((irritable OR functional) AND (bowel* OR colon*)) OR “functional gastrointestinal*” [All Fields] OR IBS [All Fields] OR fibromyalgia* [All Fields] OR fibrositis [All Fields] OR FM [All Fields] OR fibromyositis [All Fields] OR myofibrositis [All Fields] OR “chronic widespread pain*” [All Fields] OR “widespread musculoskeletal pain*” [All Fields] OR “myofascial pain*” [All Fields] OR “persistent pain*” [All Fields] OR “chronic intractable benign pain*” [All Fields] OR “fatigue syndrome*” [All Fields] OR “psychogenic fatigue” [All Fields] OR “systemic exertion intolerance” [All Fields] OR “myalgic encephalomyelitis*” [All Fields] OR “myalgic encephalopath*” [All Fields] OR CFS [All Fields] OR “CFS/ME” [All Fields] OR “postviral fatigue syndrome*” [All Fields] OR “systemic exertion intolerance*” [All Fields]))

AND

(("third wave" AND (therap* OR treatment*)) OR mindful* [All Fields] OR MBT [All Fields] OR MBCT [All Fields] OR MBSR [All Fields] OR (acceptance AND commitment) OR "mindfulness based stress reduction"[All Fields] OR "mindfulness based cognitive therapy"[All Fields] OR "acceptance and commitment therapy"[All Fields])

Filters: Randomized Controlled Trial, Meta-Analysis, Systematic Review

3.2 Search strategy Cochrane Library

ID Search

#1 MeSH descriptor: [Conversion Disorder] explode all trees

#2 MeSH descriptor: [Somatoform Disorder] explode all trees

#3 MeSH descriptor: [Somatoform Disorders] explode all trees

#4 MeSH descriptor: [Neurasthenia] explode all trees

#5 MeSH descriptor: [Psychophysiologic Disorders] explode all trees

#6 MeSH descriptor: [Psychosomatic Medicine] explode all trees

#7 MeSH descriptor: [Fibromyalgia] explode all trees

#8 MeSH descriptor: [Fatigue Syndrome, Chronic] explode all trees

#9 MeSH descriptor: [Irritable Bowel Syndrome] explode all trees

#10 MeSH descriptor: [Colonic Diseases, Functional] explode all trees

#11 “somatoform disorder*” OR somatoform OR somatiz* OR somatis* OR “somatic symptom disorder*” OR (somatic AND symptom*) OR (pain NEXT disorder) OR “psychophysiological disorder*” OR “psychophysiologic disorder*” OR psychosomat* OR “persistent physical symptom*” OR “persistent somatic symptom*” OR “bodily distress” OR "body distress" OR “Physical Symptom Disorder” OR “pain disorder*”

#12 multisomat* OR "multi?somat*" OR polysymptom* OR "poly?symptom*" OR (multiple AND (“physical symptom*” OR “symptom diagnos*”))

#13 “medically unexplain*” OR “organically unexplain*” OR MUS OR MUPS OR “unexplained medical*” OR "unexplained symptom*" OR "unexplained syndrom*" OR "unexplained illness*"

#14 nonorganic OR “non?organic” OR “non?specific complain*” OR “non?specific symptom*”

#15 “psychosomatic syndrom*”

#16 FSS OR "functional somatic syndrome*" OR "functional somatic disorder*" OR "functional symptom" OR "functional disorder*" OR "functional syndrome*" OR "functional somatic" OR "functional illness*"

#17 ((irritable OR functional) AND (bowel* OR colon*)) OR “functional gastrointestinal*” OR IBS

#18 fibromyalgia* OR fibrositis OR FM OR fibromyositis OR myofibrositis

#19 “chronic widespread pain*” OR “widespread musculoskeletal pain*” OR “myofascial pain*” OR “persistent pain*” OR “chronic intractable benign pain*”

#20 “fatigue syndrome*” OR “psychogenic fatigue” OR “systemic exertion intolerance” OR “myalgic encephalomyelitis*” OR “myalgic encephalopath*” OR CFS OR “postviral fatigue syndrome*” OR “systemic exertion intolerance*”

#21 ("third wave" AND (therap*OR treatment*))

#22 mindful* OR MBT OR MBCT OR MBSR

#23 (acceptance AND commitment)

#24 #1 OR #2 OR #3 OR #4 OR #5 OR #6 OR #7 OR #8 OR #9 OR #10 OR #11 OR #12 #13 OR #15 OR #16 OR #17 OR #18 OR #19 OR #20

#25 #21 OR #22 OR #23

#26 #24 AND #25

3.3 Search strategy PsycInfo

(“somatoform disorder*” OR somatoform OR somatiz* OR somatis* OR “somatic symptom disorder*” OR (somatic AND symptom*) OR “psychophysiological disorder*” OR “psychophysiologic disorder*” OR psychosomat* OR “persistent physical symptom*” OR “persistent somatic symptom*” OR “bodily distress” OR "body distress" OR “Physical Symptom Disorder” OR “pain disorder*” OR multisomat* OR "multi?somat*" OR polysymptom* OR "poly?symptom*" OR (multiple AND (“physical symptom*” OR “symptom diagnos*”)) OR „frequent attend*” OR „high utilis*” OR „high utiliz*” OR “medically unexplain*” OR “organically unexplain*” OR MUS OR MUPS OR “unexplained medical*” OR "unexplained symptom*" OR "unexplained syndrom*" OR "unexplained illness*" OR nonorganic OR “non?organic” OR “non?specific complain*” OR “non?specific symptom*” OR “psychosomatic syndrom*” OR FSS OR "functional somatic syndrome*" OR "functional somatic disorder*" OR "functional symptom" OR "functional disorder*" OR "functional syndrome*" OR "functional somatic" OR "functional illness*" OR ((irritable OR functional) AND (bowel* OR colon*)) OR “functional gastrointestinal*” OR IBS OR fibromyalgia* OR fibrositis OR FM OR fibromyositis OR myofibrositis OR “chronic widespread pain*” OR “widespread musculoskeletal pain*” OR “myofascial pain*” OR “persistent pain*” OR “chronic intractable benign pain*” OR “fatigue syndrome*” OR “psychogenic fatigue” OR “systemic exertion intolerance” OR “myalgic encephalomyelitis*” OR “myalgic encephalopath*” OR CFS OR “CFS/ME” OR “postviral fatigue syndrome*” OR “systemic exertion intolerance*”)

AND

(("third wave" AND (therap* OR treatment*)) OR mindful* OR MBT OR MBCT OR MBSR OR (acceptance AND commitment))

AND

("double-blind" OR "random* assigned" OR control)

Methodology: Clinical trial, systematic review, meta-analysis

3.4 Search strategy Psyndex

(„somatisch* Belastung*“ OR „somatisch* Syndrom*“ OR „somatoform*“ OR „somatisier*“ OR „anhaltend* Körperbeschwerde*“ OR „Neurasthenie*“ OR „Konversionsstörung*“ OR „psychophysiologisch*“ OR „psychosomati*“ OR „Briquet Syndrom*“ OR „Schmerzstörung*“ OR (((„nicht spezifisch*“ OR „unspezifisch*“ OR „unklar*“ OR „medizinisch unerklärt*“ OR „organisch unerklärt*“ OR „nicht erklärt*“) AND („Symptom*“ OR „Syndrom*“)) OR „funktionell* Syndrom*“ OR „funktionell* Störung*“ OR „Reizdarm*“ OR „funktionell* gastrointestinal*“ OR „fibromyalgi*“ OR „fibrositis” OR “fibromyositis” OR “myofibrositis” OR (“myofascial*” AND “Schmerz*”) OR “chronisch* Ganzkörperschmerz*” OR („Chronisch*“ OR „psychogen*“) AND („Müdigkeit*“ OR „Erschöpfung*“) OR „myalgic encephalomyelitis*“ OR „myalgic encephalopath*” OR “postviral* Erschöpfung*”)

AND

((„dritte Welle“ AND („Intervention*“ OR „Behandlung*“ OR „Therapie*”)) OR „achtsamkeit*“ OR ((„akzeptanz*“ OR “acceptance”) AND “commitment*”))

4 Study characteristics

Table S1

Study Characteristics

Study	Conditions	*n* allocated	Program Name	Guidance	Diag.	Specific Target Group	Country	No. sessions	Time total[a]	Follow-ups[b] (weeks)
Fjorback et al., 2013	MBSR	60	MBSR + CBT elements	Psychiatrist	BDS	Adults	DK	9	1890	24, 48
	EC	60								
Gaylord et al., 2011	MBSR	36	MBSR	Para	IBS	Women	US	9	1200	12, 24
	Psy. placebo	39								
Ghandi et al., 2018	MBSR	15	MBSR	Psy	IBS	Adults	IR	8	720	8
	TAU	19								
Henrich et al., 2019	MBCT	36	MBCT-IBS	Psy	IBS	Women	GB	6	720	6
	WL	31								
Ito & Muto, 2020	ACT	14	ACT for IBS	Psy	IBS	Students	JP	1	300	8
	WL	12								
Jensen et al., 2012; Wicksell et al., 2013	ACT	25	ACT for chronic pain	Psy	FM	Women	SE	12	1080	12
	WL	18								

Study	Conditions	*n* allocated	Program Name	Guidance	Diag.	Specific Target Group	Country	No. sessions	Time total[a]	Follow-ups[b] (weeks)
Luciano et al., 2014	ACT	51	ACT adapted to FM	Psy	FM	Adults	ES	8	1200	16
	WL	53								
Parra-Delgado & Latorre-Postigo, 2013	MBCT	17	MBCT	Psy	FM	Adults	ES	8	1200	12
	TAU	16								
Pedersen et al., 2019	ACT	59	ACT	Mixed	BDS	Adults	DK	9	1695	32, 56
	EC	60								
Pérez-Aranda et al., 2019	MBSR	75	MBSR adapted for FM	Psy	FM	Adults	ES	8	960	40
	TAU	75								
Rimes & Wingrove, 2013	MBCT	17	MBCT	Psy	CFS/ ME	Adults	GB	9	1215	8
	WL	19								
Schmidt et al., 2011	MBSR	58	MBSR	Para	FM	Women	GER	11	1740	8
	WL	59								
Sephton et al., 2007; Cash et al., 2015; Weissbecker et al., 2002	MBSR	51	MBSR	Psy	FM	Women	US	9	1650	8
	WL	40								
Simister et al., 2018	ACT	33	Online ACT protocol	Psy	FM	Adults	CA	7		12
	TAU	34								

Study	Conditions	*n* allocated	Program Name	Guidance	Diag.	Specific Target Group	Country	No. sessions	Time total[a]	Follow-ups[b] (weeks)
van Ravesteijn et al., 2013	MBCT	61	MBCT + cognitive techniques	Para	MUS	Adults	NL	8	1560	36
	EC	56								
Zernicke et al., 2013	MBSR	43	MBSR	Para	IBS	Adults	CA	8	900	24
	WL	47								

Note. ACT = Acceptance and Commitment Therapy; BDS = bodily distress syndrome; CBT = cognitive behavioural therapy; CFS/ME = chronic fatigue syndrome/myalgic encephalomyelitis; CA = Canada; DK = Denmark; FM = fibromyalgia; EC = enhanced care; ES = Spain; GB = United Kingdom; GER = Germany; IBS = irritable bowel syndrome; IR = Iran; JP = Japan; MBCT = mindfulness-based cognitive therapy; MBSR = mindfulness-based stress reduction; Mixed = guidance provided by psychologist and psychiatrist; NL = Netherlands; Para = paraprofessional (study nurses, trained students); Psy = trained clinician (licensed psychotherapist/trained psychologist); Psy. placebo = psychological placebo; SE = Sweden; TAU = treatment as usual; US = United States of America; WL = wait-list.

[a] total time of direct contact in minutes (was not applicable for Simister et al., 2018); [b] after end of treatment.

Table S2

Outcome measures

Study	Somatic symptom severity	Depression	Anxiety	Health Anxiety	Perceived Health Status	Mindfulness	Psychological Inflexibility	Pain acceptance
Fjorback et al., 2013	SCL-90 (Somatization subscale)[a]	SCL-8[a]	SCL-8[a]	WI [a]	SF-36 (Physical)			
Gaylord et al., 2011	IBS-SSS	BSI-18 Depression	BSI-18 anxiety		IBS-QOL	FFMQ		
Ghandi et al., 2018	IBS-SSS				IBS-QOL-34			
Henrich et al., 2019	GSRS-IBS	DASS-21 (Depression subscale)	DASS-21 (Anxiety subscale)		IBS-QOL	FFMQ		
Ito & Muto, 2020	IBSSI	BDI-II	STAI (State)		SF-36 (Physical); IBS-QOL	FFMQ	CFQ	
Jensen et al., 2012; Wicksell et al., 2013	VAS	BDI	STAI (State)		SF-36 (Physical); FIQ		PIPS	
Luciano et al., 2014	VAS	HADS-D	HADS-A		FIQ; EQ-5D (VAS)			CPAQ
Parra-Delgado & Latorre-Postigo, 2013	VAS	BDI			FIQ			

Study	Somatic symptom severity	Depression	Anxiety	Health Anxiety	Perceived Health Status	Mindfulness	Psychological Inflexibility	Pain acceptance
Pedersen et al., 2019	BDS checklist	SCL-92 (Depression score)	SCL-92 (Anxiety score)	WI-7	SF-36 (Physical); WHO-DAS II			
Pérez-Aranda et al., 2019	FSDC	HADS-D	HADS-A		FIQ-R	FFMQ	PIPS	
Rimes & Wingrove, 2013	CFS	HADS-D	HADS-A		PF-10	FFMQ		
Schmidt et al., 2011	GBB-24	CES-D	STAI (Trait)		PLC; FIQ	FMI		
Sephton et al., 2007; Cash et al., 2015; Weissbecker et al., 2002	VAS	BDI			FIQ			
Simister et al., 2018	SF-MPQ	CES-D			FIQ-R	FFMQ		CPAQ-R
van Ravesteijn et al., 2013	PHQ-15	PHQ-9		WI	SF-36 (Physical); EQ-5D (VAS)	FFMQ		
Zernicke et al., 2013	IBS-SSS				IBS-QOL			

Note. BDI = Beck Depression Inventory; BDI-II = Beck Depression Inventory-II; BDS checklist = Bodily distress syndrome checklist; BSI-18 = Brief Symptom Inventory (short-form); CES-D = Center for Epidemiologic Studies Depression Scale; CFS = Chalder Fatigue Scale; CFQ = Cognitive Fusion Questionnaire; CPAQ = Chronic Pain Acceptance Questionnaire; CPAQ-R = Chronic Pain Acceptance Questionnaire-Revised; DASS-21 = Depressions-Angst-Stress-Skalen [Depression–Anxiety–Stress Scale]; EQ-5D = EuroQol Five-

Dimensional Questionnaire; FFMQ = Five-Facet Mindfulness Questionnaire; FIQ = Fibromyalgia Impact Questionnaire; FIQ-R = Fibromyalgia Impact Questionnaire-Revised; FMI = Freiburg Mindfulness Inventory; FSDC = Fibromyalgia Survey Diagnostic Criteria; GBB-24 = Giessener Beschwerdebogen (Giessen Subjective Complaints List); GSRS-IBS = Gastrointestinal Symptom Rating Scale for IBS; HADS-A = Hospital Anxiety and Depression Scale – Anxiety; HADS-D = Hospital Anxiety and Depression Scale – Depression; IBS-QOL = Irritable Bowel Syndrome Quality of Life survey; IBSSI = Irritable Bowel Syndrome Severity Index; IBS-SSS = Irritable Bowel Syndrome-Symptom Severity Scale; PF-10 = Physical Functioning scale; PHQ-9 = Patient Health Questionnaire-9; PHQ-15 = Patient Health Questionnaire-15; PLC = Quality of Life Profile for the Chronically Ill; PIPS = Psychological Inflexibility in Pain Scale; SCL-8 = eight-item version of the Symptoms Check List; SCL-90 = Symptom Checklist-90; SCL-92 = 92-item version of the Symptom Checklist; SF-36 = Short Form Health Survey; SF-MPQ = McGill Pain Questionnaire-short form; STAI = State-Trait Anxiety Scale; VAS = Visual analogue scale; WI = Whiteley-Index; WI-7 = 7-Item Whiteley-Index; WHO-DAS II = WHO Disability Assessment Schedule 2.0.

[a] data were not extractable or provided by authors.

5 List of extracted data

The following variables, grouped into four categories, were extracted.

Study characteristics:

- Author(s); Year of publication
- Citation (according to APA)
- Protocol: register + registration code/number; link to protocol; name of protocol file (and where to find it)
- Recruitment method
- Incentive provided for completing the treatment/control group & if so, which one
- Number of people assessed for eligibility
- Number of participants wo declined to participate
- Number of participants randomized (for all groups)
- Number of persons allocated that completed at least one module in the treatment or participated in the control group (for all groups, if possible)
- Number of participants completing all modules as advised (for all groups, if possible)
- Post-assessment: duration from baseline to post-treatment assessment (done accordingly for follow-up assessments)

Clinical variables:

- Diagnosis assessment
- Diagnosis assessed
- Specific diagnosis/inclusion criteria regarding symptomology; how was this assessed
- Average and standard deviation (*SD*) for number as well as duration of symptoms (all groups)
- Exclusion of participants with comorbid disorders. If applicable, specify criterion.
- Average and *SD* for severity of somatic symptoms as well as how it was assessed
    - The data for post-treatment and respective follow-up assessments will be extracted.
    - The corresponding means of somatic symptom scores and *SDs* at baseline and post-treatment assessment will be extracted for the treatment group(s) and the control group(s), respectively.
    - Hedges' *g* and the *Standard Error (SE)* will be calculated if the mean and *SD* are not reported, but other measures that allow us to estimate the effect (e.g., 37). This will be done accordingly, for depressive and anxiety symptoms.

Intervention-related variables (for all included interventions)*:*

- Form of treatment(s)
- Manual used. If so, name of the program/manual used
- Form of guidance
- Setting (group vs. individual therapy; internet-/computer-/app-based)
- Form of control group(s)

  - When a TAU-group was given access to the program after the trial was completed – i.e., TAU was combined with WL – it will be considered as TAU. When a group received no treatment, it will be administered as TAU too as it is considered as congruent to what patients received if they did not participate in the study (38).
- Assessment of consumer satisfaction and negative effects assessed and if so, how
- Number of sessions (per week)
- Duration of treatment
- Average proportion of modules completed
- Reasons for dropping out of treatment/control

Sociodemographic variables:

- Target group
- Mean age and *SD* (all groups)
- Proportion of female participants (all groups)
- Country the trial was conducted in

5.1 Combining subscales

We combined multiple subscales to a total score using a method for integrating dependent effect sizes (39, 37). Thus, we averaged means: $d = \frac{dx+dy}{2}$ (respectively $d = \frac{dx+dy+dz+da+db+dc+dd}{7}$ for 40). However, variances were assumed to be highly dependent (41). Rustenbach's (39) approach was applied as this approach additionally integrated the covariance between subscales, resulting in the following formula: $s^2_{(\frac{d_x+d_y}{2})} = \frac{1}{4}(s^2_{dx} + s^2_{dy} + 2 * cov_{d_x d_y})$ (39, p.118f). In order to calculate the covariance, the correlation between the subscales (e.g., r_{xy}) was not reported. Hence, we estimated a correlation of *r* = 0.5 between subscales (see for instance in 42). While this method is not optimal, it is preferred to naïve-methods (e.g. assuming a zero correlation, 41). The calculations were documented in the R Script which is provided online (https://osf.io/an6zy/).

5.2 Prioritization of outcome measures (PHS)

Six studies included more than one measure that we considered relevant for the outcome perceived health status. While we prioritized validated scales, some studies included two validated scales. Here, the decision was made according to the prioritisation of scales, favouring scales that rather assessed a global level of PHS over disorder-specific scales and more elaborate scales: The decision was made in favour of the physical component summary of the SF-36 (vs. FIQ) for Jensen et al. (43) as well as (vs. IBS-QoL) for Ito & Muto (44), (vs. WHO-DAS II) for Pedersen et al. (45), and we decided in favour of the global Scale Quality of Life Profile for the Chronically Ill (vs. FIQ) for Schmidt et al. (46). Furthermore, we decided to prioritize more elaborate scales over single item scales. Hence, we favoured the FIQ (vs. the visual analogue scale, VAS, of the EQ-5D) for van Ravesteijn et al. (47), and (vs. the VAS of the EQ-5D) for Luciano et al. (48).

6 Deviations from preregistration

We did not contact other relevant authors in the field about relevant studies additionally to our strategic literature search. However, we are planning to contact the EURONET-SOMA group regarding relevant studies to create a database on RCTs on all forms of psychotherapies in bodily distress.

Deviations in moderator analyses were as follows: In the protocol we did not specify that we will compare MBCT and MBSR as a combined group against ACT. Furthermore, we chose a different form of dichotomisation for the control groups into inactive and non-specific as wait-list condition is the one eligible control condition possibly leading to the most inflated effect sizes (49). Another deviation from the protocol is that we did not perform a subgroup analysis for the type of assessment as we agreed that it would yield a sufficient benefit in a reassessment in a consensus meeting on the moderator analyses. Lastly, since the effect size (Hedges' *g*) is based on values from both groups, meta-regressions on symptom severity and comorbidity (depression/anxiety) were not possible as the values we extracted were only referring to one of the groups each.

All other deviations are mentioned in the main paper.

7 Risk of bias assessment

Figure S1

Risk of bias ratings for somatic symptom severity at post-treatment

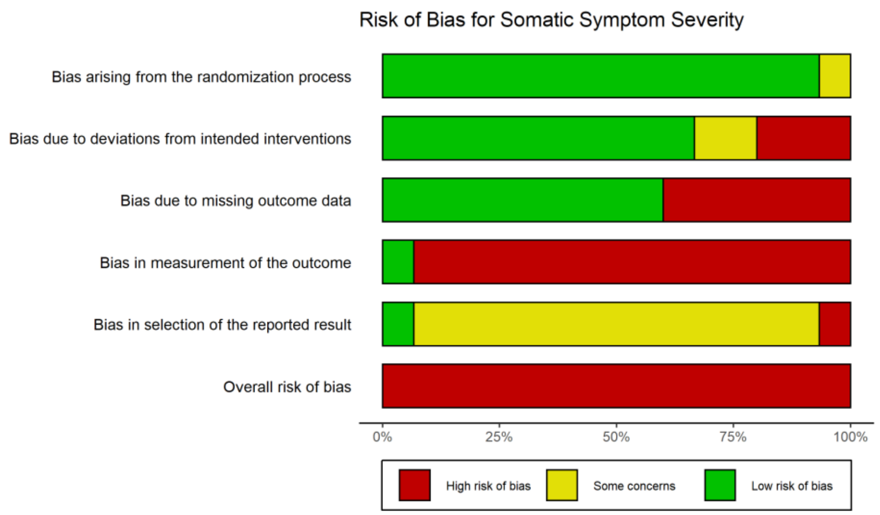

(A)

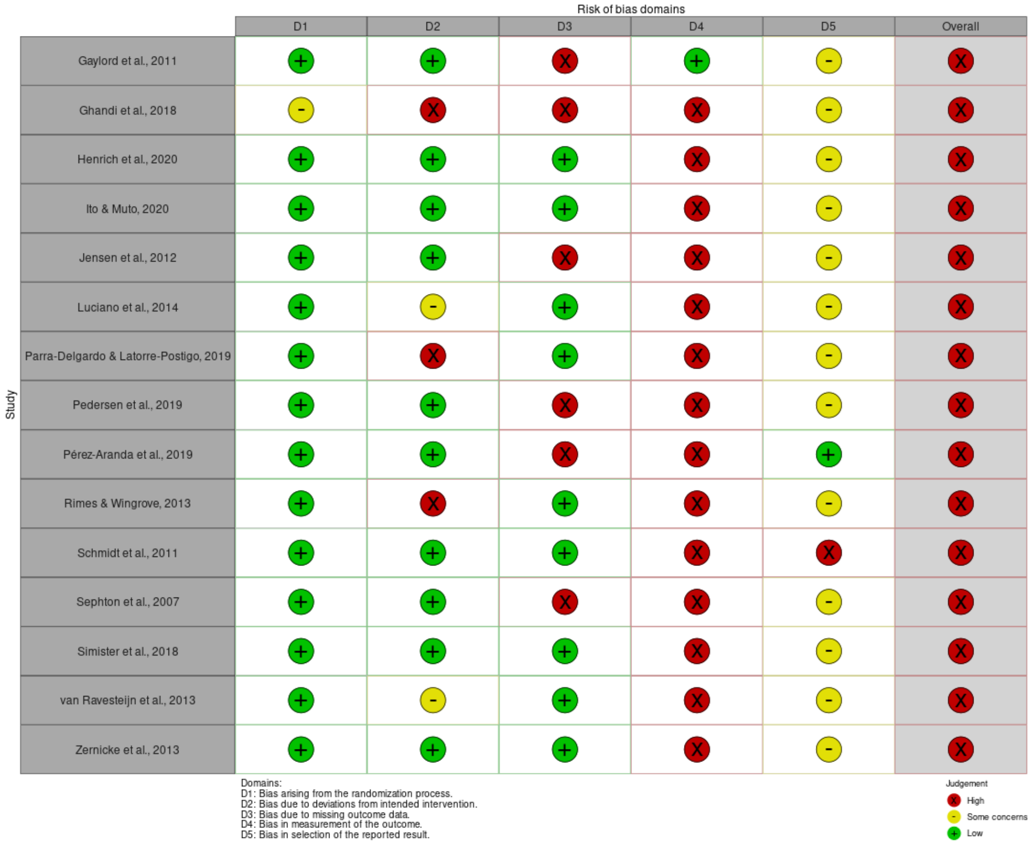

(B)

Note. A: summary plot; B: traffic light plot.

Figure S2

Risk of bias ratings for perceived health status at post-treatment

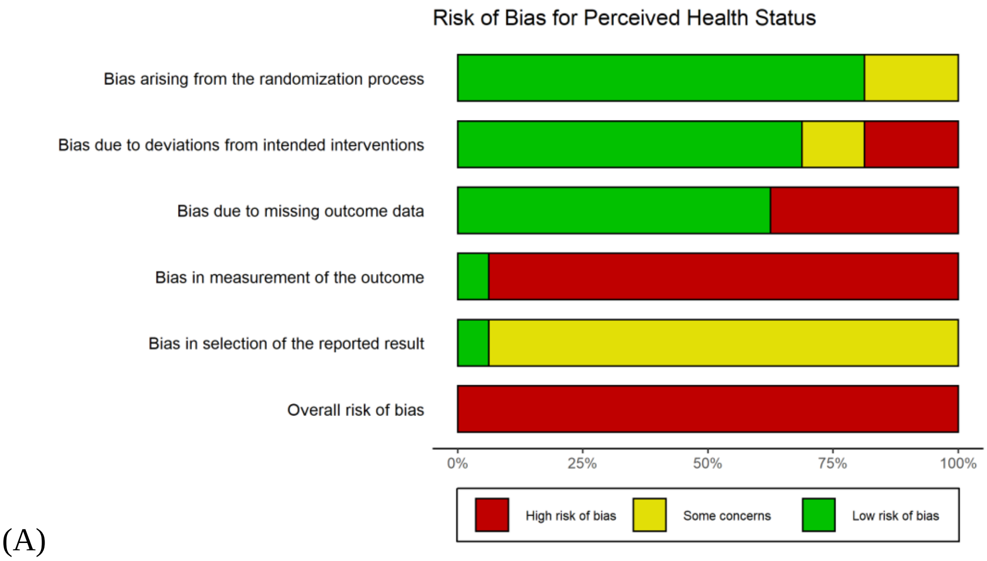

(A)

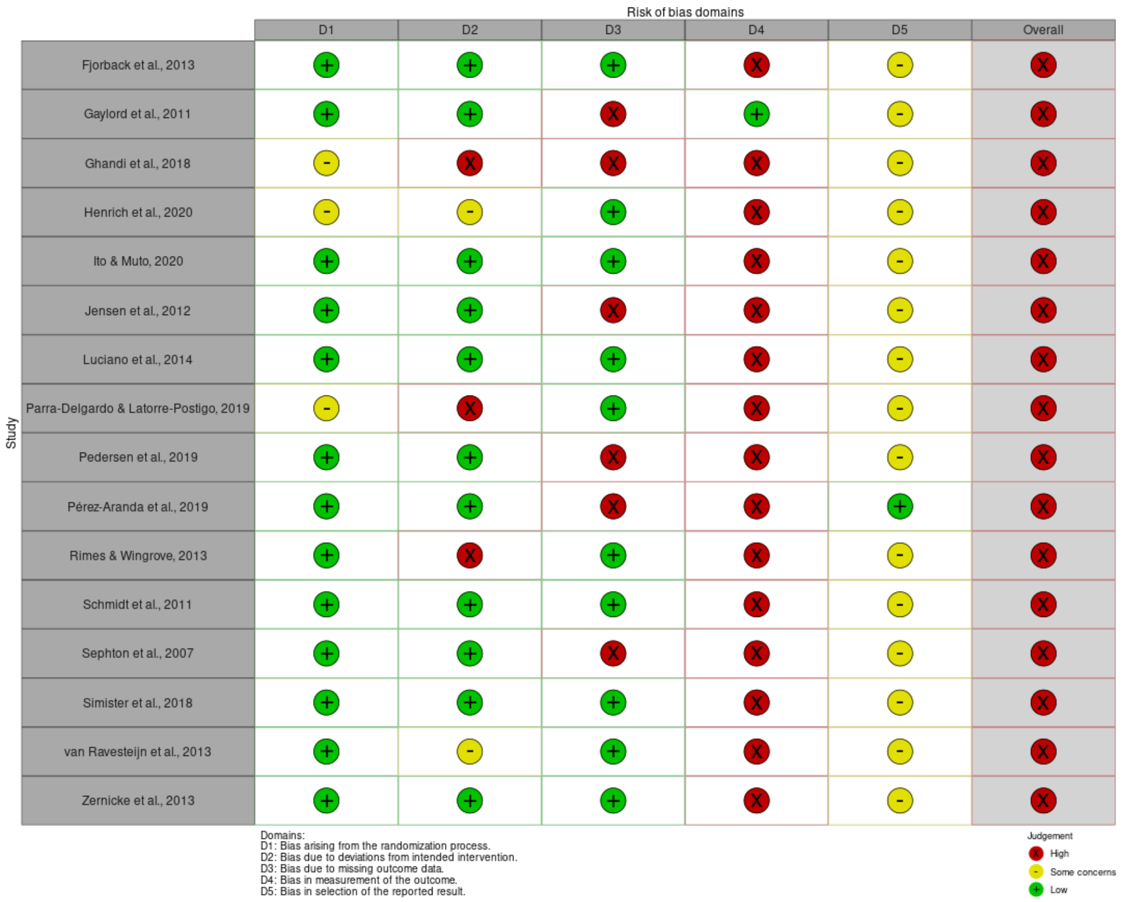

(B)

Note. A: summary plot; B: traffic light plot.

Figure S3

Risk of bias ratings for depression at post-treatment

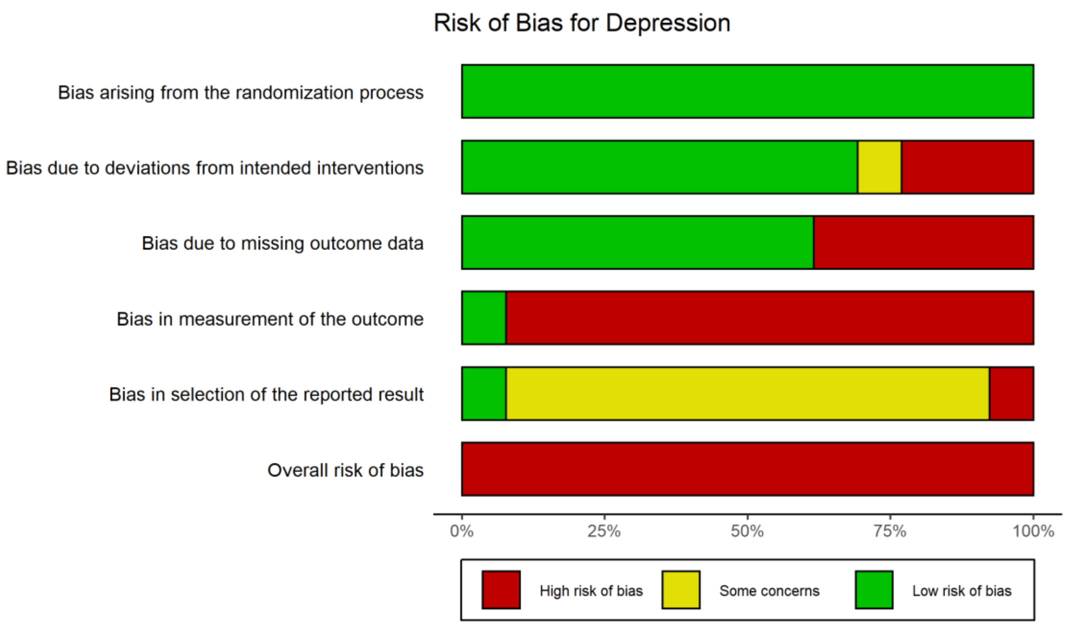

(A)

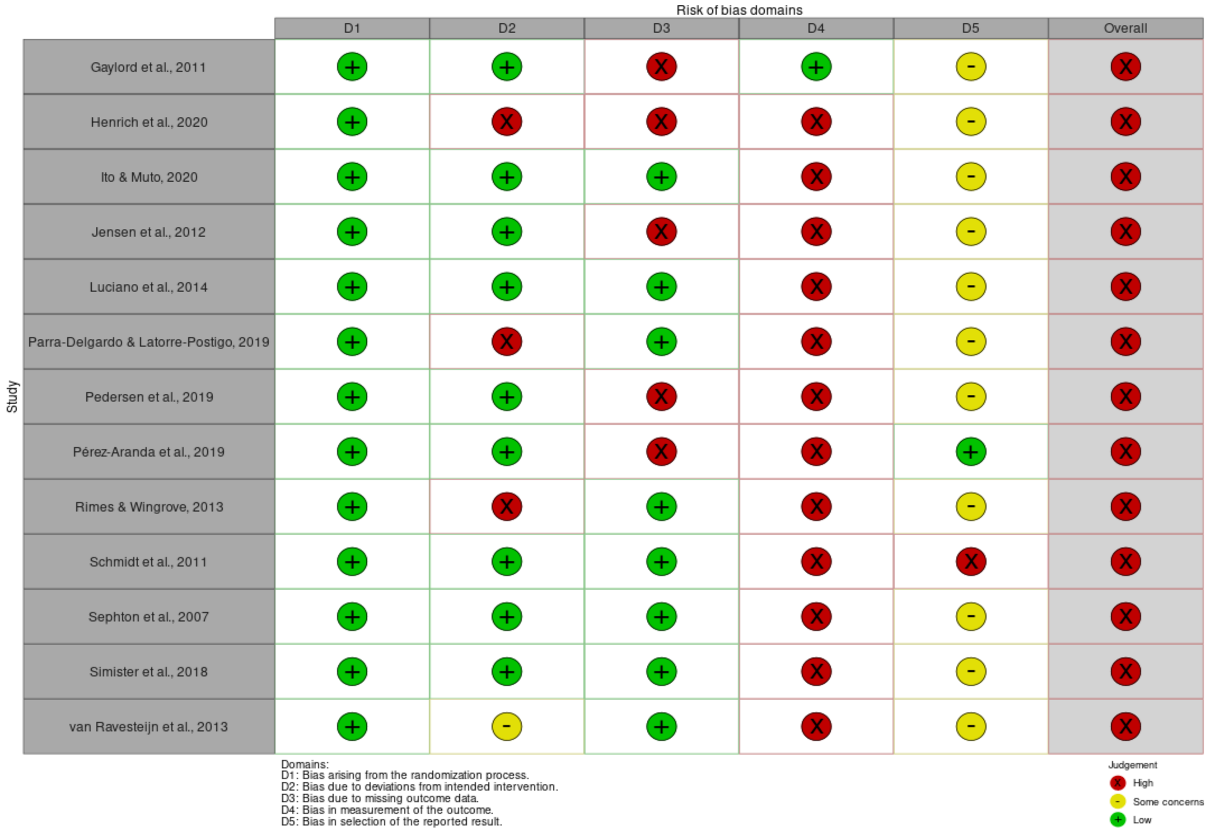

(B)

Note. A: summary plot; B: traffic light plot.

Figure S4

Risk of bias ratings for anxiety at post-treatment

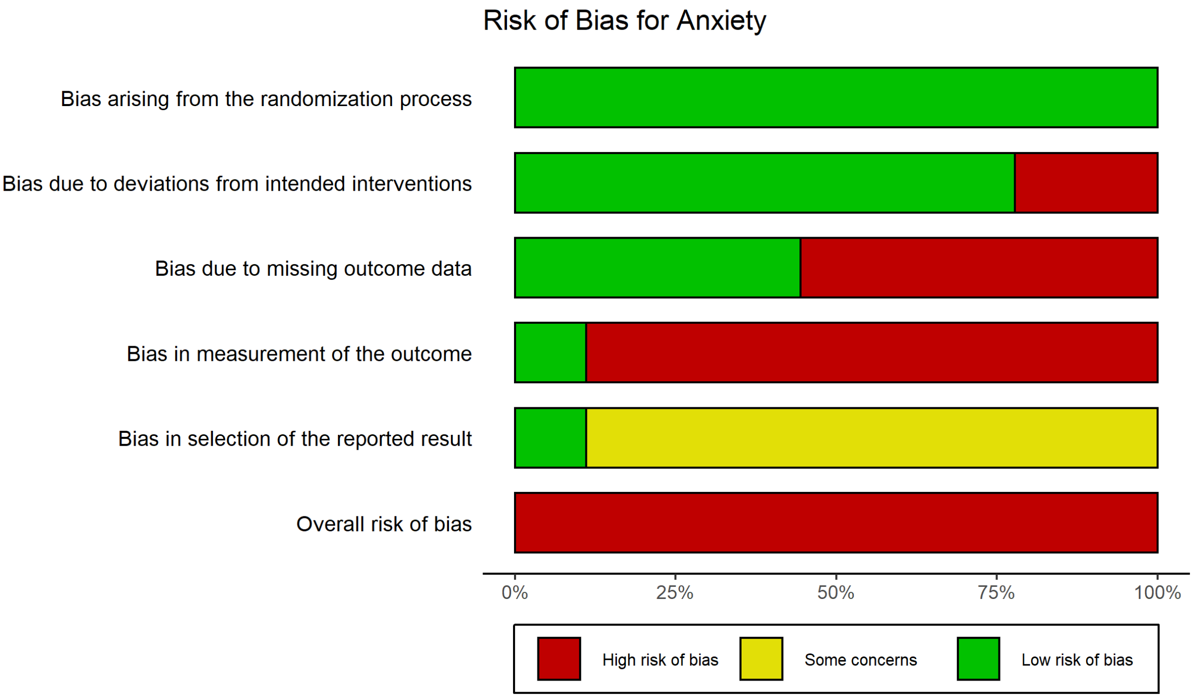

(A)

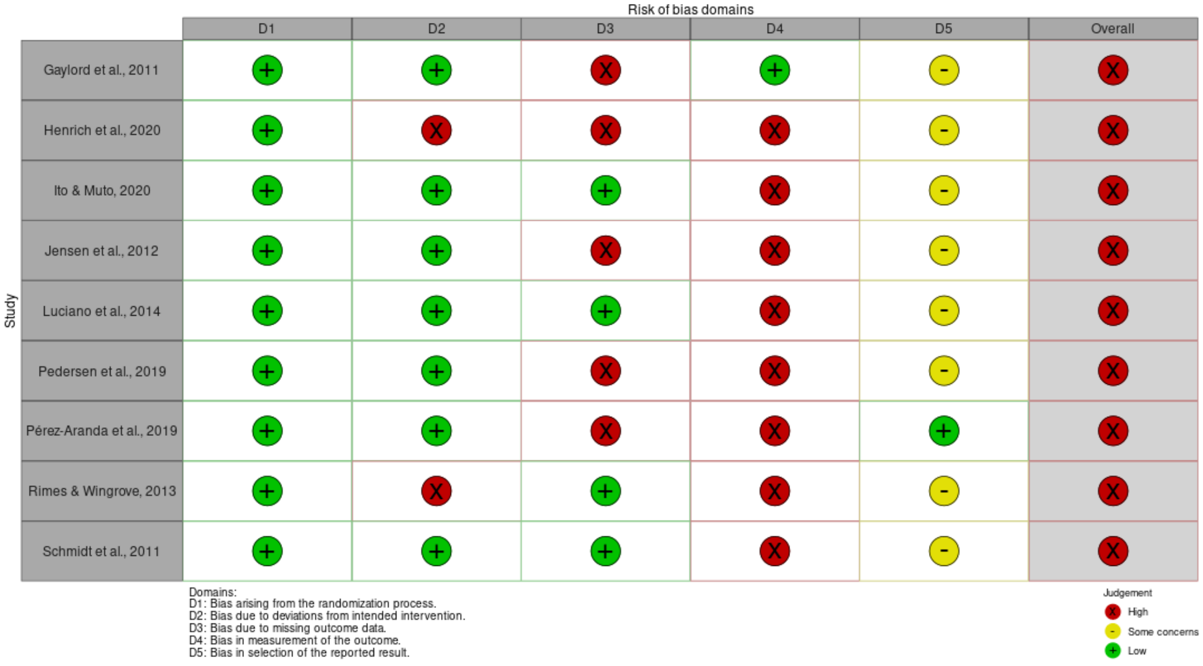

(B)

Note. A: summary plot; B: traffic light plot.

Figure S5

Risk of bias ratings for mindfulness at post-treatment

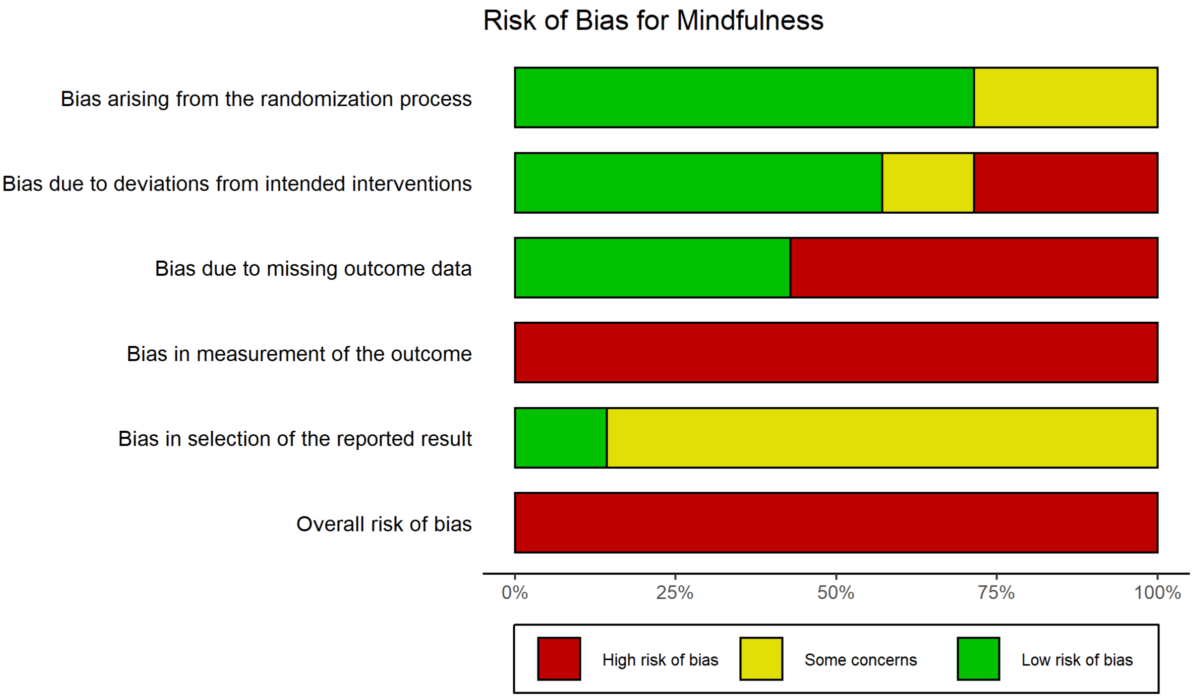

(A)

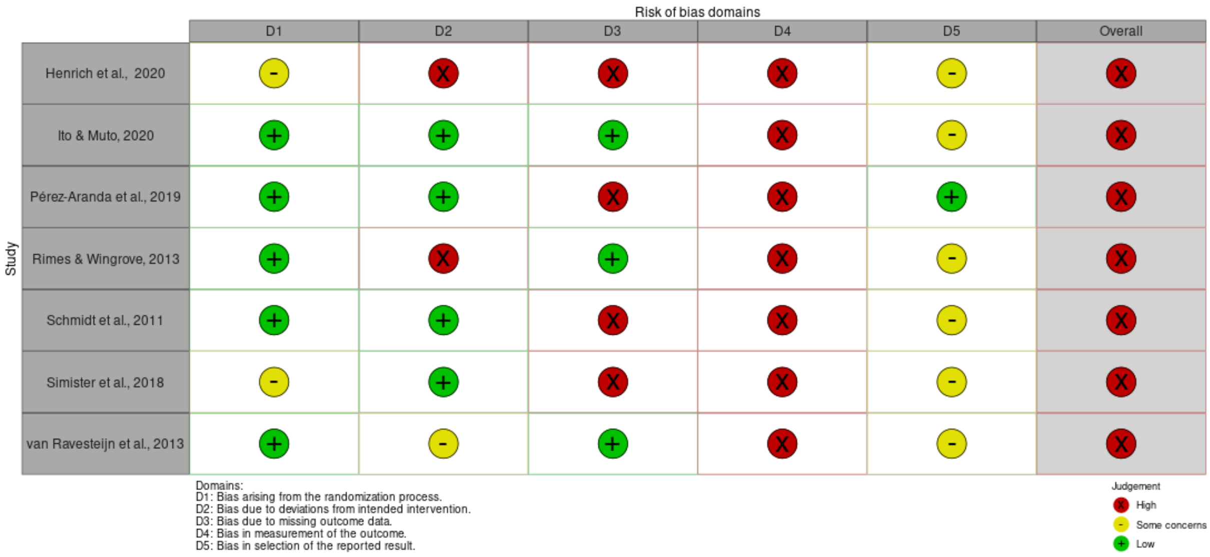

(B)

Note. A: summary plot; B: traffic light plot.

Figure S6

Risk of bias ratings for psychological inflexibility at post-treatment

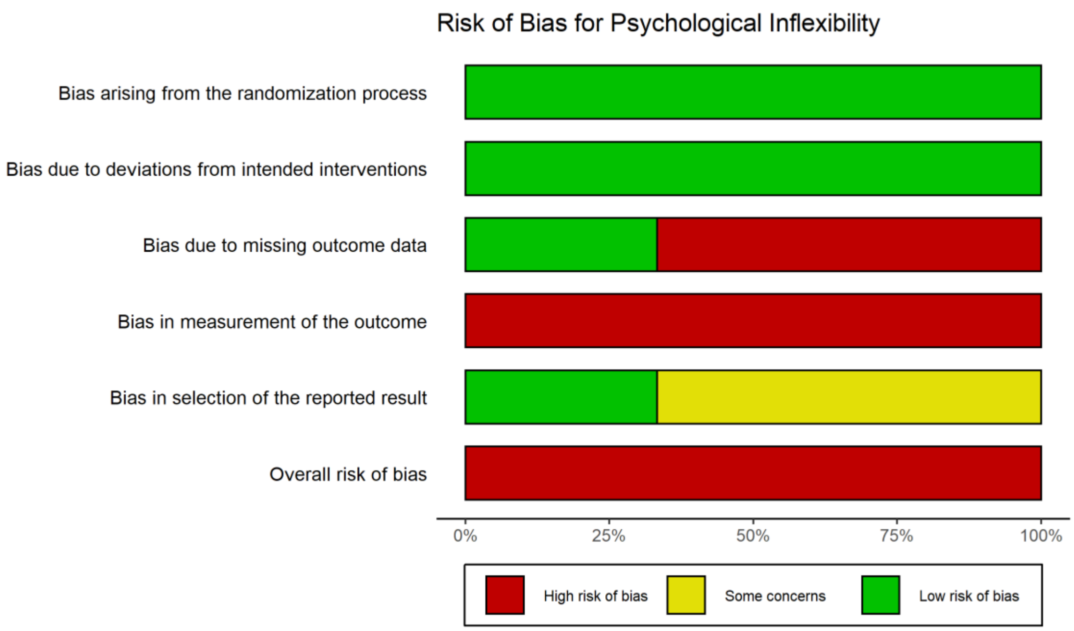

(A)

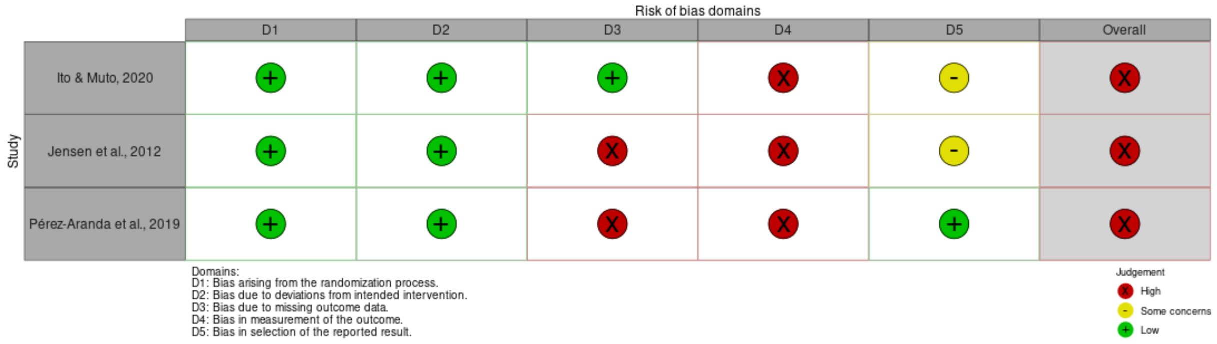

(B)

Note. A: summary plot; B: traffic light plot.

8 R packages

In R version 4.2.1 (50) we used the packages {meta} (51), {metafor} (52), and {dmetar} (53) to perform the effect size aggregation and associated analyses. The package {tidyverse} (54) provided good functions to visualize data. Additionally, we imported {esc} (55), and {car} (56). Furthermore, we used the packages {readxl }(57) to import the data that we prepared in Microsoft Excel (58) sheets. Additionally, we used the package {robvis (59) as well as the corresponding web application (https://mcguinlu.shinyapps.io/robvis/) to depict the risk of bias assessment. Lastly, we used {irr} (60) to calculate interrater reliabilities.

9 Additional information on treatment effects

Figure S7

Forest plots – Post-treatment

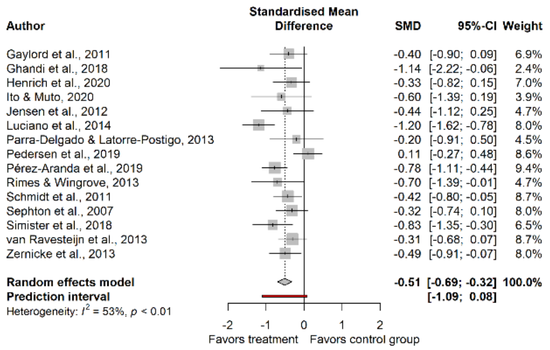

(A)

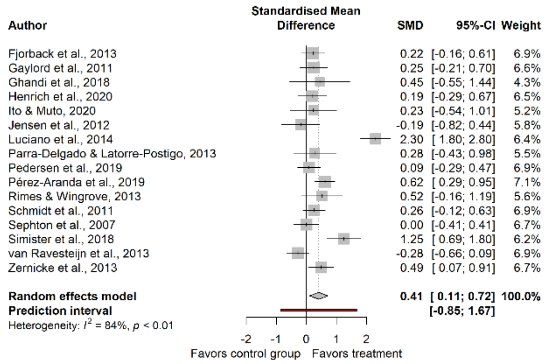

(B)

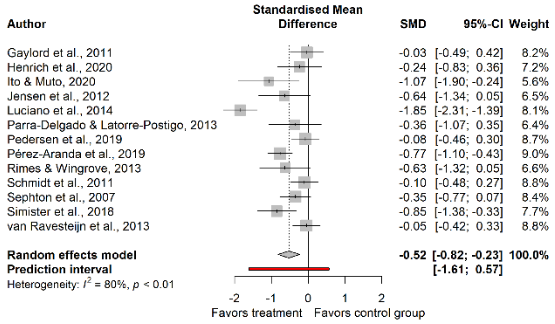

(C)

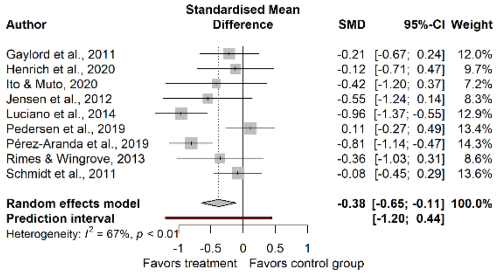

(D)

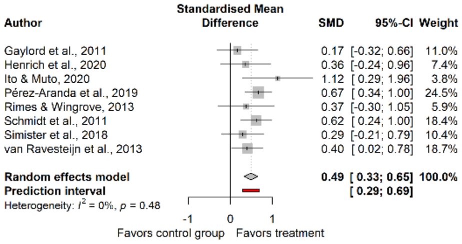

(E)

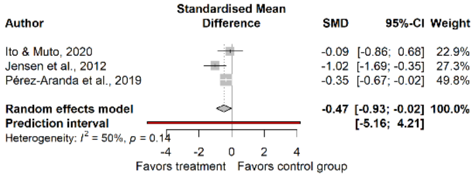

(F)

Note. A: somatic symptom severity at post-treatment; B: perceived health status at post-treatment; C: depression at post-treatment; D: anxiety at post-treatment; E: mindfulness at post-treatment; F: psychological inflexibility at post-treatment. Note: For PHS and mindfulness positive effects indicate efficacy of the treatments, for all other outcomes negative effects indicate efficacy of the treatments.

Figure S8

Forest plots – Short-term follow-up

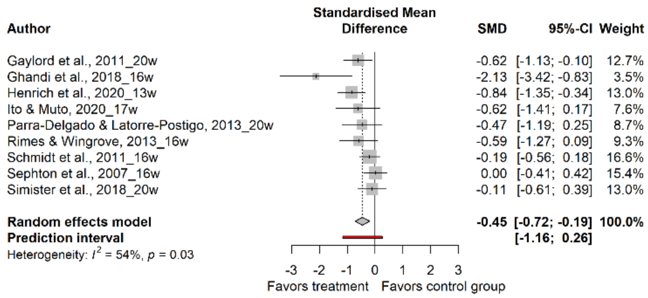

(A)

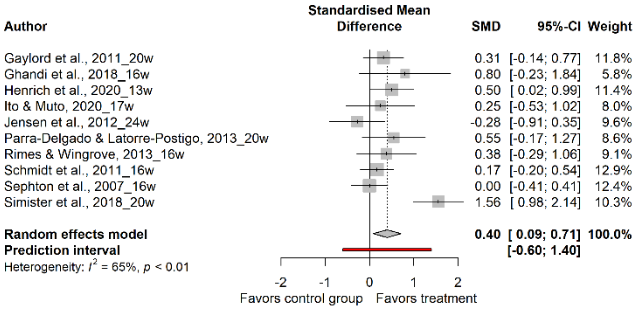

(B)

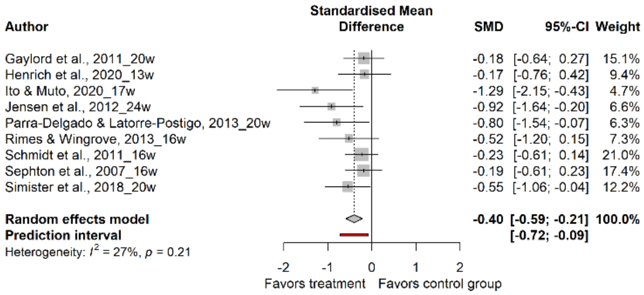

(C)

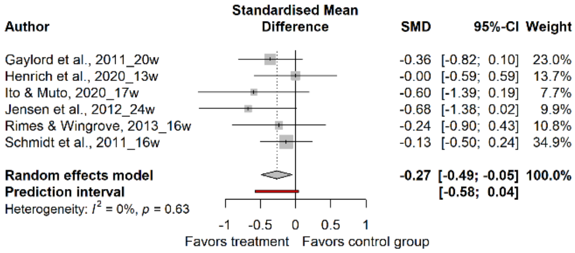

(D)

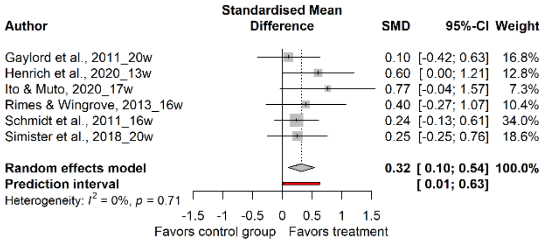

(E)

Note. A: somatic symptom severity at short-term follow-up; B: perceived health status at short-term follow-up; C: depression at short-term follow-up; D: anxiety at short-term follow-up; E: mindfulness at short-term follow-up. Note: For PHS and mindfulness positive effects indicate efficacy of the treatments, for all other outcomes negative effects indicate efficacy of the treatments.

Figure S9

Forest plots – Long-term follow-up

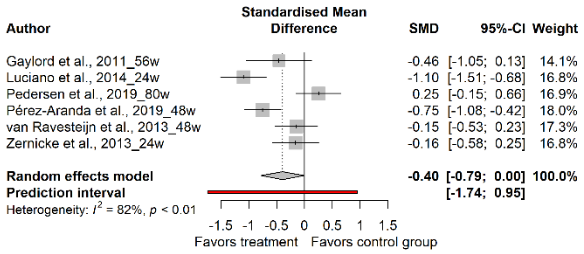

(A)

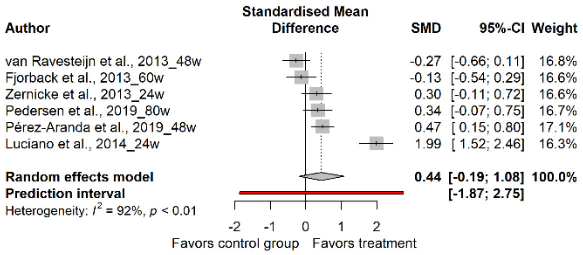

(B)

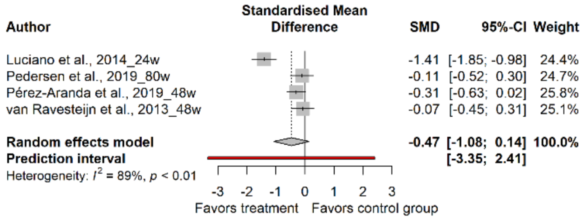

(C)

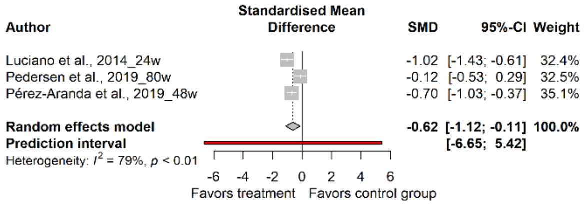

(D)

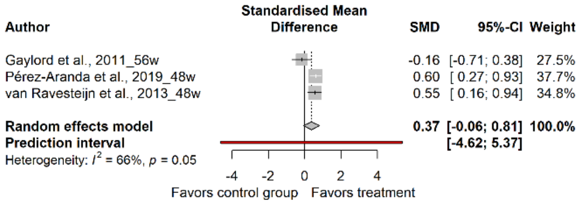

(E)

Note. A: somatic symptom severity at long-term follow-up; B: perceived health status at long-term follow-up; C: depression at long-term follow-up; D: anxiety at long-term follow-up; E: mindfulness at long-term follow-up. Note: For PHS and mindfulness positive effects indicate efficacy of the treatments, for all other outcomes negative effects indicate efficacy of the treatments.

9.1 Descriptive effects for outcomes that were not aggregated

Table S3

Health anxiety (post-treatment)

Study	Instrument	*Ne*	*Nc*	Hedges' *g*	*SE*
Pedersen et al., 2019	WI-7	53	54	0.08	0.19
van Ravesteijn et al., 2013	WI-14	58	53	-0.14	0.19

Note. WI-7 = Whitely Index with 7 items; WI-14 = Whitely Index with 14 items; Ne = number of participants in the experimental group; Nc = number of participants in the control group; SE = standard error.

Table S4

Pain acceptance (post-treatment)

Study	Instrument	*Ne*	*Nc*	Hedges' *g*	*SE*
Luciano et al., 2014	CPAQ	51	53	1.52	0.22
Simister et al., 2018	CPAQ-R	30	31	0.82	0.27

Note. CPAQ = chronic pain catastrophizing scale; CPAQ-R = chronic pain catastrophizing scale revised; Ne = number of participants in the experimental group; Nc = number of participants in the control group; SE = standard error.

9.2 Results moderator analyses

Table S5

Subgroup analyses for type of treatment (post-treatment)

	k	Hedges' g	95%CI	τ^2	I^2	$p_{subgroup}$
Somatic Symptom Severity						0.658
MBCT/MBSR	10	-0.47	-0.62; -0.33	0.0006	0.0%	
ACT	5	-0.59	-1.07; -0.10	0.22	81.8%	
Perceived Health Status						0.294
MBCT/MBSR	11	0.25	0.07; 0.43	0.04	38.2%	
ACT	5	0.75	-0.17; 1.66	1.00	93.6%	
Depression						0.079
MBCT/MBSR	8	-0.31	-0.53; -0.08	0.04	45.0%	
ACT	5	-0.89	-1.51; -0.28	0.40	88.4%	
Anxiety						0.729
MBCT/MBSR	5	-0.34	-0.65; -0.03	0.07	60.3%	
ACT	4	-0.45	-0.96; 0.07	0.19	79.2%	

Note. $k < 3$ for one of the subgroups for mindfulness, and psychological inflexibility.

Table S6

Subgroup analyses for control condition (post-treatment)

	k	Hedges' g	95%CI	τ^2	I^2	$p_{subgroup}$
Somatic Symptom Severity						0.523
Non-specific	8	-0.45	-0.71; -0.20	0.07	58.0%	
Inactive	7	-0.57	-0.85; -0.30	0.07	49.2%	
Perceived Health Status						0.720
Non-specific	9	0.35	0.07; 0.63	0.12	69.8%	
Inactive	7	0.48	-0.15; 1.11	0.65	90.6%	
Depression						0.245
Non-specific	6	-0.35	-0.66; -0.03	0.10	68.7%	
Inactive	7	-0.69	-1.16; -0.21	0.33	84.7%	
Anxiety						0.737
Non-specific	3	-0.31	-0.85; 0.23	0.44	84.9%	
Inactive	6	-0.42	-0.75; -0.09	0.29	55.1%	
Mindfulness						0.425
Non-specific	4	0.43	0.20; 0.66	0.01	11.3%	
Inactive	4	0.58	0.30; 0.85	<0.001	0.0%	

Note. $k < 3$ for one of the subgroups for psychological inflexibility; inactive = wait-list; non-specific = EC, psychological placebo, TAU.

Table S7

Subgroup analyses for diagnosis (post-treatment)

	k	Hedges' g	95%CI	τ^2	I^2	$p_{subgroup}$
Somatic Symptom Severity						0.396
IBS	5	-0.47	-0.72; -0.23	0.00	0.0%	
FM	7	-0.63	-0.89; -0.37	0.07	54.9%	
Perceived Health Status						0.349
IBS	5	0.32	0.08; 0.56	0.00	0.0%	
FM	7	0.65	0.008; 1.29	0.68	91.2%	
Depression						0.327
IBS	3	-0.36	-0.90; 0.19	0.13	56.7%	
FM	7	-0.71	-1.15; -0.27	0.28	84.2%	
Anxiety						0.156
IBS	3	-0.22	-0.55; 0.11	0.00	0.0%	
FM	4	-0.60	-1.01; -0.19	0.13	75.1%	
Mindfulness						0.657
IBS	3	0.45	-0.03; 0.94	0.08	46.6%	
FM	3	0.58	0.35; 0.80	0.00	0.0%	

Note. $k < 3$ for one of the subgroups for psychological inflexibility.

Table S8

Subgroup analyses for therapy intensity (post-treatment)

	k	Hedges' g	95%CI	τ^2	I^2	$p_{subgroup}$
Somatic Symptom Severity						0.712
High intensity	12	-0.49	-0.71; -0.28	0.08	60.1%	
Low intensity	3	-0.57	-0.92; -0.22	0.01	0.0%	
Perceived Health Status						0.634
High intensity	13	0.38	0.03; 0.73	0.35	85.3%	
Low intensity	3	0.57	-0.13; 1.27	0.28	77.5%	
Depression						0.496
High intensity	10	-0.48	-0.83; -0.12	0.26	83.5%	
Low intensity	3	-0.68	-1.16; -0.21	0.07	41.3%	
Mindfulness						0.911
High intensity	5	0.50	0.32; 0.68	0.00	0.0%	
Low intensity	3	0.48	0.09; 0.86	0.02	32.4%	

Note. $k < 3$ for one of the subgroups for anxiety, and psychological inflexibility.

Table S9

Meta-regression analyses for therapy dosis (effect on outcomes at post-treatment)

Outcome	$\tau^2_{unexplained}$	I^2	R^2	$p_{moderator}$	Regression weight	Intercept
Somatic Symptom Severity	0.05	46.69%	22.58%	0.109	0.0004	-0.95
Perceived Health Status	0.31	84.58%	0.00%	0.526	0.65	-0.0002
Depression	0.20	77.66%	14.51%	0.126	-1.23	0.0006
Anxiety	0.08	58.82%	18.93%	0.22	-0.85	0.0004
Mindfulness	0.0009	1.57%	0.00%	0.481	0.71	-0.0002

Note. $\tau^2_{unexplained}$ = estimated amount of residual heterogeneity; I^2 = residual heterogeneity/unaccounted variability; R^2 = amount of heterogeneity accounted for

Figure S10

Bubble plots – meta-regression analysis for therapy dosis

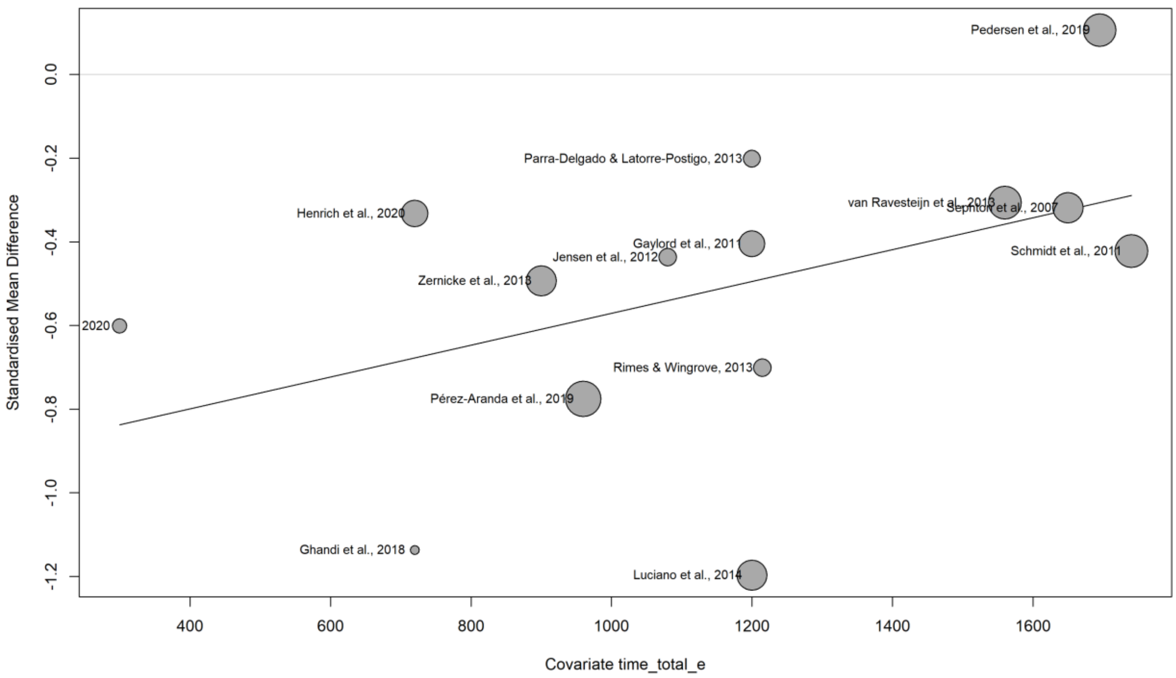

(A)

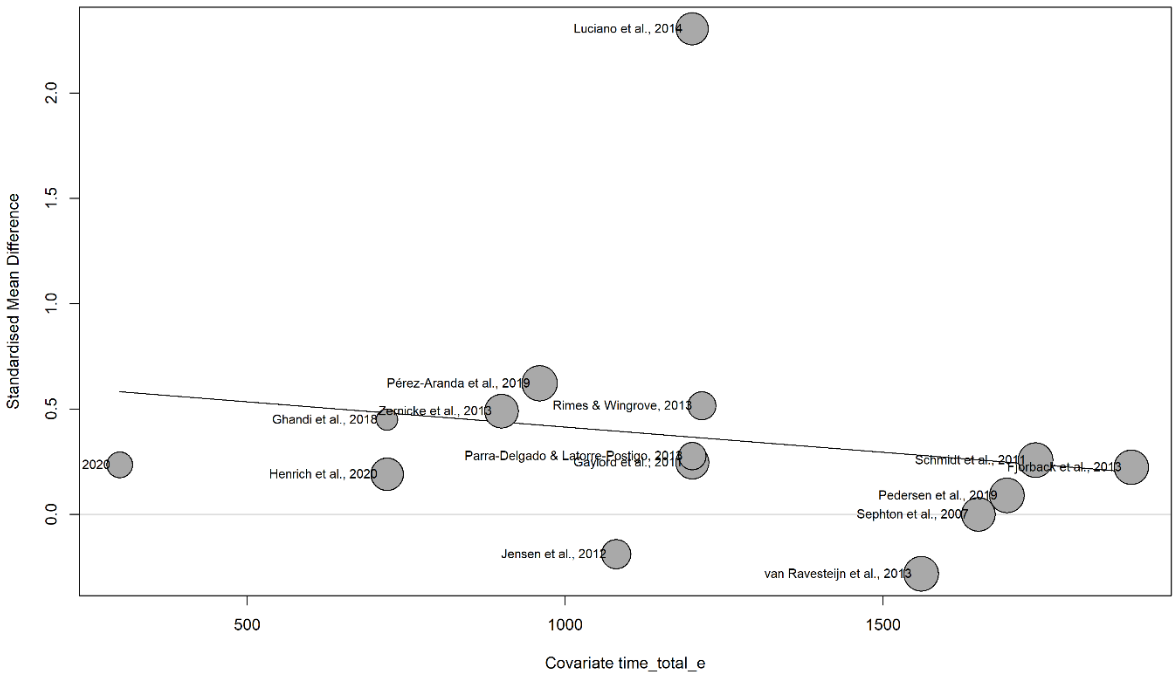

(B)

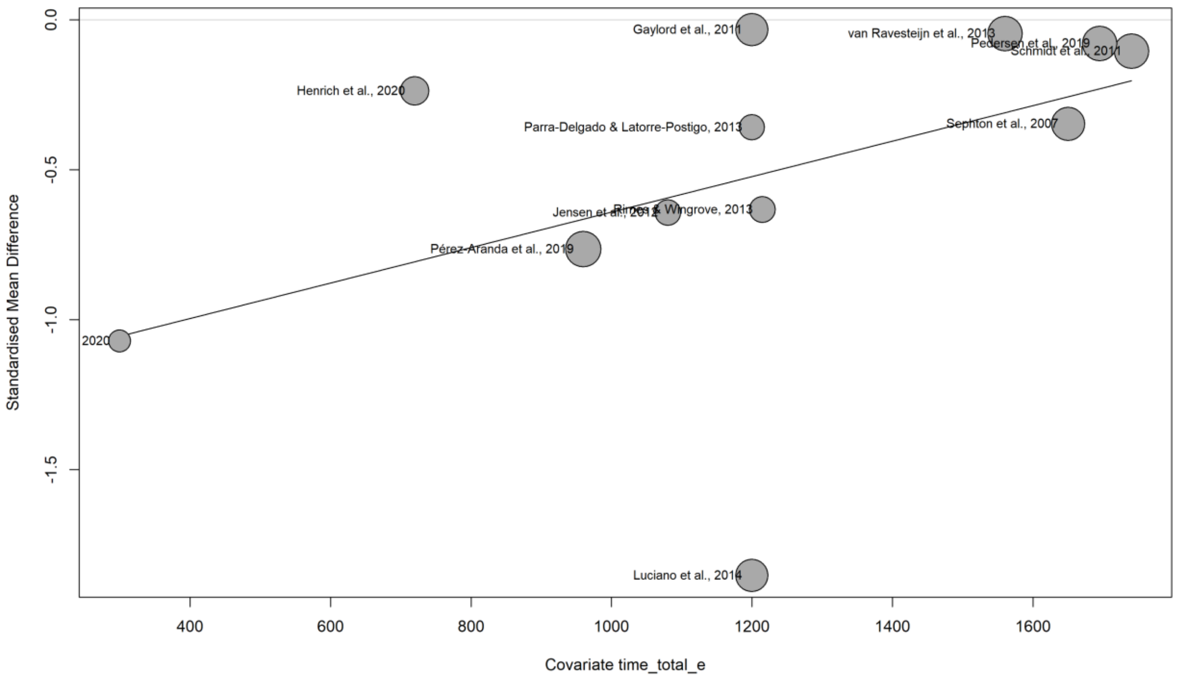

Gaylord et al., 2011
van Ravesteijn et al., 2013
Pedersen et al., 2019
Schmidt et al., 2011
Henrich et al., 2020
Parra-Delgado & Latorre-Postigo, 2013
Sephton et al., 2007
Rimes & Wingrove, 2013
Pérez-Aranda et al., 2019
2020
Luciano et al., 2014
Standardised Mean Difference
Covariate time_total_e

(C)

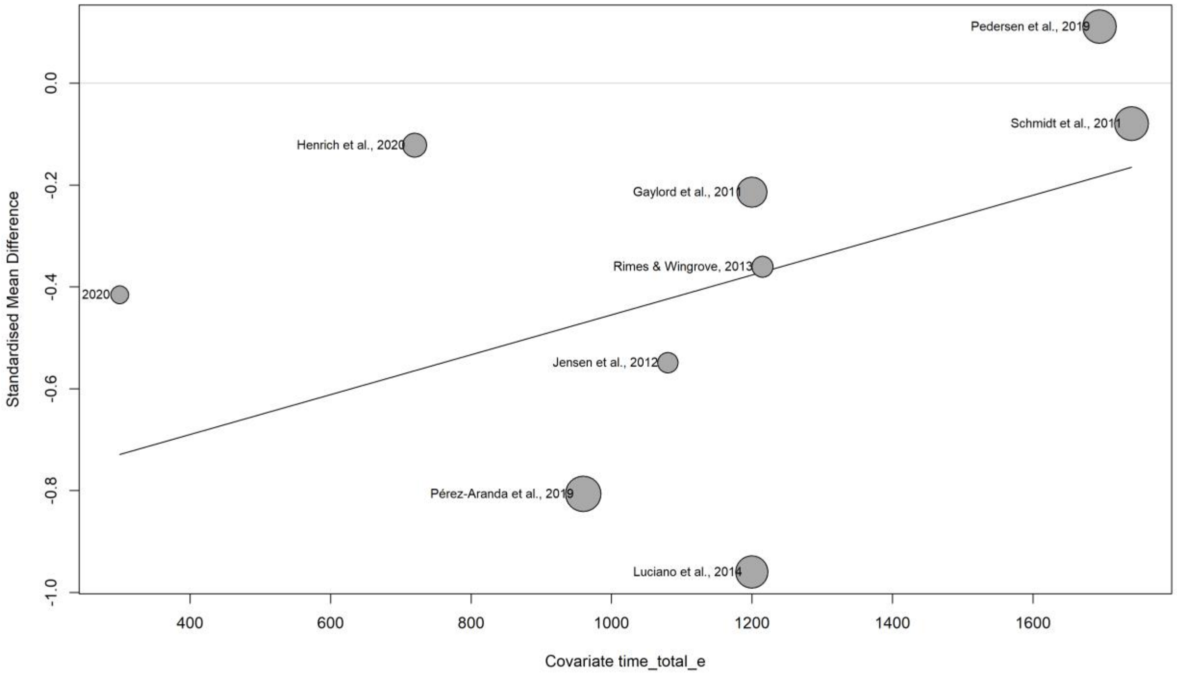

Pedersen et al., 2019
Schmidt et al., 2011
Henrich et al., 2020
Gaylord et al., 2011
Rimes & Wingrove, 2013
2020
Jensen et al., 2012
Pérez-Aranda et al., 2019
Luciano et al., 2014
Standardised Mean Difference
Covariate time_total_e

(D)

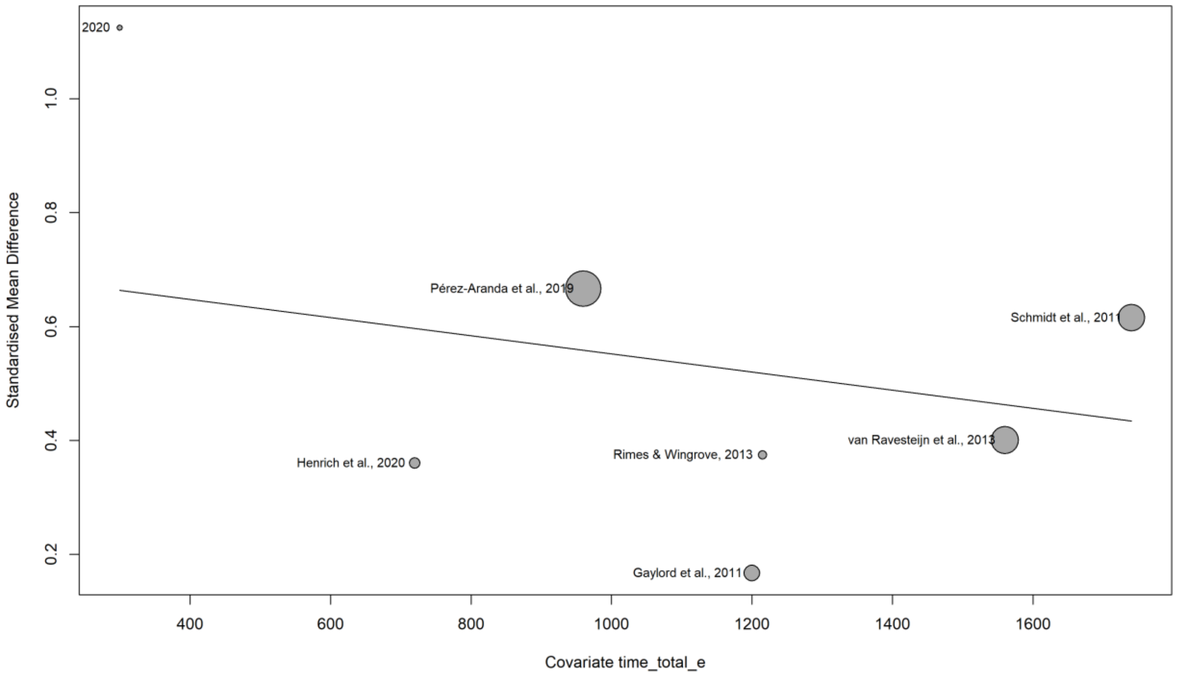

(E)

Note. A: bubble plot for the effect of psychotherapy dosis on somatic symptom severity at post-treatment; B: bubble plot for the effect of psychotherapy dosis on perceived health status at post-treatment; C: bubble plot for the effect of psychotherapy dosis on depression at post-treatment; D: bubble plot for the effect of psychotherapy dosis on anxiety at post-treatment; E: bubble plot for the effect of psychotherapy dosis on mindfulness at post-treatment. Note: Simister et al. (61) was not included as time_total_e could not be calculated.

Table S10

Meta-regression analyses for number of sessions (effect on outcomes at post-treatment)

Outcome	$\tau^2_{unexplained}$	I^2	R^2	$p_{moderator}$	Regression weight	Intercept
Somatic Symptom Severity	0.07	53.36%	0.00%	0.458	-0.80	0.03
Perceived Health Status	0.33	85.51%	0.00%	0.485	0.84	-0.05
Depression	0.21	78.58%	6.77%	0.225	-1.17	0.08
Anxiety	0.11	67.30%	0.00%	0.746	-0.53	0.02
Mindfulness	0.003	4.28%	0.00%	0.478	0.74	-0.03

Note: $\tau^2_{unexplained}$ = estimated amount of residual heterogeneity; I^2 = residual heterogeneity/unaccounted variability; R^2 = amount of heterogeneity accounted for.

Figure S11

Bubble plots – meta-regression analysis for number of sessions

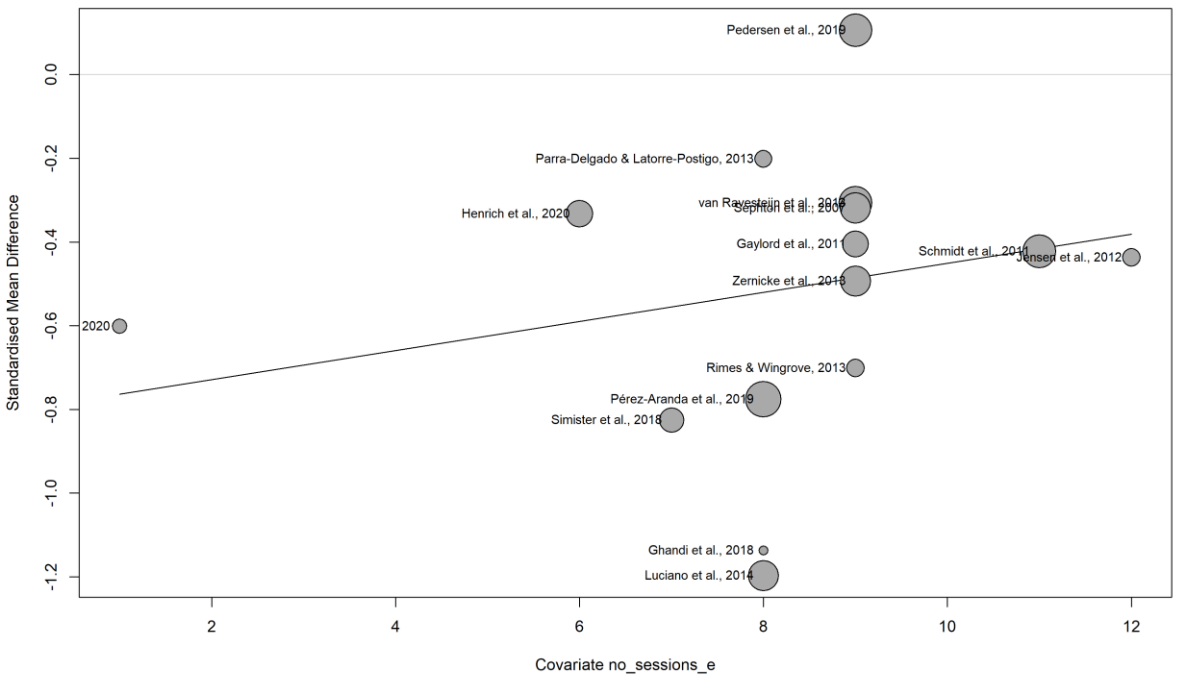

(A)

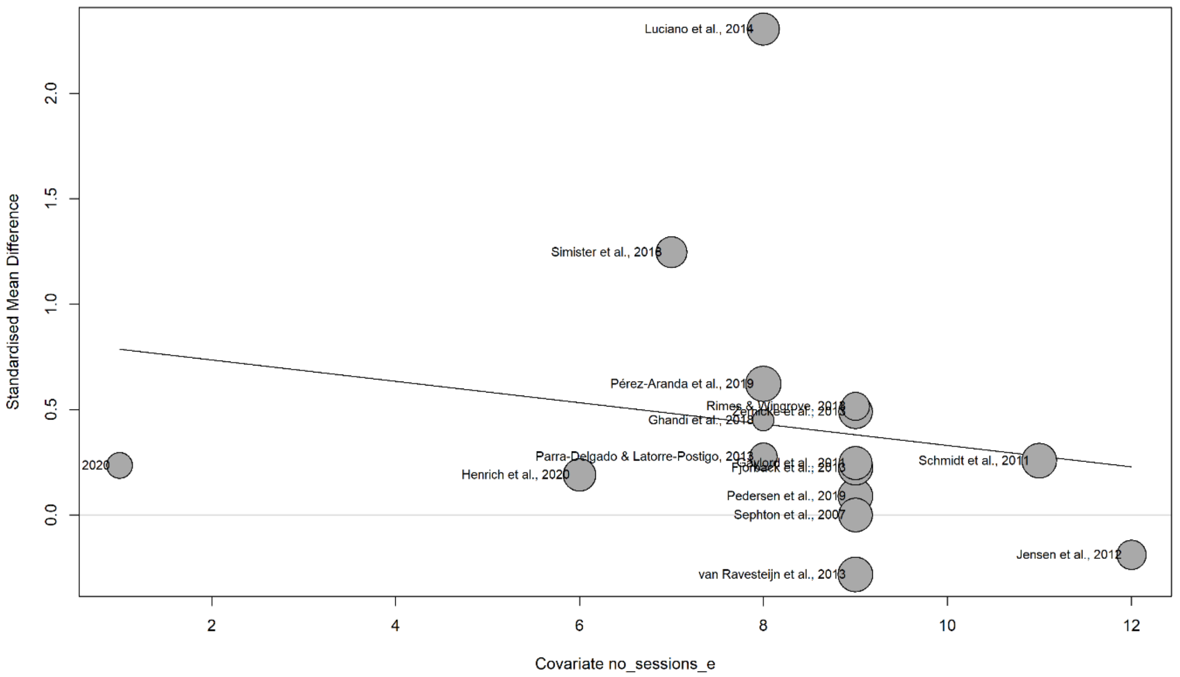

(B)

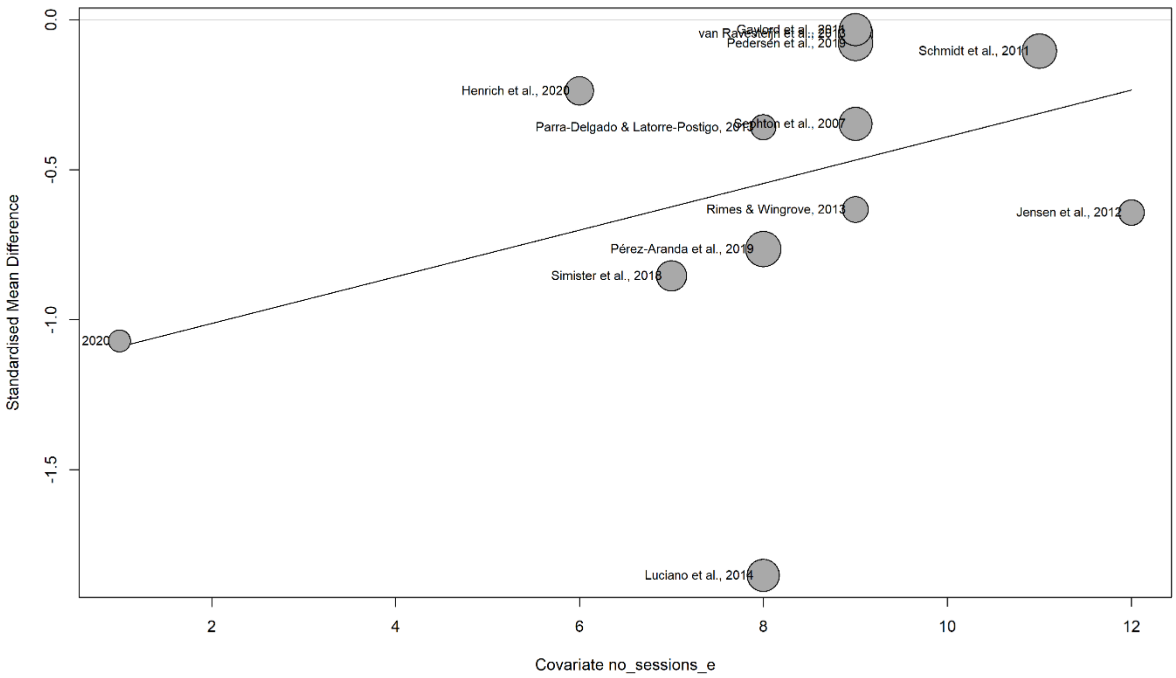
Standardised Mean Difference
Covariate no_sessions_e
Gaylord et al., 2011
van Ravesteijn et al., 2013
Pedersen et al., 2019
Schmidt et al., 2011
Henrich et al., 2020
Parra-Delgado & Latorre-Postigo, 2013
Sephton et al., 2007
Rimes & Wingrove, 2013
Jensen et al., 2012
Pérez-Aranda et al., 2019
Simister et al., 2018
2020
Luciano et al., 2014

(C)

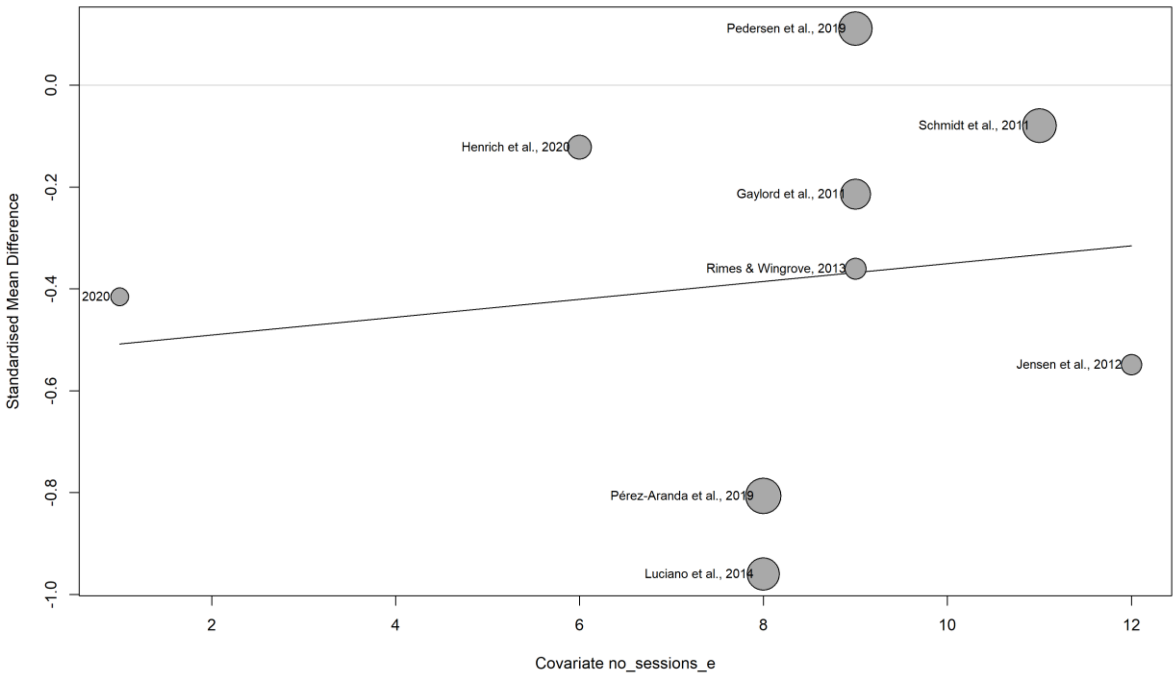
Standardised Mean Difference
Covariate no_sessions_e
Pedersen et al., 2019
Schmidt et al., 2011
Henrich et al., 2020
Gaylord et al., 2011
Rimes & Wingrove, 2013
2020
Jensen et al., 2012
Pérez-Aranda et al., 2019
Luciano et al., 2014

(D)

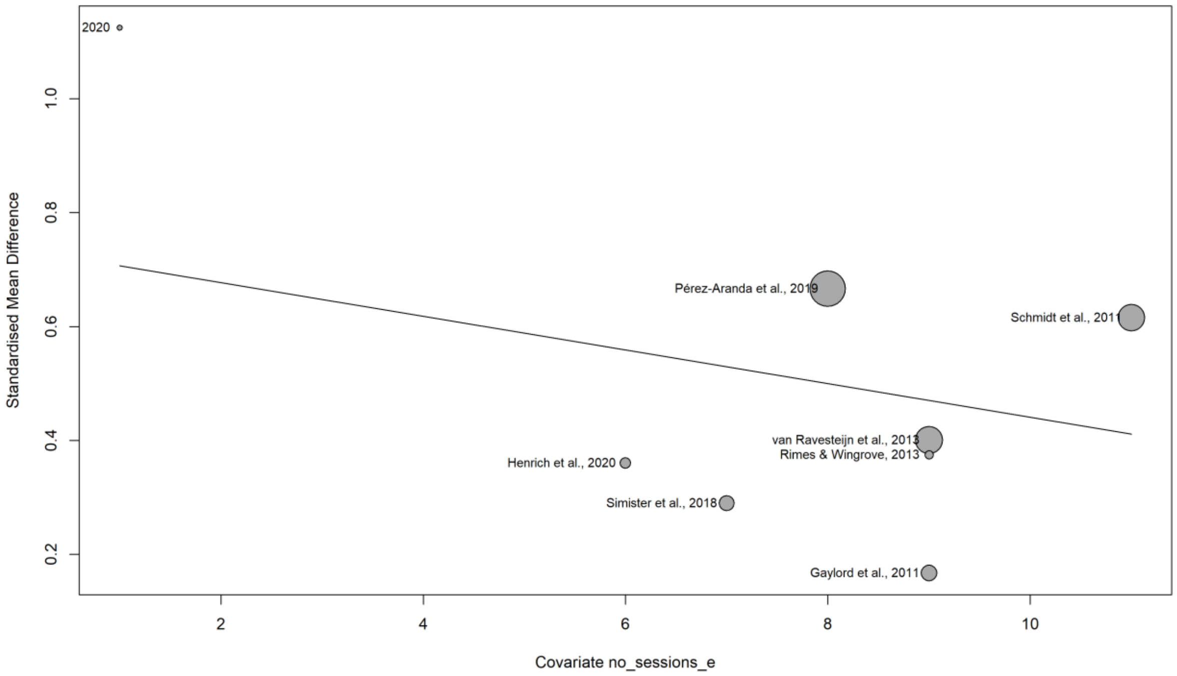

(E)

Note. A: bubble plot for the effect of number of sessions on somatic symptom severity at post-treatment; B: bubble plot for the effect of number of sessions on perceived health status at post-treatment; C: bubble plot for the effect of number of sessions on depression at post-treatment; D: bubble plot for the effect of number of sessions on anxiety at post-treatment; E: bubble plot for the effect of number of sessions on mindfulness at post-treatment. Note: Simister et al. (61) was not included as time_total_e could not be calculated.

9.3 Results sensitivity analyses

Table S11

Sensitivity analyses comparing fixed effect and random effects models (post-treatment)

Outcome	*k*	Model	Hedges' *g*	95%CI
Somatic symptom severity	15	Random effects	-0.51	-0.69; -0.32
		Fixed effect	-0.50	-0.62; -0.37
Perceived Health status	16	Random effects	0.41	0.11; 0.72
		Fixed effect	0.37	0.26; 0.49
Depression	13	Random effects	-0.52	-0.82; -0.23
		Fixed effect	-0.47	-0.60; -0.34
Anxiety	9	Random effects	-0.38	-0.65; -0.11
		Fixed effect	-0.39	-0.55; -0.24
Mindfulness	8	Random effects	0.49	0.33; 0.65
		Fixed effect	0.49	0. .33; 0.65
Psychological inflexibility	3	Random effects	-0.47	-0.93; -0.02
		Fixed effect	-0.43	-0.70; -0.15

Note. *k* = number of studies.

Table S12

Sensitivity analyses comparing PHS with the prioritized measures and the alternative measures

Outcome	*k*	PHS Measures	Hedges' *g*	95%CI	I^2	95%CI
Post treatment	16	Prioritized	0.41	0.11; 0.72	83.7%	74.8; 89.4
		Alternative measures	0.42	0.20; 0.65	72.0%	53.8; 83.1
Short-term follow-up	10	Prioritized	0.40	0.09; 0.71	65.1%	31.6; 82.2
		Alternative measures	0.49	0.19; 0.79	63.5%	27.8; 81.5
Long-term follow-up	6	Prioritized	0.44	-0.19; 1.08	91.9%	85.2; 95.6
		Alternative measures	0.22	-0.19; 0.63	84.1%	67.1; 92.4

Note. *k* = number of studies.

Table S13

Outlier analyses

	k	Hedges' g	95%CI	I^2	95%CI	95%PI
Somatic Symptom Severity						
With outliers	15	-0.51	-0.69; -0.32	52.8%	15.1; 73.8	-1.09; 0.08
Without outliers	13	-0.50	-0.63; -0.37	0.0%	0.0; 56.6	-0.65; -0.35
Perceived Health Status						
With outliers	16	0.41	0.11; 0.72	83.7%	74.8; 89.4	-0.85; 1.67
Without outliers	14	0.33	0.15; 0.50	41.2%	0.0; 68.7	-0.17; 0.82
Depression						
With outliers	13	-0.52	-0.82; -0.23	79.8%	66.2; 87.9	-1.61; 0.57
Without outliers	12	-0.38	-0.57; -0.18	50.0%	3.0; 74.2	-0.95; 0.20

Note. k = number of studies.

Figure S12

Results for the influence analyses for somatic symptom severity at post-treatment

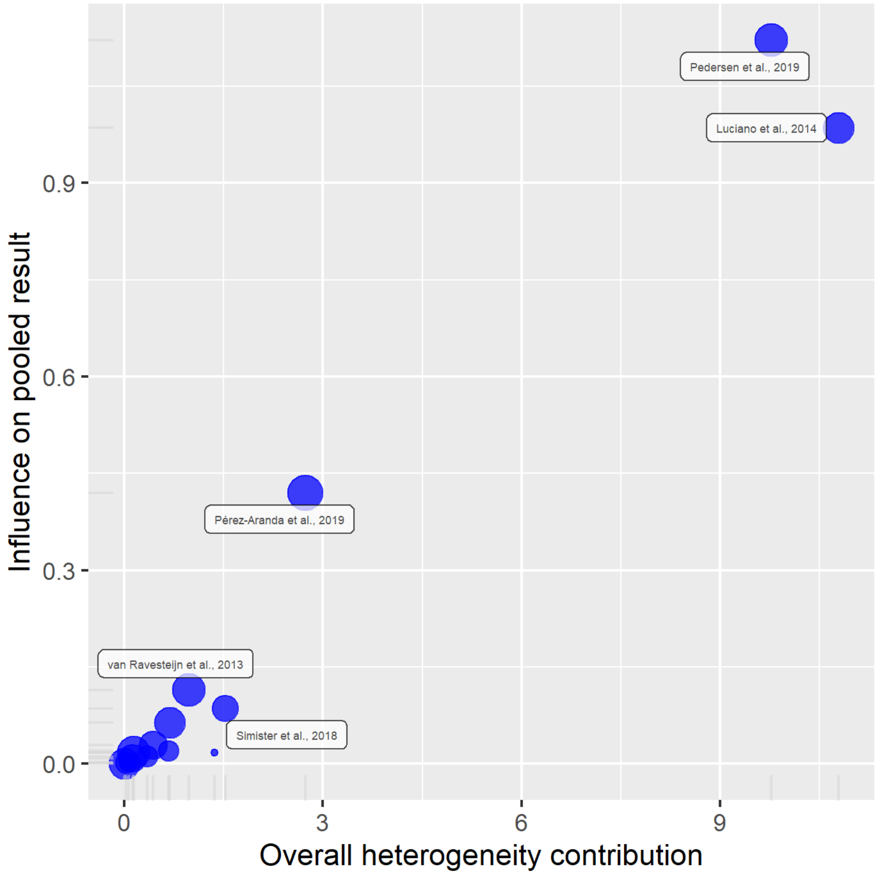

(A)

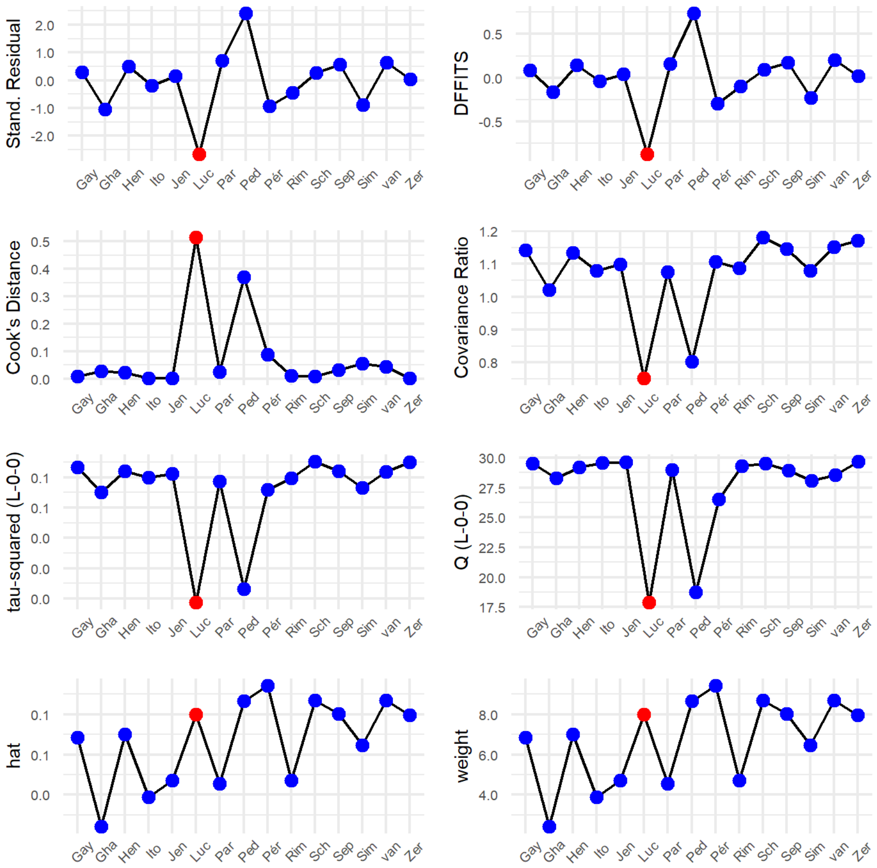

Stand. Residual
DFFITS
Cook's Distance
Covariance Ratio
tau-squared (L-0-0)
Q (L-0-0)
hat
weight
Gay Gha Hen Ito Jen Luc Par Ped Pér Rim Sch Sep Sim van Zer

(B)

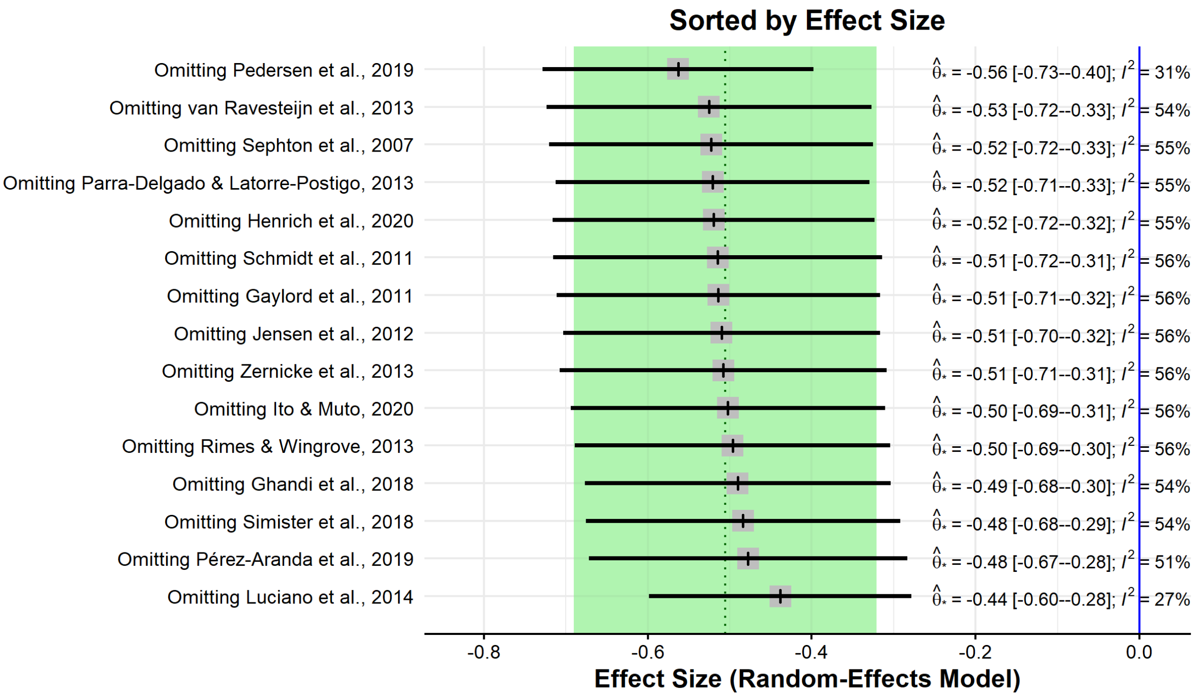

Sorted by Effect Size
Omitting Pedersen et al., 2019
Omitting van Ravesteijn et al., 2013
Omitting Sephton et al., 2007
Omitting Parra-Delgado & Latorre-Postigo, 2013
Omitting Henrich et al., 2020
Omitting Schmidt et al., 2011
Omitting Gaylord et al., 2011
Omitting Jensen et al., 2012
Omitting Zernicke et al., 2013
Omitting Ito & Muto, 2020
Omitting Rimes & Wingrove, 2013
Omitting Ghandi et al., 2018
Omitting Simister et al., 2018
Omitting Pérez-Aranda et al., 2019
Omitting Luciano et al., 2014
$\hat{\theta}_{*}$ = -0.56 [-0.73--0.40]; I^2 = 31%
$\hat{\theta}_{*}$ = -0.53 [-0.72--0.33]; I^2 = 54%
$\hat{\theta}_{*}$ = -0.52 [-0.72--0.33]; I^2 = 55%
$\hat{\theta}_{*}$ = -0.52 [-0.71--0.33]; I^2 = 55%
$\hat{\theta}_{*}$ = -0.52 [-0.72--0.32]; I^2 = 55%
$\hat{\theta}_{*}$ = -0.51 [-0.72--0.31]; I^2 = 56%
$\hat{\theta}_{*}$ = -0.51 [-0.71--0.32]; I^2 = 56%
$\hat{\theta}_{*}$ = -0.51 [-0.70--0.32]; I^2 = 56%
$\hat{\theta}_{*}$ = -0.51 [-0.71--0.31]; I^2 = 56%
$\hat{\theta}_{*}$ = -0.50 [-0.69--0.31]; I^2 = 56%
$\hat{\theta}_{*}$ = -0.50 [-0.69--0.30]; I^2 = 56%
$\hat{\theta}_{*}$ = -0.49 [-0.68--0.30]; I^2 = 54%
$\hat{\theta}_{*}$ = -0.48 [-0.68--0.29]; I^2 = 54%
$\hat{\theta}_{*}$ = -0.48 [-0.67--0.28]; I^2 = 51%
$\hat{\theta}_{*}$ = -0.44 [-0.60--0.28]; I^2 = 27%
-0.8 -0.6 -0.4 -0.2 0.0
Effect Size (Random-Effects Model)

(C)

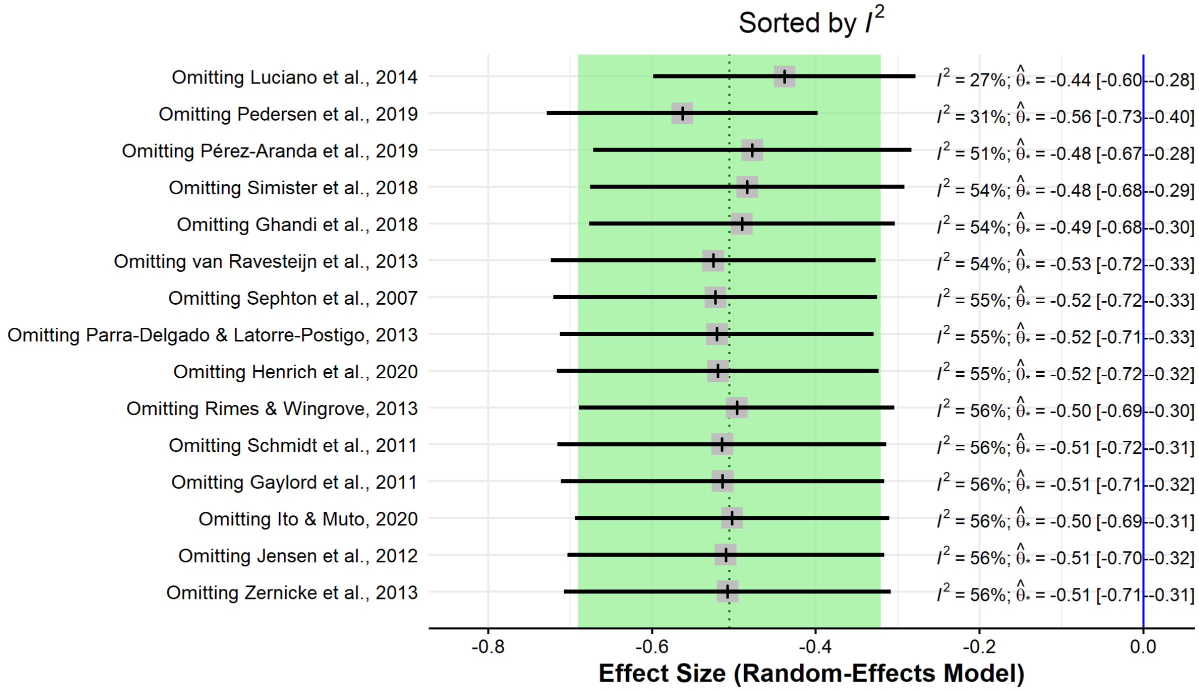

(D)

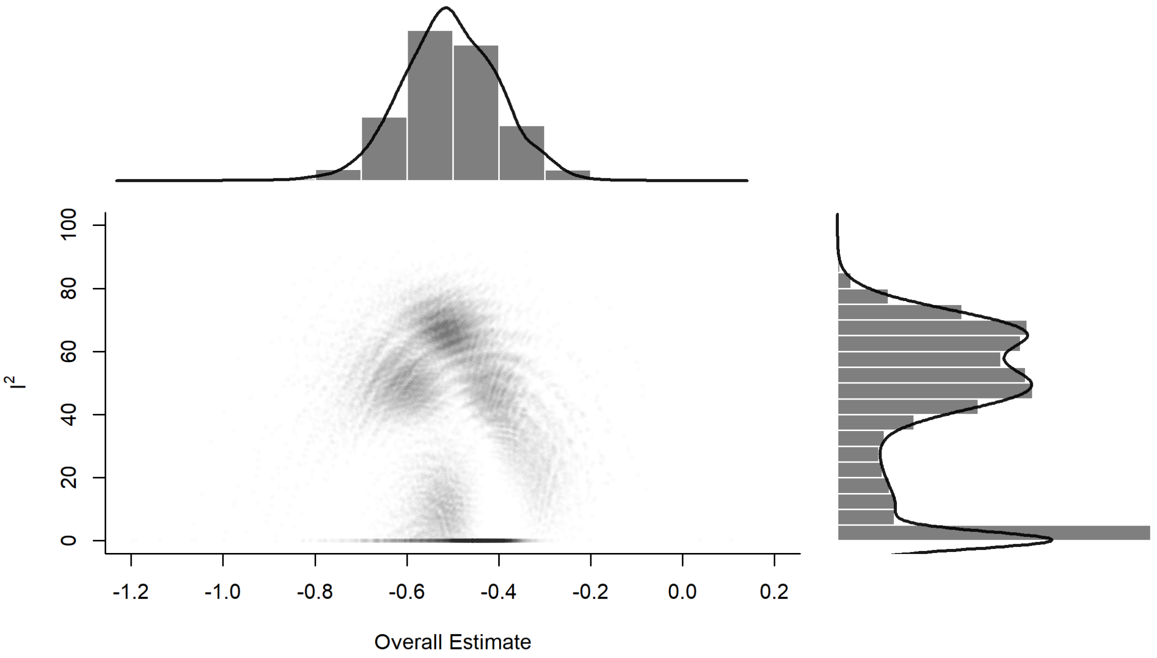

(E)

Note. A: Baujat plot; B: influence plot; C: leave one out method, influence on effect size; D: leave one out method, influence on heterogeneity; E: Graphic Display of Heterogeneity (GOSH) plot.

Information on how to interpret these plots:

Baujat plot: This plot helps to identify studies that excessively contribute to heterogeneity. It displays a study's impact on both the overall heterogeneity (measured by Cochran's *Q*) and the pooled effect size, determined using the leave-one-out method.

Influence plot: The studies' values on several influence measures are presented.

Leave one out method, influence on effect size: This plot visualizes the results from the Leave-one-out-analyses, it indicates the standardized difference in the overall effect when the study is included versus when it is excluded.

Leave one out method, influence on heterogeneity: This plot visualizes the results from the Leave-one-out-analyses, it indicates the standardized difference in the heterogeneity when the study is included versus when it is excluded.

Graphic Display of Heterogeneity (GOSH) plot: The plot shows the pooled effect size of all 2^{k-1} possible study combinations along the x-axis and the between-study heterogeneity on the y-axis. If the plot displays multiple distinct clusters, indicates the likelihood of multiple populations of effect sizes, calling for a subgroup analysis. Conversely, if the effect sizes are consistent, the GOSH plot will exhibit a uniform and symmetrical distribution.

Figure S13

Results for the influence analyses for perceived health status at post-treatment

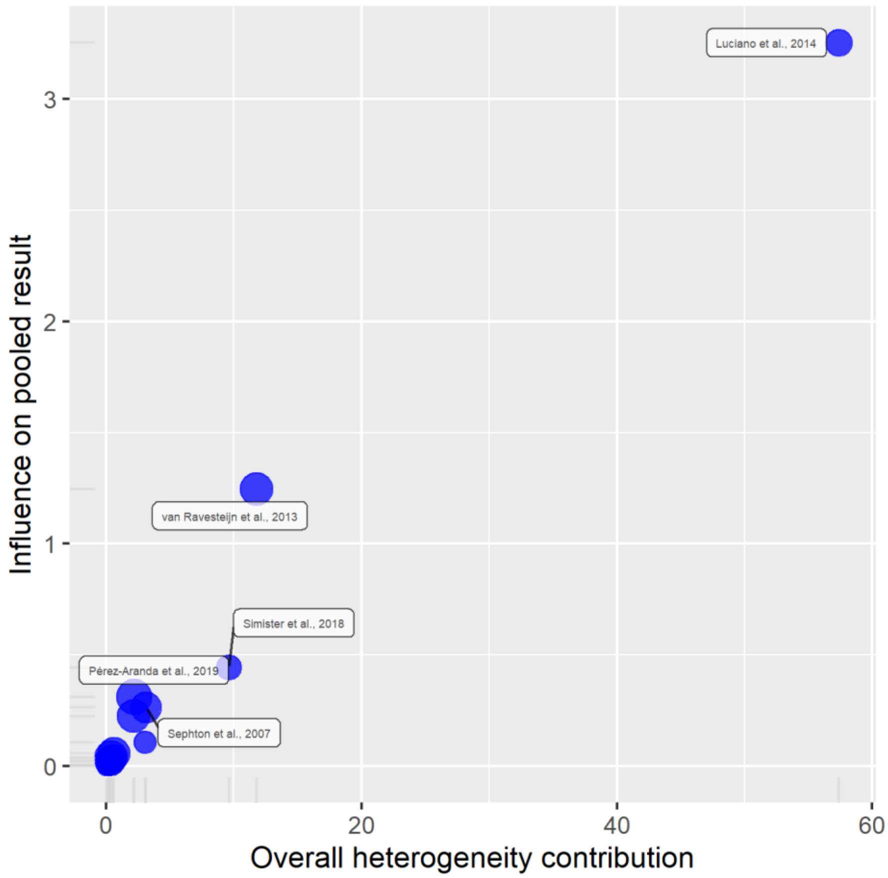

(A)

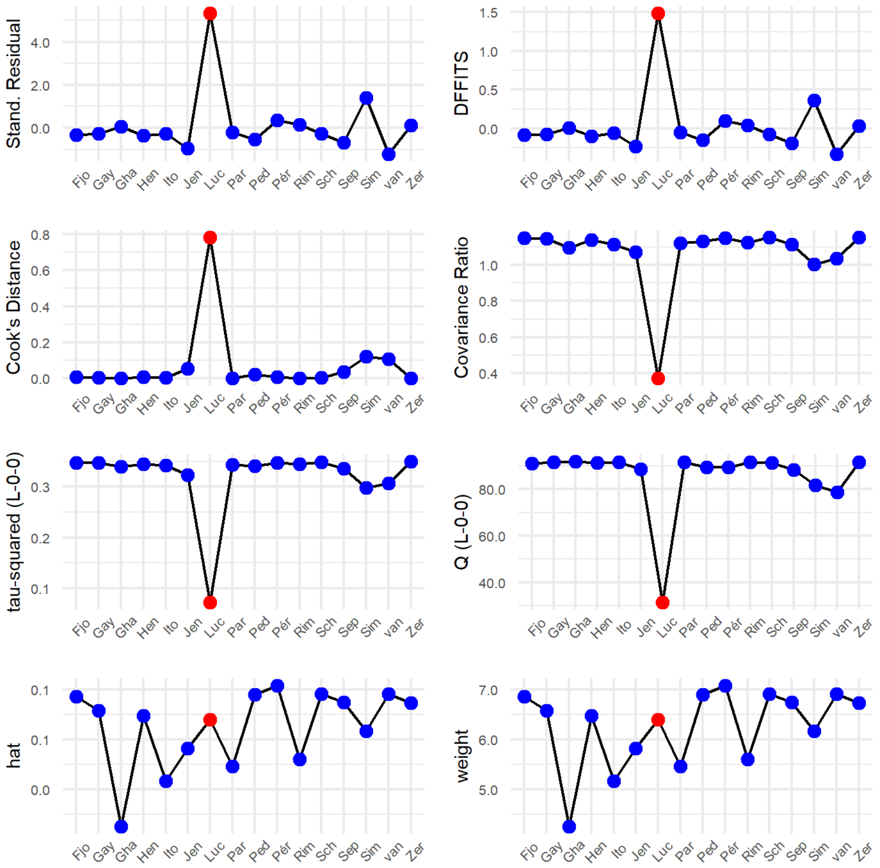
Stand. Residual
4.0
2.0
0.0
DFFITS
1.5
1.0
0.5
0.0
Cook's Distance
0.8
0.6
0.4
0.2
0.0
Covariance Ratio
1.0
0.8
0.6
0.4
tau-squared (L-0-0)
0.3
0.2
0.1
Q (L-0-0)
80.0
60.0
40.0
hat
0.1
0.1
0.0
weight
7.0
6.0
5.0
Fjo Gay Gha Hen Ito Jen Luc Par Ped Pér Rim Sch Sep Sim van Zer

(B)

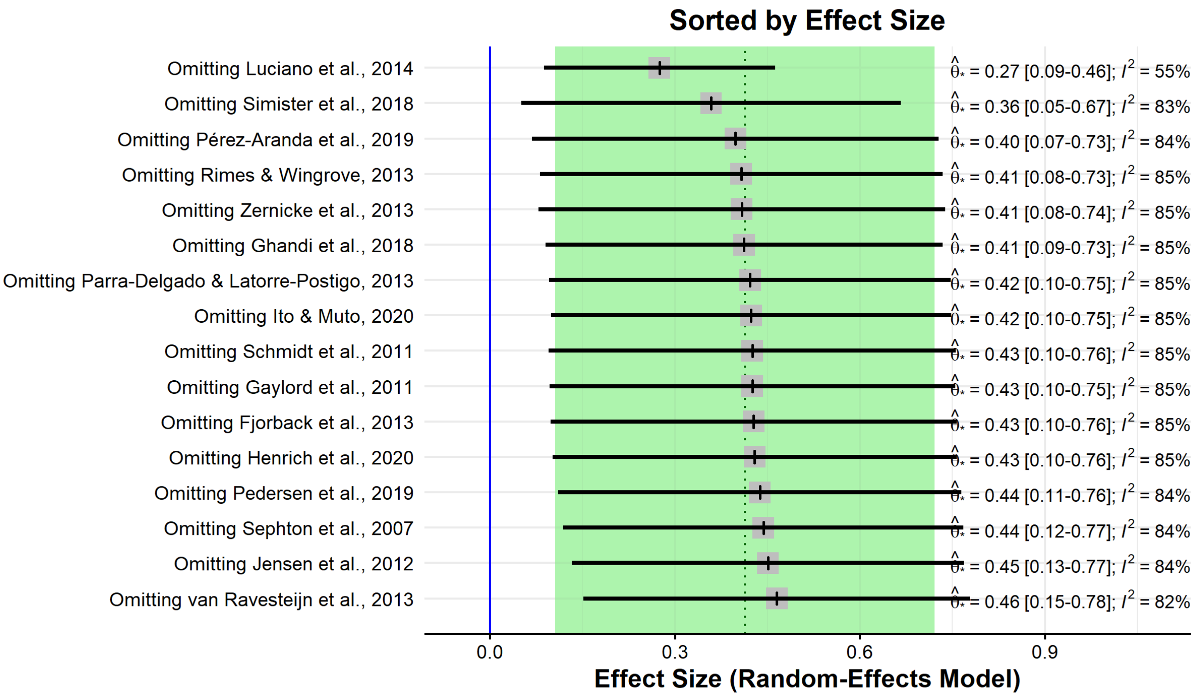
Sorted by Effect Size
Omitting Luciano et al., 2014
Omitting Simister et al., 2018
Omitting Pérez-Aranda et al., 2019
Omitting Rimes & Wingrove, 2013
Omitting Zernicke et al., 2013
Omitting Ghandi et al., 2018
Omitting Parra-Delgado & Latorre-Postigo, 2013
Omitting Ito & Muto, 2020
Omitting Schmidt et al., 2011
Omitting Gaylord et al., 2011
Omitting Fjorback et al., 2013
Omitting Henrich et al., 2020
Omitting Pedersen et al., 2019
Omitting Sephton et al., 2007
Omitting Jensen et al., 2012
Omitting van Ravesteijn et al., 2013
$\hat{\theta}_*$ = 0.27 [0.09-0.46]; I^2 = 55%
$\hat{\theta}_*$ = 0.36 [0.05-0.67]; I^2 = 83%
$\hat{\theta}_*$ = 0.40 [0.07-0.73]; I^2 = 84%
$\hat{\theta}_*$ = 0.41 [0.08-0.73]; I^2 = 85%
$\hat{\theta}_*$ = 0.41 [0.08-0.74]; I^2 = 85%
$\hat{\theta}_*$ = 0.41 [0.09-0.73]; I^2 = 85%
$\hat{\theta}_*$ = 0.42 [0.10-0.75]; I^2 = 85%
$\hat{\theta}_*$ = 0.42 [0.10-0.75]; I^2 = 85%
$\hat{\theta}_*$ = 0.43 [0.10-0.76]; I^2 = 85%
$\hat{\theta}_*$ = 0.43 [0.10-0.75]; I^2 = 85%
$\hat{\theta}_*$ = 0.43 [0.10-0.76]; I^2 = 85%
$\hat{\theta}_*$ = 0.43 [0.10-0.76]; I^2 = 85%
$\hat{\theta}_*$ = 0.44 [0.11-0.76]; I^2 = 84%
$\hat{\theta}_*$ = 0.44 [0.12-0.77]; I^2 = 84%
$\hat{\theta}_*$ = 0.45 [0.13-0.77]; I^2 = 84%
$\hat{\theta}_*$ = 0.46 [0.15-0.78]; I^2 = 82%
0.0
0.3
0.6
0.9
Effect Size (Random-Effects Model)

(C)

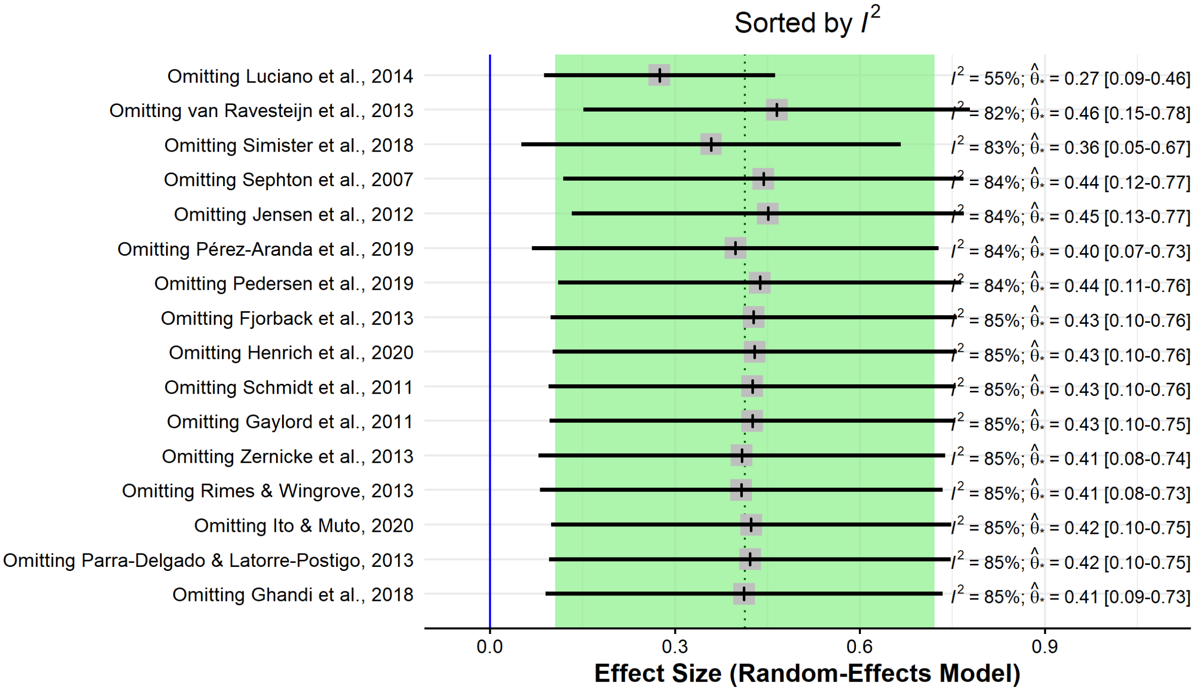

(D)

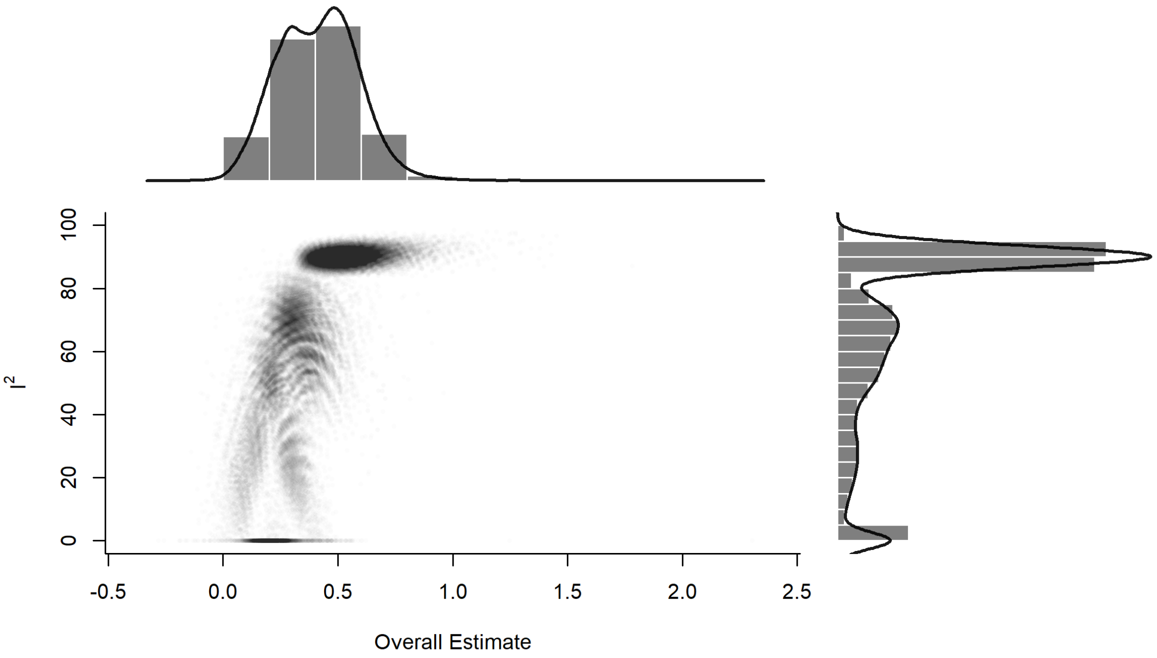

(E)

Note. A: Baujat plot; B: influence plot; C: leave one out method, influence on effect size; D: leave one out method, influence on heterogeneity; E: Graphic Display of Heterogeneity (GOSH) plot. Note: For more information on how to interpret these plots see below Figure S12.

Figure S14

Results for the influence analyses for depression at post-treatment

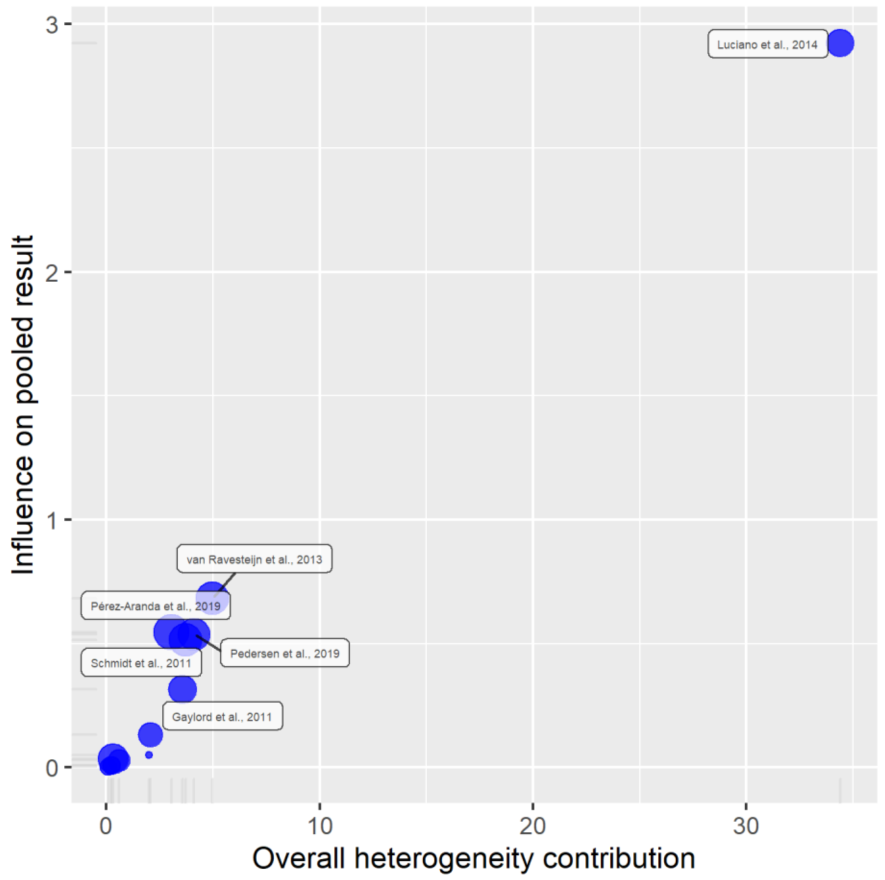

(A)

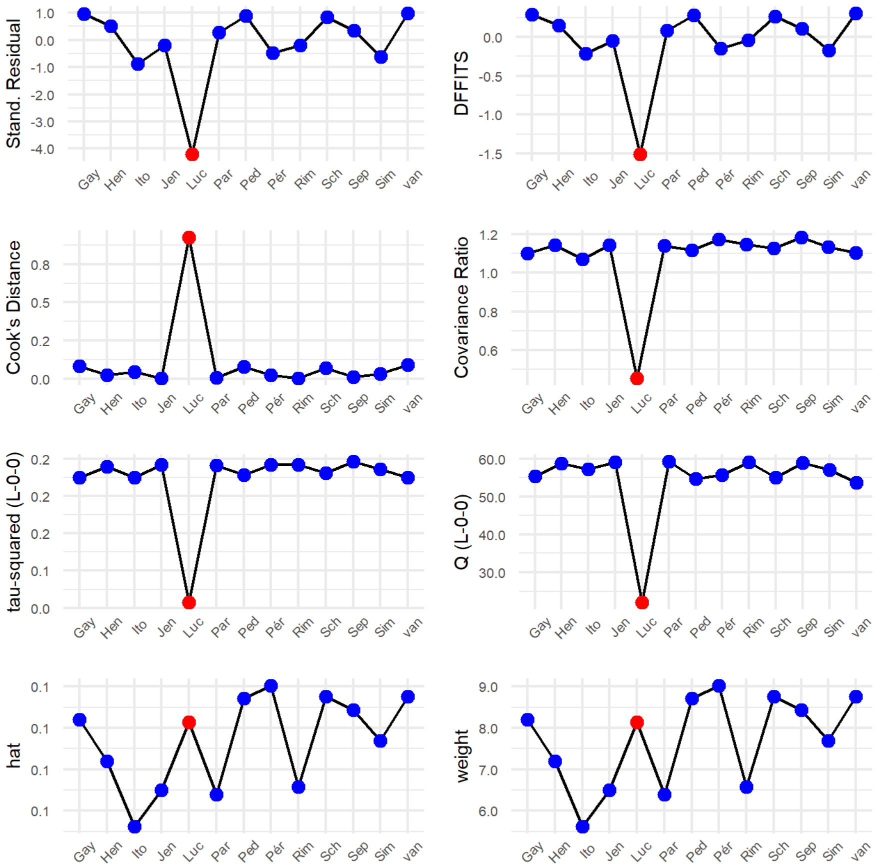
Stand. Residual
DFFITS
Cook's Distance
Covariance Ratio
tau-squared (L-0-0)
Q (L-0-0)
hat
weight
Gay Hen Ito Jen Luc Par Ped Pér Rim Sch Sep Sim van

(B)

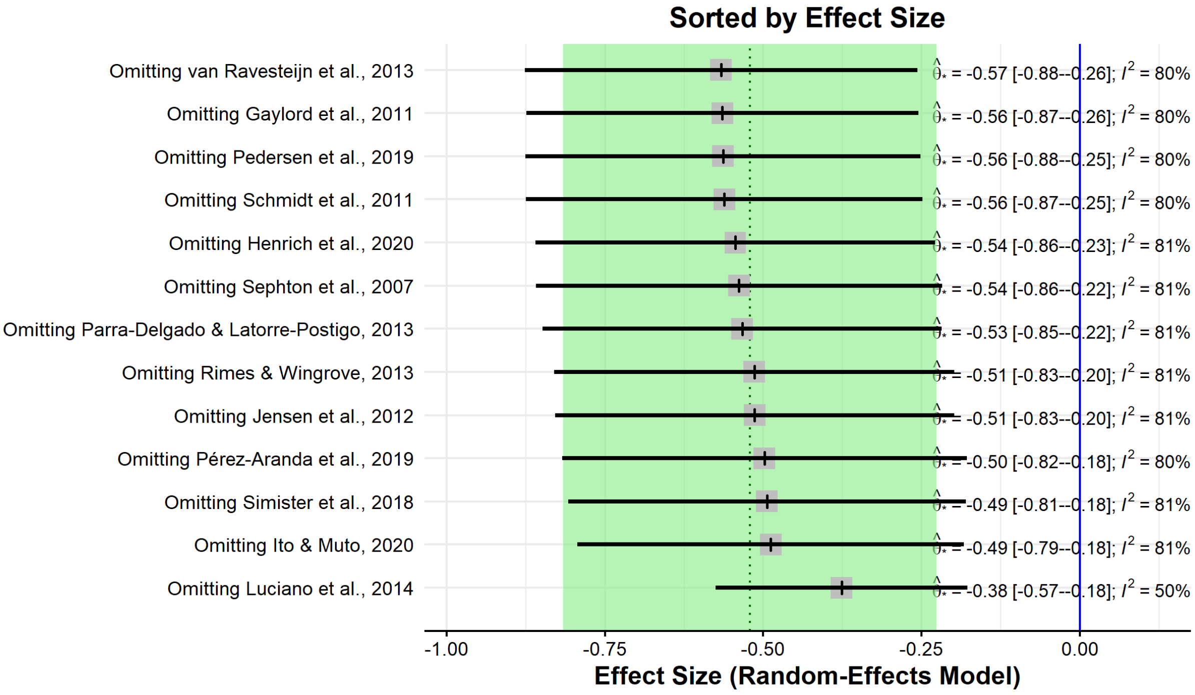
Sorted by Effect Size
Omitting van Ravesteijn et al., 2013
Omitting Gaylord et al., 2011
Omitting Pedersen et al., 2019
Omitting Schmidt et al., 2011
Omitting Henrich et al., 2020
Omitting Sephton et al., 2007
Omitting Parra-Delgado & Latorre-Postigo, 2013
Omitting Rimes & Wingrove, 2013
Omitting Jensen et al., 2012
Omitting Pérez-Aranda et al., 2019
Omitting Simister et al., 2018
Omitting Ito & Muto, 2020
Omitting Luciano et al., 2014
$\hat{\theta}_*$ = -0.57 [-0.88--0.26]; I^2 = 80%
$\hat{\theta}_*$ = -0.56 [-0.87--0.26]; I^2 = 80%
$\hat{\theta}_*$ = -0.56 [-0.88--0.25]; I^2 = 80%
$\hat{\theta}_*$ = -0.56 [-0.87--0.25]; I^2 = 80%
$\hat{\theta}_*$ = -0.54 [-0.86--0.23]; I^2 = 81%
$\hat{\theta}_*$ = -0.54 [-0.86--0.22]; I^2 = 81%
$\hat{\theta}_*$ = -0.53 [-0.85--0.22]; I^2 = 81%
$\hat{\theta}_*$ = -0.51 [-0.83--0.20]; I^2 = 81%
$\hat{\theta}_*$ = -0.51 [-0.83--0.20]; I^2 = 81%
$\hat{\theta}_*$ = -0.50 [-0.82--0.18]; I^2 = 80%
$\hat{\theta}_*$ = -0.49 [-0.81--0.18]; I^2 = 81%
$\hat{\theta}_*$ = -0.49 [-0.79--0.18]; I^2 = 81%
$\hat{\theta}_*$ = -0.38 [-0.57--0.18]; I^2 = 50%
-1.00 -0.75 -0.50 -0.25 0.00
Effect Size (Random-Effects Model)

(C)

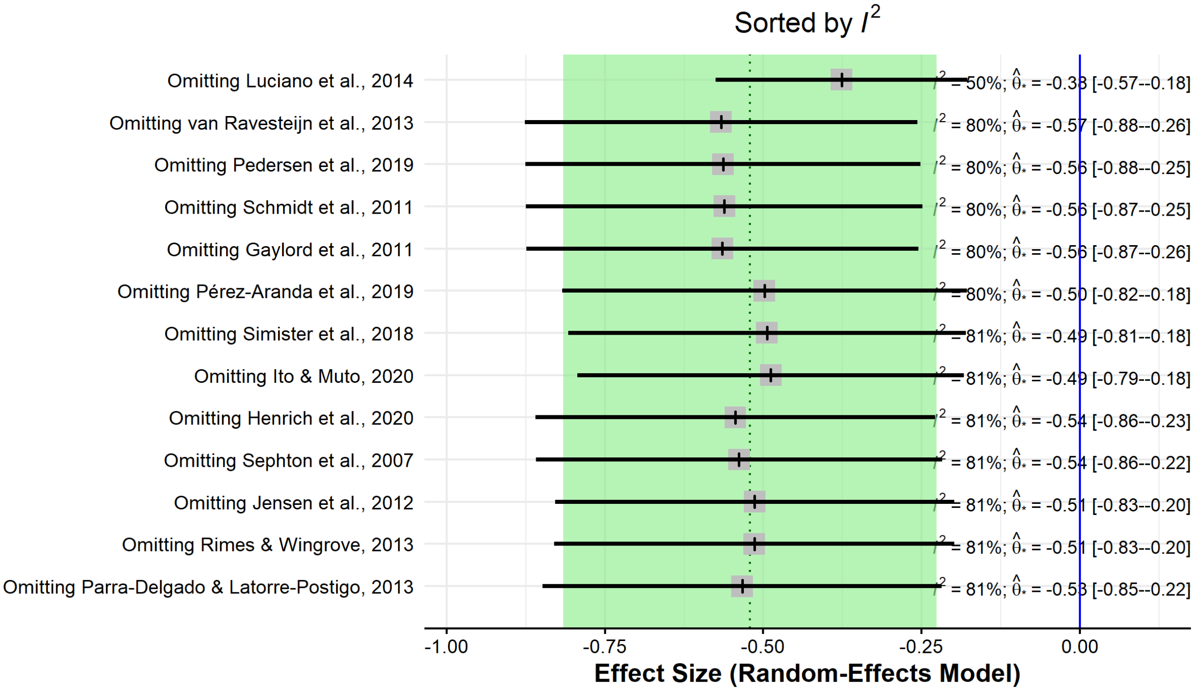

(D)

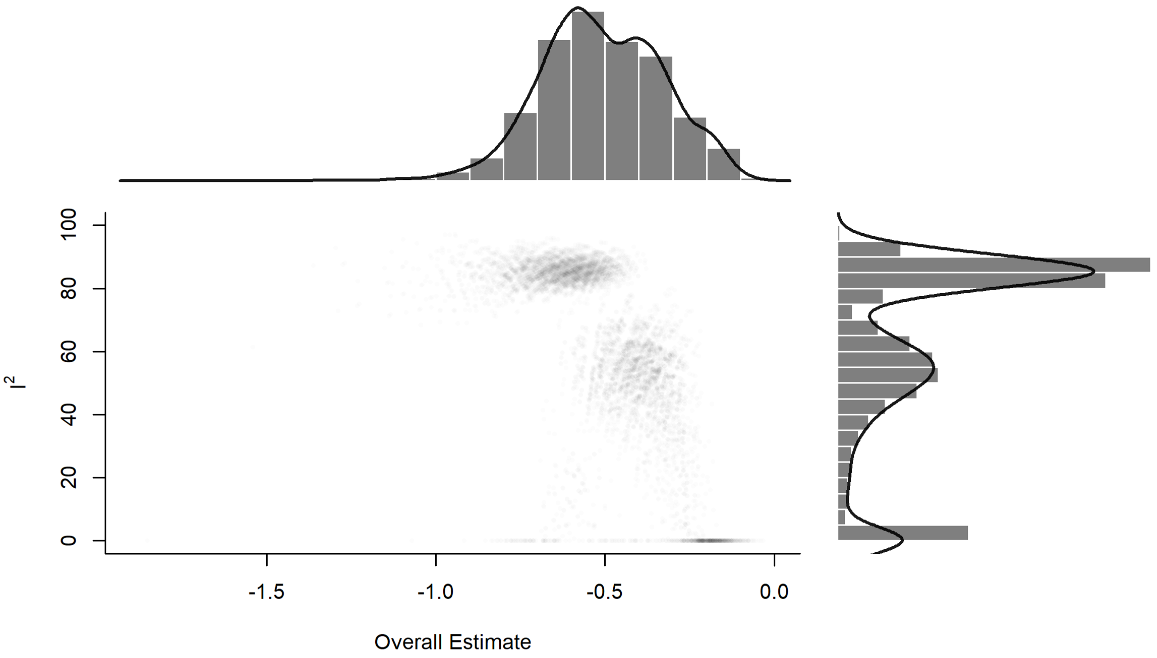

(E)

Note. A: Baujat plot; B: influence plot; C: leave one out method, influence on effect size; D: leave one out method, influence on heterogeneity; E: Graphic Display of Heterogeneity (GOSH) plot. Note: For more information on how to interpret these plots see below Figure S12.

10 Publication Bias

Figure S15

Publication bias – post-treatment

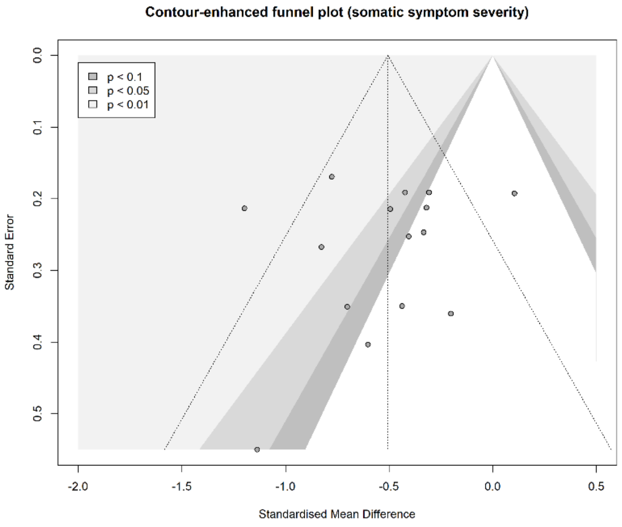

(A)

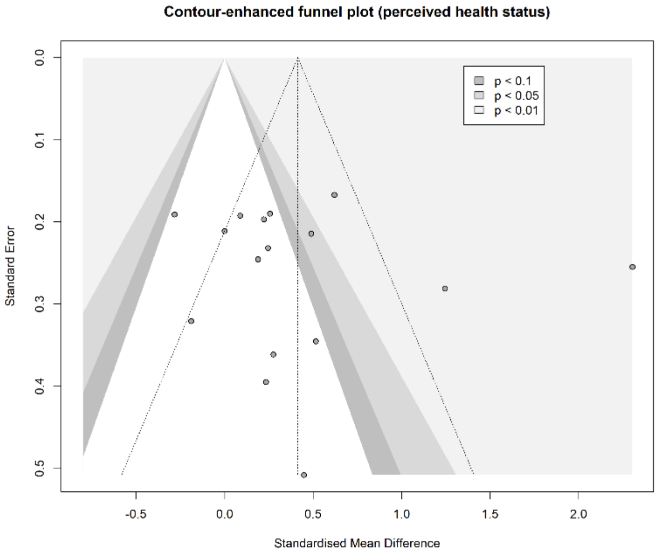

(B)

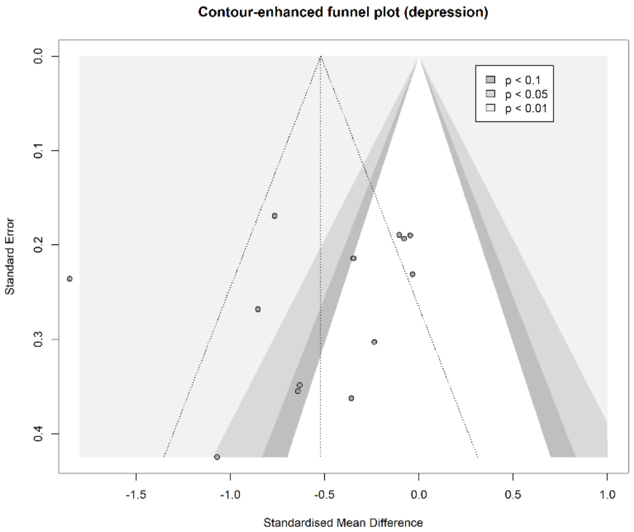
Contour-enhanced funnel plot (depression)
p < 0.1
p < 0.05
p < 0.01
Standard Error
0.0
0.1
0.2
0.3
0.4
-1.5
-1.0
-0.5
0.0
0.5
1.0
Standardised Mean Difference

(C)

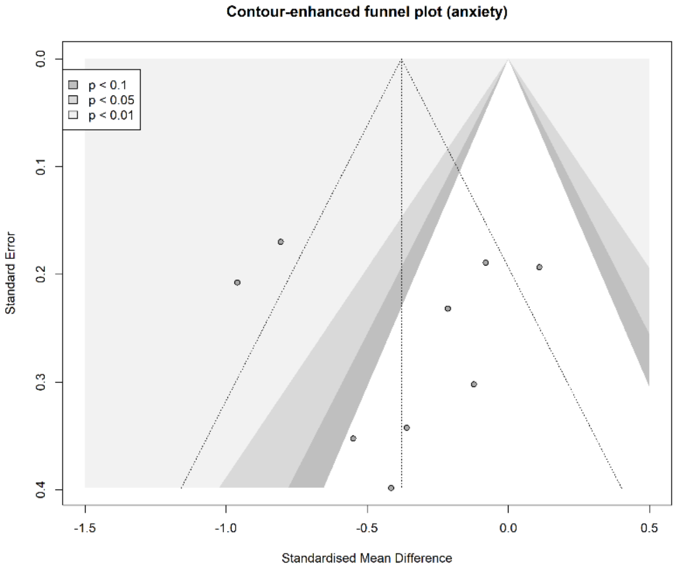
Contour-enhanced funnel plot (anxiety)
p < 0.1
p < 0.05
p < 0.01
Standard Error
0.0
0.1
0.2
0.3
0.4
-1.5
-1.0
-0.5
0.0
0.5
Standardised Mean Difference

(D)

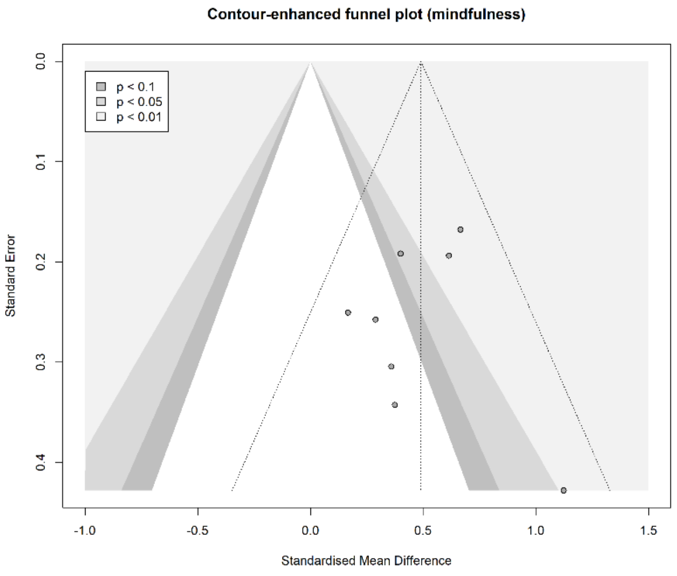

(E)

Note. A: somatic symptom severity at post-treatment; B: perceived health status at post-treatment; C: depression at post-treatment; D: anxiety at post-treatment; E: mindfulness at post-treatment; F: psychological inflexibility at post-treatment

Table S14

Results of Egger's regression tests at post-treatment

Outcome	Intercept	*t*	*p*
Somatic Symptom Severity	-0.71	-0.52	0.612
Perceived Health Status	1.34	0.56	0.579
Depression	-1.85	-0.78	0.449
Anxiety[a]	0.56	0.25	0.811
Mindfulness[a]	-0.22	-0.17	0.873

[a] $k < 10$

10.1 P-curve analyses

Figure S16

P-curve analyses

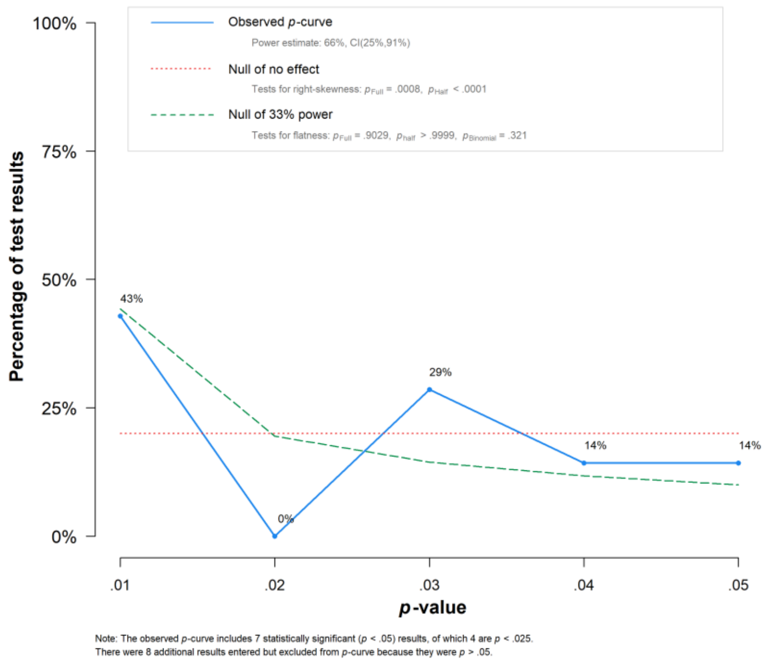

(A)

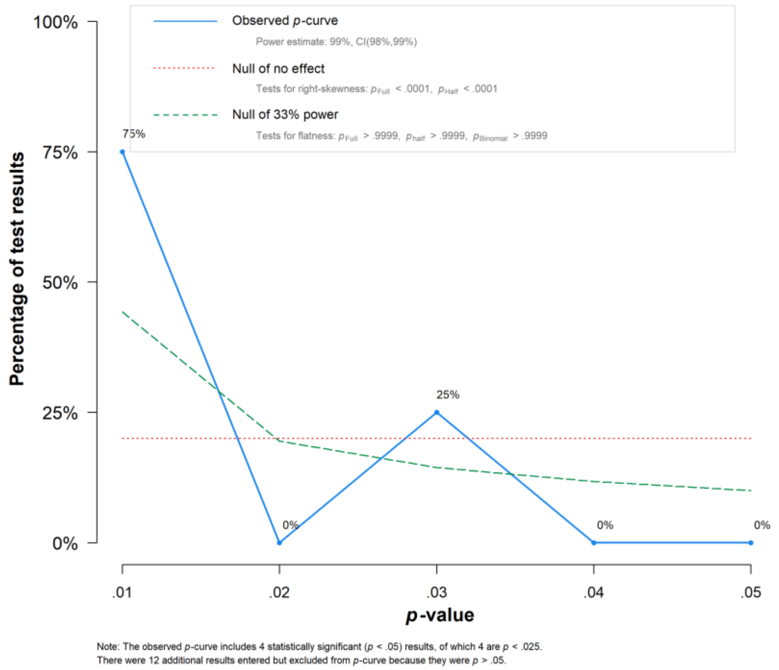

(B)

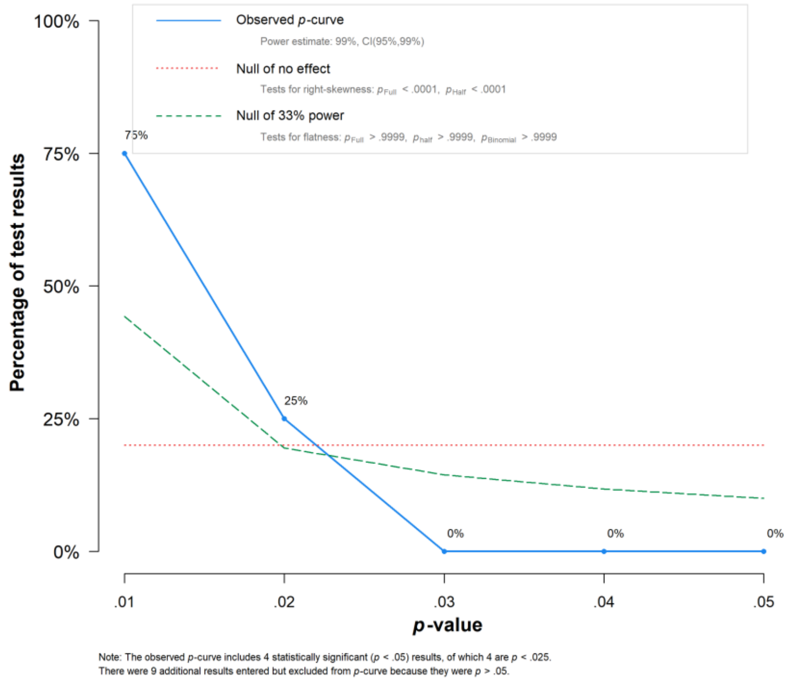

(C)

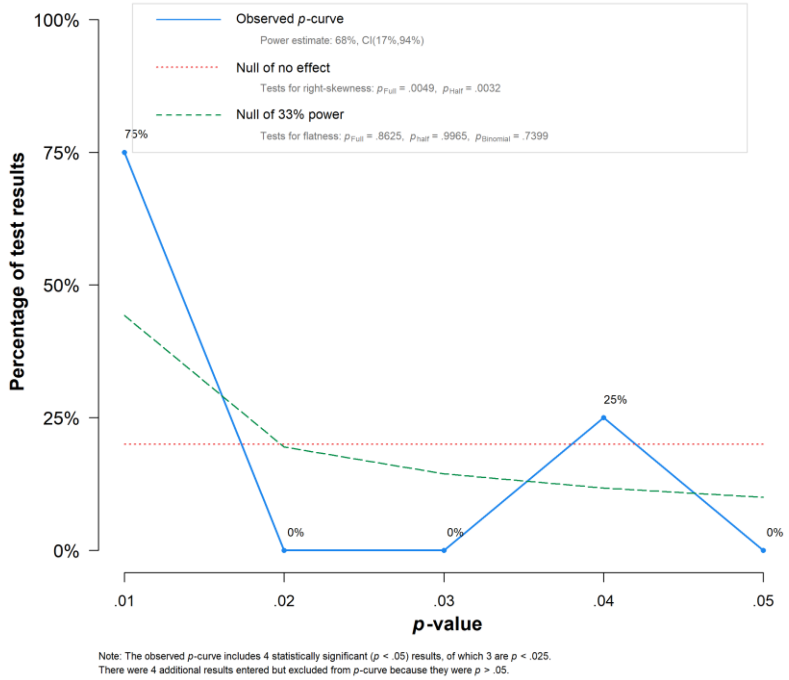

(D)

Note. A: *p*-curve for somatic symptom severity at post-treatment; B: *p*-curve for perceived health status at post-treatment; C: *p*-curve for depression at post-treatment; D: *p*-curve for mindfulness at post-treatment. Note: *p*-curve could not be conducted for anxiety and psychological inflexibility as two or less significant ($p < 0.05$) effect sizes were detected.

11 Literature Cited

1. World Health Organization. The ICD-10 classification of mental and behavioural disorders: Clinical descriptions and diagnostic guidelines. Reprinted. Geneva: World Health Organization; 2009.

2. World Health Organization. ICD-11 - Mortality and Morbidity Statistics: 6C20 Bodily distress disorder; 2020 [cited 2020 May 29]. Available from: URL: https://icd.who.int/browse11/l-m/en#/http://id.who.int/icd/entity/767044268.

3. American Psychiatric Association. Diagnostic criteria from DSM-IV. [Reprint.]. Washington, D.C: American Psychiatric Assoc; 1994.

4. Rief W, Mewes R, Martin A, Glaesmer H, Brähler E. Evaluating new proposals for the psychiatric classification of patients with multiple somatic symptoms. Psychosom Med 2011; 73(9):760–8.

5. American Psychiatric Association. Diagnostic and statistical manual of mental disorders (5th Ed). Arlington, VA: American Psychiatric Publishing; 2013.

6. Rief W, Martin A. How to use the new DSM-5 somatic symptom disorder diagnosis in research and practice: a critical evaluation and a proposal for modifications. Annu Rev Clin Psychol 2014; 10:339–67.

7. Fink PK, Schröder A. One single diagnosis, bodily distress syndrome, succeeded to capture 10 diagnostic categories of functional somatic syndromes and somatoform disorders. J Psychosom Res 2010; 68(5):415–26.

8. Kroenke K, Spitzer RL, deGruy FV, Hahn SR, Linzer M, Williams JBW et al. Multisomatoform disorder. An alternative to undifferentiated somatoform disorder for the somatizing patient in primary care. Arch Gen Psychiatry 1997; 54(4):352–8.

9. Escobar JI, Rubio-Stipec M, Canino G, Karno M. Somatic symptom index (SSI): a new and abridged somatization construct. Prevalence and epidemiological correlates in two large community samples. J Nerv Ment Dis 1989; 177(3):140–6.

10. Kroenke K. Physical symptom disorder: a simpler diagnostic category for somatization-spectrum conditions. J Psychosom Res 2006; 60(4):335–9.

11. Dimsdale JE, Creed FH. The proposed diagnosis of somatic symptom disorders in DSM-V to replace somatoform disorders in DSM-IV--a preliminary report. J Psychosom Res 2009; 66(6):473–6.

12. Creed FH, Guthrie E, Fink PK, Henningsen P, Rief W, Sharpe M et al. Is there a better term than "medically unexplained symptoms"? J Psychosom Res 2010; 68(1):5–8.

13. Burton C, Fink PK, Henningsen P, Löwe B, Rief W. Functional somatic disorders: discussion paper for a new common classification for research and clinical use. BMC Med 2020; 18(1):1–7.

14. Black CJ, Yiannakou Y, Houghton LA, Ford AC. Epidemiological, Clinical, and Psychological Characteristics of Individuals with Self-reported Irritable Bowel Syndrome Based on the Rome IV vs Rome III Criteria. Clin Gastroenterol Hepatol 2020; 18(2):392-398.e2.

15. Layer P, Andresen V, Pehl C, Allescher H, Bischoff SC, Claßen M et al. S3-Leitlinie Reizdarmsyndrom: Definition, Pathophysiologie, Diagnostik und Therapie. Gemeinsame

Leitlinie der Deutschen Gesellschaft für Verdauungs- und Stoffwechselkrankheiten (DGVS) und der Deutschen Gesellschaft für Neurogastroenterologie und Motilität (DGNM)1. Z Gastroenterol 2011; 49(2):237–93.

16. Holmes GP, Kaplan JE, Gantz NM, Komaroff AL, Schonberger LB, Straus SE et al. Chronic fatigue syndrome: a working case definition. Annals of Internal Medicine 1988; 108(3):387–9.

17. Sharpe MC, Archard LC, Banatvala JE, Borysiewicz LK, Clare AW, David A et al. A report - chronic fatigue syndrome: guidelines for research. J R Soc Med 1991; 84(2):118–21.

18. Fukuda K, Straus SE, Hickie I, Sharpe M, Dobbins JG, Komaroff A. The chronic fatigue syndrome: a comprehensive approach to its definition and study. International Chronic Fatigue Syndrome Study Group. Annals of Internal Medicine 1994; 121(12):953–9.

19. Carruthers BM, Jain AK, Meirleir KL de, Peterson DL, Klimas NG, Lerner AM et al. Myalgic Encephalomyelitis/Chronic Fatigue Syndrome: Clinical Working Case Definition, Diagnostic and Treatment Protocols. Journal of Chronic Fatigue Syndrome 2003; 11(1):7–115.

20. Carruthers BM, van de Sande MI, Meirleir KL de, Klimas NG, Broderick G, Mitchell T et al. Myalgic encephalomyelitis: International Consensus Criteria. J Intern Med 2011; 270(4):327–38.

21. National Institute for Health and Clinical Excellence. Chronic fatigue syndrome/myalgic encephalomyelitis (or encephalopathy): Diagnosis and management of CFS/ME in adults and children. London: National Institute for Clinical Excellence; 2007. (NICE clinical guideline; vol 53).

22. Institute of Medicine. Beyond myalgic encephalomyelitis/chronic fatigue syndrome: Redefining an illness. Washington, District of Columbia: The National Academies Press; 2015.

23. Clayton EW. Beyond myalgic encephalomyelitis/chronic fatigue syndrome: an IOM report on redefining an illness. JAMA 2015; 313(11):1101–2. Available from: URL: https://pubmed.ncbi.nlm.nih.gov/25668027/.

24. Wolfe F, Smythe HA, Yunus MB, Bennett RM, Bombardier C, Goldenberg DL et al. The American College of Rheumatology 1990 Criteria for the Classification of Fibromyalgia. Report of the Multicenter Criteria Committee. Arthritis Rheum 1990; 33(2):160–72.

25. Wolfe F, Clauw DJ, Fitzcharles M-A, Goldenberg DL, Katz RS, Mease P et al. The American College of Rheumatology preliminary diagnostic criteria for fibromyalgia and measurement of symptom severity. Arthritis Care & Research 2010; 62(5):600–10. Available from: URL: https://pubmed.ncbi.nlm.nih.gov/20461783/.

26. Wolfe F, Clauw DJ, Fitzcharles M-A, Goldenberg DL, Häuser W, Katz RL et al. 2016 Revisions to the 2010/2011 fibromyalgia diagnostic criteria. Seminars in Arthritis and Rheumatism 2016; 46(3):319–29. Available from: URL: http://www.sciencedirect.com/science/article/pii/S0049017216302086.

27. Häuser W, Perrot S, Sommer C, Shir Y, Fitzcharles M-A. Diagnostic confounders of chronic widespread pain: not always fibromyalgia. Pain Rep 2017; 2(3):e598.

28. Amutio A, Franco C, Pérez-Fuentes MdC, Gázquez JJ, Mercader I. Mindfulness training for reducing anger, anxiety, and depression in fibromyalgia patients. Front Psychol 2014; 5:1572.

29. Pérez-Aranda A, Feliu-Soler A, Montero-Marín J, García-Campayo J, Andrés-Rodríguez L, Borràs X et al. A randomized controlled efficacy trial of mindfulness-based stress reduction compared with an active control group and usual care for fibromyalgia: the EUDAIMON study. Pain 2019; 160(11):2508–23.

30. Givehki R, Afshar H, Goli F, Scheidt CE, Omidi A, Davoudi M. Effect of acceptance and commitment therapy on body image flexibility and body awareness in patients with psychosomatic disorders: a randomized clinical trial. Electron Physician 2018; 10(7):7008–16.

31. Mirsharifa SM, Mirzaian B, Dousti Y. The Efficacy of Acceptance and Commitment Therapy (ACT) Matrix on Depression and Psychological Capital of the Patients with Irritable Bowel Syndrome. Open Access Maced J Med Sci 2019; 7(3):421–7.

32. Mohamadi J, Ghazanfari F, Drikvand FM. Comparison of the Effect of Dialectical Behavior Therapy, Mindfulness Based Cognitive Therapy and Positive Psychotherapy on Perceived Stress and Quality of Life in Patients with Irritable Bowel Syndrome: a Pilot Randomized Controlled Trial. Psychiatr Q 2019; 90(3):565–78.

33. Zomorrodi S, Rasoulzadeh Tabatabaie SK, Azadfallah P, Ebrahimidaryani N, Arbabi M. Long Term Effects of Mindfulness on Quality of life in Irritable Bowel Syndrome. Iran J Psychiatry 2015; 10(2):100–5.

34. Ljótsson B, Andersson G, Andersson E, Hedman E, Lindfors P, Andréewitch S et al. Acceptability, effectiveness, and cost-effectiveness of internet-based exposure treatment for irritable bowel syndrome in a clinical sample: a randomized controlled trial. BMC Gastroenterol 2011; 11:110.

35. Ljótsson B, Hesser H, Andersson E, Lackner JM, El Alaoui S, Falk L et al. Provoking symptoms to relieve symptoms: a randomized controlled dismantling study of exposure therapy in irritable bowel syndrome. Behaviour Research and Therapy 2014; 55:27–39.

36. Kleinstäuber M, Gottschalk J, Berking M, Rau J, Rief W. Enriching Cognitive Behavior Therapy with Emotion Regulation Training for Patients with Multiple Medically Unexplained Symptoms (ENCERT): Design and implementation of a multicenter, randomized, active-controlled trial. Contemp Clin Trials 2016; 47:54–63.

37. Borenstein M, Hedges LV, Higgins JPT, Rothstein HR. Introduction to meta-analysis. Reprinted. Chichester: Wiley; 2010. Available from: URL: http://www.Meta-Analysis.com.

38. Cuijpers P. Meta-analyses in mental health research: A practical guide. Amsterdam: Pim Cuijpers Uitgeverij; 2016.

39. Rustenbach SJ. Metaanalyse: Eine anwendungsorientierte Einführung [Meta-analysis: an practical introduction] [Zugl.: Hamburg, Univ., Diss., 2003]. 1. Aufl. Bern, Göttingen: Huber; 2003. (Methoden der Psychologie; vol 16).

40. Parra-Delgado M, Latorre-Postigo JM. Effectiveness of Mindfulness-Based Cognitive Therapy in the Treatment of Fibromyalgia: A Randomised Trial. Cogn Ther Res 2013; 37(5):1015–26.

41. Hoyt WT, Del Re AC. Effect size calculation in meta-analyses of psychotherapy outcome research. Psychotherapy Research 2018; 28(3):379–88.

42. Wampold BE, Mondin GW, Moody M, Stich F, Benson K, Ahn H-n. A meta-analysis of outcome studies comparing bona fide psychotherapies: Empiricially, "all must have prizes.". Psychological Bulletin 1997; 122(3):203–15.

43. Jensen KB, Kosek E, Wicksell R, Kemani M, Olsson G, Merle JV et al. Cognitive Behavioral Therapy increases pain-evoked activation of the prefrontal cortex in patients with fibromyalgia. Pain 2012; 153(7):1495–503.

44. Ito M, Muto T. Effectiveness of acceptance and commitment therapy for irritable bowel syndrome non-patients: A pilot randomized waiting list controlled trial. Journal of Contextual Behavioral Science 2020; 15:85–91.

45. Pedersen HF, Agger JL, Frostholm L, Jensen JS, Ørnbøl E, Fink P et al. Acceptance and Commitment group Therapy for patients with multiple functional somatic syndromes: a three-armed trial comparing ACT in a brief and extended version with enhanced care. Psychol. Med. 2019; 49(6):1005–14.

46. Schmidt S, Grossman P, Schwarzer B, Jena S, Naumann J, Walach H. Treating fibromyalgia with mindfulness-based stress reduction: results from a 3-armed randomized controlled trial. Pain 2011; 152(2):361–9.

47. van Ravesteijn H, Lucassen P, Bor H, van Weel C, Speckens A. Mindfulness-based cognitive therapy for patients with medically unexplained symptoms: a randomized controlled trial. PPS 2013; 82(5):299–310.

48. Luciano JV, Guallar JA, Aguado J, López-Del-Hoyo Y, Olivan B, Magallón R et al. Effectiveness of group acceptance and commitment therapy for fibromyalgia: a 6-month randomized controlled trial (EFFIGACT study). Pain 2014; 155(4):693–702.

49. Gold SM, Enck P, Hasselmann H, Friede T, Hegerl U, Mohr DC et al. Control conditions for randomised trials of behavioural interventions in psychiatry: a decision framework. The Lancet Psychiatry 2017; 4(9):725–32.

50. R: A language and environment for statistical computing. Version 4.2.1. Vienna, Austria: R Foundation for Statistical Computing; 2022. Available from: URL: https://www.R-project.org/.

51. Balduzzi S, Rücker G, Schwarzer G. How to perform a meta-analysis with R: a practical tutorial. Evid Based Ment Health 2019; 22(4):153–60.

52. Viechtbauer W. Conducting Meta-Analyses in R with the metafor Package. J Stat Softw 2010; 36(3).

53. dmetar: Companion R Package For The Guide 'Doing Meta-Analysis in. Version R package version 0.0.9000; 2019. Available from: URL: http://dmetar.protectlab.org/.

54. Wickham H, Averick M, Bryan J, Chang W, McGowan L, François R et al. Welcome to the Tidyverse. JOSS 2019; 4(43):1686.

55. Lüdecke D. Effect Size Computation for Meta Analysis [R package esc version 0.5.1]. Comprehensive R Archive Network (CRAN); 2019. Available from: URL: https://cran.r-project.org/web/packages/esc/index.html.

56. An R Companion to Applied Regression. Version Third edition. Thousand Oaks, CA, USA: Sage; 2019. Available from: URL: https://socialsciences.mcmaster.ca/jfox/Books/Companion/.

57. readxl: Read Excel Files. Version 1.4.0; 2022. Available from: URL: https://CRAN.R-project.org/package=readxl.

58. Microsoft Excel. Version Microsoft® Excel® für Microsoft 365 MSO (Version 2205 Build 16.0.15225.20028) 32 Bit; 2018.

59. McGuinness LA, Higgins JPT. Risk-of-bias VISualization (robvis): An R package and Shiny web app for visualizing risk-of-bias assessments. Res Synth Methods 2021; 12(1):55–61.

60. irr: Various Coefficients of Interrater Reliability and Agreement. Version 0.84.1; 2019. Available from: URL: https://CRAN.R-project.org/package=irr.

61. Simister HD, Tkachuk GA, Shay BL, Vincent N, Pear JJ, Skrabek RQ. Randomized Controlled Trial of Online Acceptance and Commitment Therapy for Fibromyalgia. J Pain 2018; 19(7):741–53.
